# Diagnostic Accuracy of Artificial Intelligence for Dental Caries Detection Across Clinical Imaging Modalities: A Systematic Review and Descriptive Synthesis

**DOI:** 10.3390/diagnostics16182995

**Published:** 2026-09-16

**Authors:** Alain Manuel Chaple Gil, Iván Claudio Suazo Galdames, Laura Pereda Vázquez, Meylin Santiesteban Velázquez, Jorge J. Menendez

**Affiliations:** 1 Facultad de Ciencias de la Salud, Universidad Autónoma de Chile, Santiago 8900000, Chile; 2 Vicerrectoría de Investigación y Doctorados, Universidad Autónoma de Chile, Santiago 7500912, Chile; 3 Penn Dental Medicine, University of Pennsylvania, Philadelphia, PA 19104, USA; 4 Facultad de Odontología, Universidad Autónoma del Paraguay, Asunción 1255, Paraguay; 5 College of Dental Medicine, Nova Southeastern University, Davie, FL 33314, USA

**Keywords:** artificial intelligence, dental caries, diagnostic test accuracy, external validation, GRADE-DTA, sensitivity and specificity, systematic review

## Abstract

**Background/Objectives:** Published estimates of artificial-intelligence (AI) accuracy for dental caries differ in imaging modality, lesion threshold, observational unit, reference standard, and validation design. We mapped the clinical composition and credibility of this evidence and summarized clinician-plus-AI studies separately. **Methods:** Five databases were searched through 10 June 2026. Eligible diagnostic-accuracy studies used clinically acquired human dental data. QUADAS-3 and a structured GRADE-DTA assessment were applied. Because the studies did not address a common clinical question, no pooled operating point was estimated. **Results:** A total of 29 reports representing 28 studies were included. Overall risk of bias was high for all 28 estimates; only five studies used external validation. Twelve standalone-AI reports supplied exact, coherent 2 × 2 data. Their sensitivity ranged from 0.360 to 0.940 and specificity from 0.700 to 0.983. The exact-data subset was an availability sample, and most nominal uncertainty estimates could not account for clustering. Two controlled reader studies reported higher sensitivity with AI assistance, accompanied by lower specificity in one study and more invasive treatment decisions in the other. Certainty was very low for both standalone accuracy and incremental clinician benefit. **Conclusions:** The evidence does not support a transferable accuracy benchmark or a conclusion of net clinical benefit. Locked-model external validation and paired prospective evaluations with patient-relevant outcomes are needed.

## 1. Introduction

Artificial-intelligence systems for dental caries detection have been evaluated using bitewing, periapical, and panoramic radiographs, cone-beam computed tomography, intraoral photographs, fluorescence images, and three-dimensional scans. Systematic reviews and primary diagnostic-accuracy studies have reported promising but variable sensitivity and specificity [1,2,3,4,5].

Clinical interpretation is limited because the studies differ in modality, lesion threshold, observational unit, reference standard, sampling frame, and validation design. Accuracy for enamel-only proximal lesions on bitewings is not exchangeable with tooth-level panoramic classification, photograph-based cavitation detection, or patch-level computational tasks.

Standalone algorithm accuracy and clinician performance with AI assistance are also different questions [6,7,8]. The first evaluates algorithm output against a reference standard; the second asks whether AI changes human interpretation, errors, decisions, or outcomes. Cost-effectiveness and patient benefit require further evidence beyond diagnostic discrimination.

This review therefore maps the clinical composition, methodological limitations, external validation, and study-level accuracy of the available evidence. Reports with exact, internally coherent 2 × 2 data are shown as a descriptive subset rather than pooled across non-exchangeable clinical questions. Controlled clinician-plus-AI evidence is summarized separately.

### 1.1. Research Questions

Primary question: What study-level diagnostic-accuracy evidence is reported for standalone AI systems that detect dental caries in clinically acquired human dental data?

Secondary question: What controlled evidence evaluates changes in clinician performance when AI assistance is added?

### 1.2. Objective

To describe the clinical composition, risk of bias, external validation, study-level sensitivity and specificity, and certainty of evidence for standalone AI caries detection, and to summarize controlled clinician-plus-AI evidence separately.

## 2. Materials and Methods

### 2.1. Study Design and Registration

This systematic review of diagnostic test accuracy was conducted and reported in accordance with PRISMA 2020 and PRISMA-DTA [9,10]. The protocol was prospectively registered in PROSPERO (CRD420251232014) before title/abstract screening and data extraction.

### 2.2. Eligibility Criteria

Eligible studies evaluated a defined standalone AI test or clinician interpretation with AI assistance on clinically acquired human dental data against a study-defined clinical, operative, histological, or expert image-based reference standard and reported at least one quantitative diagnostic result. The target condition and observational unit were recorded as defined by each study.

Reports were retained in the systematic review even when a complete contingency table was unavailable. A descriptive exact-data subset required integer TP, FP, FN, and TN values reported directly or uniquely reconstructable from internally consistent denominators and unrounded measures, together with a clinically interpretable patient, tooth, surface, or complete-image unit. Non-integer pseudocells, incompatible denominators, patch-only units, and unresolved dependent computational subsamples were excluded from this subset.

Animal, phantom, simulated, and purely in vitro studies were excluded, as were reports without a caries outcome, defined reference standard, or quantitative diagnostic result, and non-original publication types. Standalone AI and clinician-plus-AI interpretation were treated as separate index-test constructs.

### 2.3. Information Sources and Search Strategy

PubMed, Scopus, Web of Science Core Collection, Embase, and the Cochrane Library were searched through 10 June 2026 without database-level publication-type, study-design, subject-area, category, or disease-mapping restrictions. Exact syntax, interfaces, dates, coverage, and raw yields are provided in Supplementary File S1.

IEEE Xplore was listed in the protocol but was not searched because the required institutional interface/API was unavailable. Trial registers, preprint servers, organizational websites, and other gray-literature sources were not searched, and authors were not contacted for missing cells. These choices may have reduced coverage and were incorporated into the certainty assessment.

### 2.4. Study Selection Criteria

Database exports were imported into Rayyan (Systems, Inc., Cambridge, MA, USA) [11]. Before human screening, the review team applied deterministic rules using bibliographic fields, record types, keyword searches, labels, exclusion reasons, and bulk actions. Rule families targeted clearly non-primary publication types and records whose indexed fields or title/abstract placed them outside dental-caries diagnostic evaluation. Rayyan relevance ranking and AI suggestions were not used.

This process marked 130,245 records ineligible before duplicate human screening. The retained audit trail does not contain the complete bulk-excluded record set, rule-specific counts, or a human-screened validation sample. Its false-negative rate, therefore, cannot be estimated. Records with ambiguous metadata or possible relevance were intended to be retained, but this safeguard cannot substitute for a validation audit.

LPV and MSV independently screened the remaining 3248 titles/abstracts and assessed the 35 retrieved full texts. Disagreements were resolved through discussion, with JM available for adjudication. The blinded pre-consensus export was not retained; Cohen’s kappa and percentage agreement cannot be reconstructed, and the screening stage is not independently auditable at the reviewer-decision level.

### 2.5. Data Extraction

Two reviewers used a standardized, piloted form to extract study design, country, setting, modality, target condition, observational unit, reference standard, model, dataset origin, development/test separation, external validation, clinical role, reported diagnostic measures, and exact contingency cells when available. Disagreements were resolved by discussion and, if required, third-reviewer adjudication. Missing cells were not imputed.

Author-reported limitations were retained only as an ancillary verbatim corpus in the Supplementary Materials. They were not treated as diagnostic outcomes, observed implementation effects, or evidence of translational readiness.

### 2.6. Risk of Bias Assessment

QUADAS-3 version 1.2 was applied without modification at the selected-estimate level [12,13]. Separate synthesis questions and ideal-study definitions were specified for standalone AI and clinician-plus-AI interpretation. The pre-populated master assessment was independently checked against the corresponding source reports by ACG and JM after calibration. This was source verification of a common matrix, not two de novo blinded assessments from blank forms. Final judgments were consensus verified; no inter-reviewer reliability statistic is claimed. Complete responses, rationales, source locations, verification records, and final judgments are provided in Supplementary File S4.

### 2.7. Data Synthesis and Certainty of Evidence

#### 2.7.1. Standalone-AI Evidence

Study characteristics and reported diagnostic measures were synthesized descriptively. For the 12 reports with exact, coherent 2 × 2 data, sensitivity and specificity were calculated and displayed as study-level point estimates. No bivariate summary, HSROC curve, likelihood ratio, diagnostic odds ratio, predictive value, or cross-modality pooled estimate was calculated because the reports did not evaluate a common clinical question.

Most source datasets included multiple teeth, surfaces, images, or lesions per participant without cluster-adjusted variance. Exact binomial intervals would therefore imply independence that was not supported by the study designs. The main display consequently emphasizes point estimates and ranges; raw cells and analytic limitations remain available in Supplementary File S3.

#### 2.7.2. Clinician-Plus-AI Evidence

Controlled reader studies were summarized using the authors’ reported without-AI and with-AI results. They were not combined with standalone algorithm estimates or pooled with each other because their reader assignment, case allocation, lesion thresholds, and reported outcomes differed.

#### 2.7.3. Certainty of Evidence

Certainty was assessed for (1) standalone-AI study-level sensitivity and specificity and (2) incremental clinician performance with AI. A GRADE-DTA structure considered risk of bias, inconsistency, indirectness, imprecision, and publication bias. Domain judgments and reasons are provided in Supplementary Table S4.

#### 2.7.4. Reporting Bias

No funnel-plot or asymmetry test was performed because the descriptive exact-data subset was small and clinically heterogeneous. Risk of missing evidence was instead considered qualitatively from the search coverage and the unvalidated bulk-exclusion process.

## 3. Results

### 3.1. Study Selection

The searches identified 137,274 records: PubMed 31,663; Scopus 37,582; Web of Science 13,836; Embase 53,523; and Cochrane Library 670. Rule-based preprocessing marked 130,245 records ineligible, and 3781 duplicates were removed. Of 3248 records screened by two reviewers, 3213 were excluded. All 35 full texts were retrieved; six were excluded (irrelevant outcome, n = 2; unsuitable design, n = 4). A total of 29 reports representing 28 unique studies were included [4,14,15,16,17,18,19,20,21,22,23,24,25,26,27,28,29,30,31,32,33,34,35,36,37,38,39,40,41].

**Figure 1:**
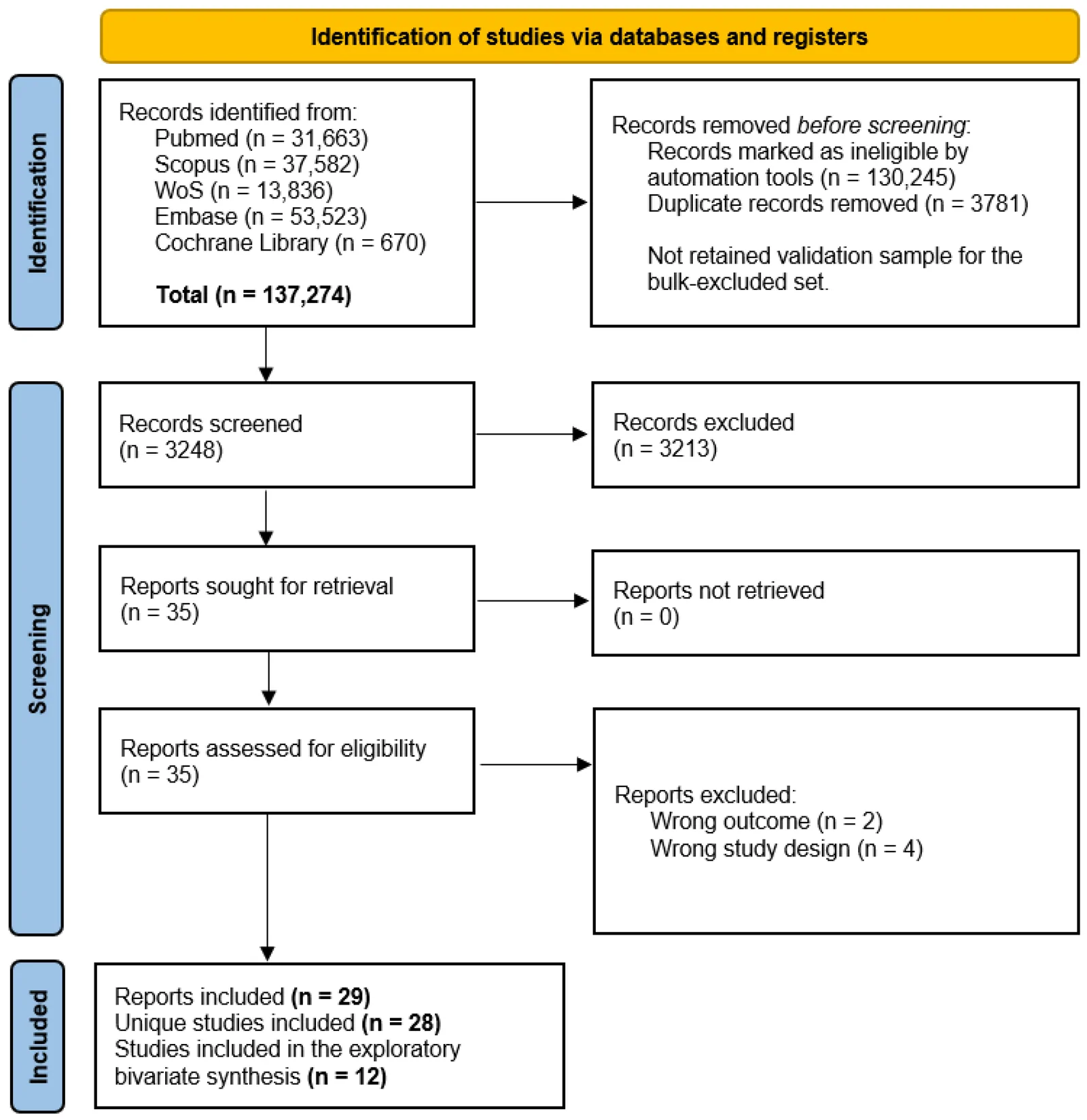
PRISMA 2020 flow diagram. The rule-based preprocessing step marked 130,245 records ineligible; no retained validation sample permits estimation of its false-negative rate. A total of 29 reports represented 28 unique studies; 12 standalone-AI reports supplied exact, coherent 2 × 2 data for descriptive study-level estimates.

The automation box in Figure 1 follows the PRISMA 2020 flow structure, but the magnitude of the bulk exclusion and absence of a retained validation sample are review-level limitations rather than routine deduplication.

### 3.2. Risk of Bias

**Figure 2:**
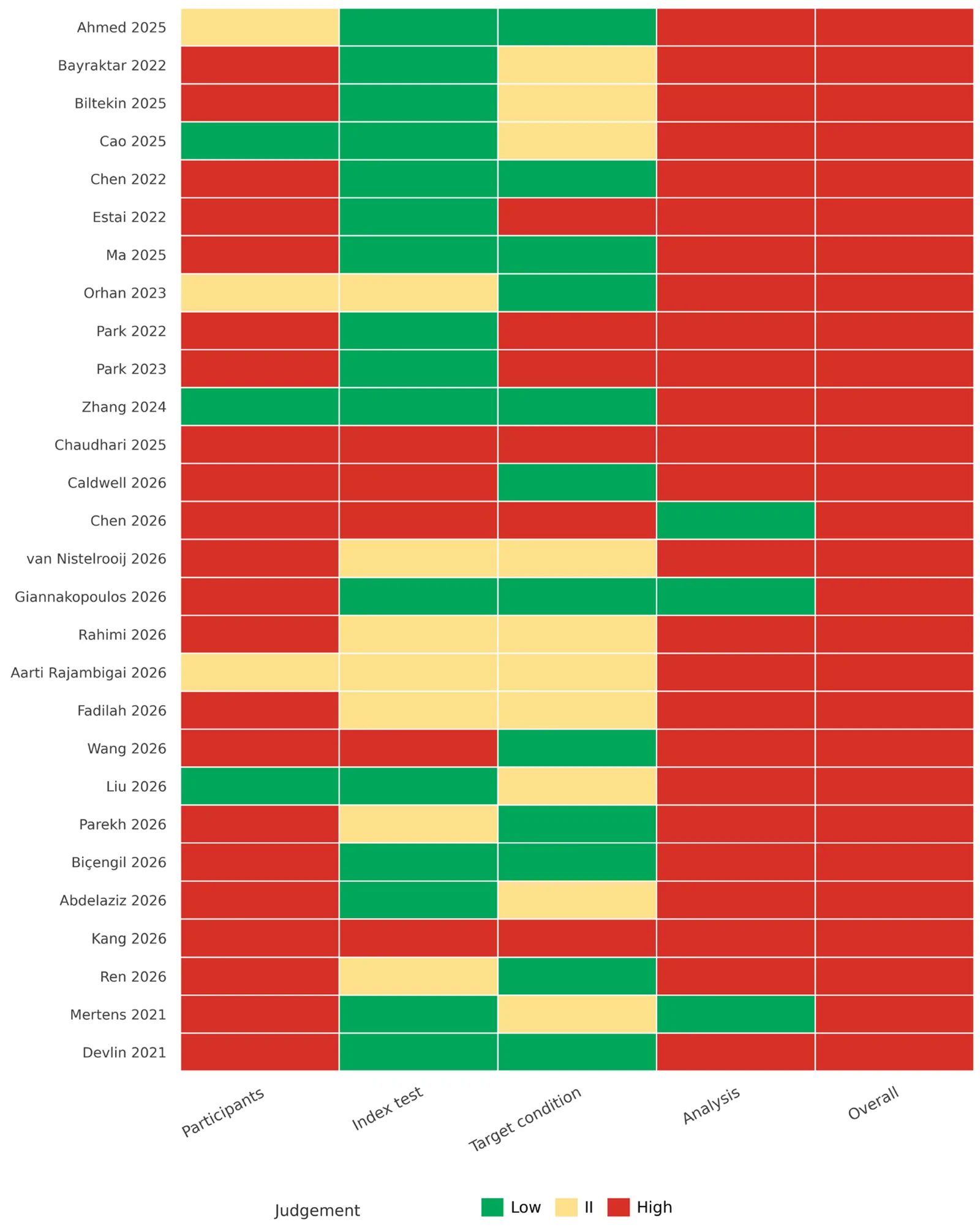
QUADAS-3 v1.2 estimate-level risk-of-bias judgments for 28 distinct estimates [4,14,15,16,17,18,20,21,22,23,24,25,26,27,28,29,30,31,32,33,34,35,36,37,38,39,40,41]. Green indicates low risk, yellow indicates insufficient information (II), and red indicates high risk. Overall risk of bias was high for all estimates.

QUADAS-3 was applied to 28 distinct numerical estimates (Figure 2). Arsiwala-Scheppach et al. [19] was an ancillary report of the Mertens et al. [33] trial and supplied no separate diagnostic-accuracy estimate. Final consensus risk of bias was high for all 28 estimates. The analysis domain was high risk in 25 (89.3%), primarily because clustered teeth, surfaces, images, or patches were analyzed as independent and because exclusions, missing observations, or indeterminate results were incompletely handled.

Participants were high risk in 22 estimates (78.6%); index test was high risk in 5 (17.9%); and target condition was high risk in 6 (21.4%). Overall applicability concern was high in 17 estimates (60.7%), low in 8 (28.6%), and based on insufficient information in 3 (10.7%). Detailed judgments are in Supplementary Files S2 and S4.

### 3.3. Clinical Composition of the Evidence

The 29 reports represented 28 studies published from 2021 to 2026 across 16 countries or country groups. Imaging modality, target condition, observational unit, and reference standard varied substantially. Only five studies used an independent external dataset. Table 1 makes the principal composition and evidence constraints visible in the main text; full report-level characteristics are provided in Supplementary File S3.

### 3.4. Study-Level Standalone-AI Accuracy

Twelve standalone-AI reports supplied exact, internally coherent contingency cells for a clinically interpretable unit. The subset comprised four bitewing, two panoramic, three photographic or QLF, one CBCT, one 3D intraoral scan, and one mixed-radiograph study. Five estimates used surfaces, five used teeth, and two used images; only one used external validation.

**Figure 3:**
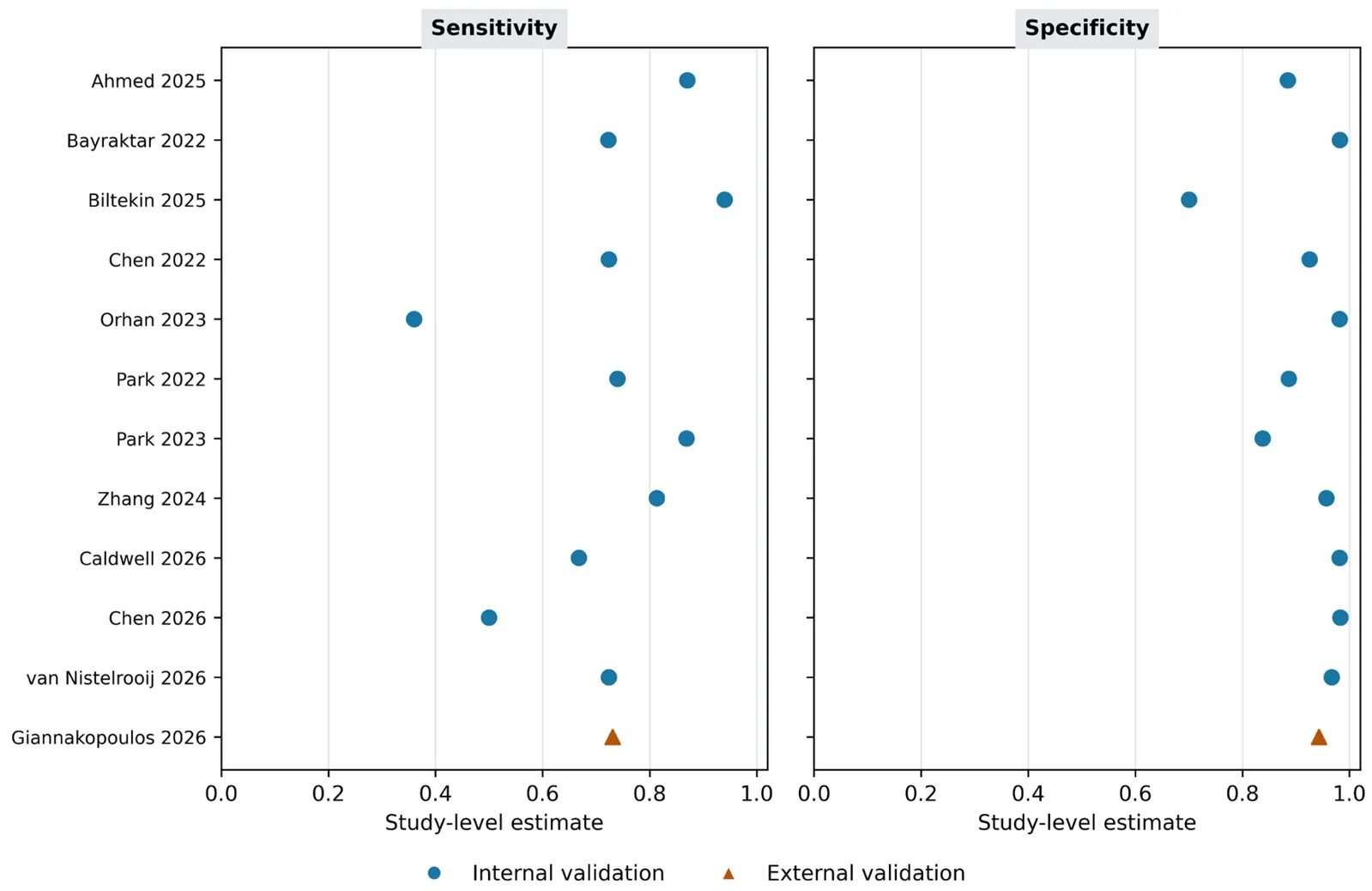
Study-level sensitivity and specificity for the 12 standalone-AI reports [18,20,22,23,25,26,29,34,36,37,39,41] with exact, coherent 2 × 2 data. Circles indicate internal validation and triangles external validation. No pooled estimate or independence-based confidence interval is shown.

Sensitivity ranged from 0.360 to 0.940 and specificity from 0.700 to 0.983 (Figure 3). These values describe an availability subset selected partly by reporting completeness. No pooled operating point was calculated. The main figure omits binomial confidence intervals because the required participant-level clustering information was generally unavailable and independent-observation intervals could be anti-conservative.

Certainty for standalone-AI sensitivity and specificity was very low because of very serious risk of bias and indirectness, serious inconsistency and imprecision, and concern about missing evidence (Supplementary Table S4).

### 3.5. Clinician Interpretation with AI Assistance

Devlin et al. [27] randomized 23 dentists to read the same 24 bitewings without or with AssistDent prompts. Sensitivity was 0.443 without AI and 0.758 with AI, while specificity was 0.963 and 0.854, respectively; both differences were statistically significant. Mertens et al. [33] used a cluster-randomized crossover design. Mean sensitivity increased from 0.72 without AI to 0.81 with AI and AUC increased from 0.85 to 0.89; specificity did not differ significantly, but AI assistance increased invasive treatment decisions.

The two studies therefore suggest that AI can increase reader sensitivity, but they do not establish net clinical benefit. Their designs, case allocation, outcomes, and tradeoffs differed, and neither evaluated patient outcomes in routine care. Certainty for incremental clinician benefit was very low.

## 4. Discussion

This review found a broad and methodologically fragile evidence base. All 28 assessed estimates had high overall risk of bias, only 5 studies used external validation, and the 12 reports with exact contingency data spanned sensitivity from 0.360 to 0.940 and specificity from 0.700 to 0.983. These reports did not estimate one clinically exchangeable quantity, so a cross-modality pooled operating point was not retained.

Controlled reader evidence is more informative for human–AI interaction than standalone algorithm accuracy. Devlin and Mertens both reported sensitivity gains with AI, but one showed lower specificity and the other more invasive treatment decisions. Improved detection is therefore not equivalent to improved care.

### 4.1. Comparison with Previous Evidence

The numerical estimates reported by previous meta-analyses depend strongly on scope. Abbott et al. [1] pooled seven mixed clinical-image and radiographic studies and reported sensitivity of 0.76 and specificity of 0.91. Ammar and Kuhnisch [3] restricted the question to bitewing caries detection and reported sensitivity of 0.87 and specificity of 0.89 across five studies. Carvalho et al. [42], focusing on approximal caries on bitewings, reported pooled sensitivity and specificity of approximately 0.94 and 0.91. These summary values fall within the wide study-level ranges observed here, but direct comparison is inappropriate because the included modalities, lesion thresholds, units, and contingency-data rules differ.

The stricter exact-data rule reduced the quantitative denominator but did not create a homogeneous clinical question. Its contribution is to expose how much published evidence cannot support verifiable contingency analysis and how strongly study composition governs any apparent average.

### 4.2. Sources of Dispersion and Uncertainty

Target conditions ranged from enamel-only lesions to cavitated or any caries; observational units ranged from surfaces and teeth to images and patches; and reference standards ranged from single-examiner image labels to multi-expert consensus or clinical examination. These differences can change both sensitivity and specificity and were not tested as causal moderators.

Clustering is a particularly important limitation. Many reports treated multiple teeth, surfaces, lesions, or images from the same participant as independent observations. Without participant-level data or cluster-adjusted variances, conventional binomial intervals and hierarchical meta-analysis can overstate precision. Retrospective exclusion of technically difficult images and internal testing from the same centers or devices further limits transportability.

### 4.3. Clinical Utility and Human–AI Evaluation

The reader studies indicate that AI prompts can change diagnostic behavior, not merely reproduce standalone algorithm performance. Higher sensitivity may be clinically valuable for early lesions, but false-positive prompts and more invasive management can offset that benefit. Paired or crossover evaluations should account for repeated readers, repeated cases, treatment thresholds, and downstream decisions.

Cost-effectiveness was outside this review’s diagnostic-accuracy eligibility criteria. Schwendicke et al. [43], a linked economic evaluation excluded from the systematic review, reported similar modeled tooth retention and costs with and without AI and substantial uncertainty. We therefore make no within-review claim about cost-effectiveness.

Future evaluations should use locked models, prespecified thresholds, independent multicenter and multivendor data, clinically meaningful lesion definitions, and participant-level analyses. Reader studies should randomize or counterbalance reading order, include washout where appropriate, and report diagnostic errors, confidence, management decisions, reading time, workload, equity, unintended consequences, and patient outcomes. STARD-AI, DECIDE-AI, and CONSORT-AI provide relevant reporting frameworks [44,45,46].

### 4.4. Strengths and Limitations

Strengths include explicit separation of standalone and clinician-plus-AI constructs, transparent estimate-selection rules, exact contingency checks, study-level presentation, unmodified QUADAS-3, and structured certainty assessment.

The largest limitation is the selection audit. Rule-based preprocessing removed 130,245 of 137,274 records before duplicate human screening. Because the excluded record set, rule-level counts, and validation sample were not retained, the false-negative rate cannot be estimated. The screening export also does not preserve independent pre-consensus decisions. These deficiencies materially reduce auditability and contributed to very-low certainty.

Coverage was further limited by omission of IEEE Xplore, registers, preprints, organizational websites, and gray literature. Authors were not contacted, so the exact-data subset is an availability sample influenced by reporting completeness. The QUADAS-3 process involved independent source verification of a shared pre-populated matrix rather than de novo duplicate coding, and no reliability statistic is claimed.

All assessed estimates were at high overall risk of bias, and 25 had high analysis-domain risk. Cluster-adjusted uncertainty was generally unavailable; external validation was sparse; and the small, clinically diverse evidence base did not support meta-regression, formal reporting-bias tests, or a pooled operating point.

## 5. Conclusions

Published accuracy estimates for standalone AI caries detection vary widely across non-exchangeable modalities, lesion thresholds, observational units, reference standards, and validation designs. With high risk of bias in all assessed estimates and very-low certainty, the evidence does not support a transferable clinical performance benchmark.

Two controlled reader studies suggest that AI assistance can increase sensitivity, but specificity and treatment-intensity tradeoffs remain and net clinical benefit has not been established. Future research should prioritize locked-model external validation and controlled prospective human–AI studies that measure treatment decisions, workflow, and patient-relevant outcomes.

## Figures and Tables

**Table 1:** Clinical composition and evidential constraints of the 28 unique studies.

Characteristic	Evidence Profile
Reports/unique studies	29 reports/28 studies
Publication period	2021–2026
Imaging modalities	Bitewing 9; intraoral photography 6; panoramic 5; mixed 3; CBCT 2; 3D intraoral scan 1; periapical 1; QLF 1
Evaluated index-test construct	Standalone algorithm output: 26 studies; clinician-plus-AI interpretation: 2 studies
Target conditions	Enamel-only, any proximal/approximal, cavitated, tooth-level caries presence, lesion segmentation, and multiclass severity tasks
Observational units	Patient, image, reader–image pair, tooth, surface, lesion, region of interest, and computational patch
Independent external validation	5/28 studies (17.9%)
Exact standalone-AI 2 × 2 data	12/28 studies (42.9%); descriptive availability subset, not a pooled clinical sample
Controlled clinician-plus-AI studies	2 studies [27,33]
Overall QUADAS-3 risk of bias	High in 28/28 estimates (100%)

## Data Availability

All data generated or analyzed during this study are available in Zenodo at https://zenodo.org/records/22286374, accessed on 28 August 2026, with reference https://doi.org/10.5281/zenodo.22286374.

## Supplementary Materials

The following supporting information can be downloaded at: https://www.mdpi.com/article/10.3390/diagnostics16182995/s1. Supplementary File S1: Final Database Search Strategies and Rayyan-Assisted Preprocessing; Supplementary File S2: Revised Complementary Tables and Figures; Supplementary File S3: Study Characteristics, Estimate Selection, and Analytic Disposition; Supplementary File S4: Complete QUADAS-3 v1.2 Assessment; PRISMA_2020_Checklist_Completed. References [4,11,12,13,14,15,16,17,18,19,20,21,22,23,24,25,26,27,28,29,30,31,32,33,34,35,36,37,38,39,40,41] are cited in the Supplementary Materials and are included in the main reference list.
