# Supplementary material for: Diagnostic Accuracy of Artificial Intelligence for Dental Caries Detection Across Clinical Imaging Modalities: A Systematic Review and Descriptive Synthesis
Source: Diagnostics (Basel). 2026 Sep 16;16(18):2995. doi: 10.3390/diagnostics16182995 (PMC13605960; doi:10.3390/diagnostics16182995)
Supplement: Supplementary file 1 [file diagnostics-16-02995-s001.zip › Supplementary_File_S1_Revised_Search_and_Rayyan_Workflow.pdf]

Supplementary File S1. Final Database Search Strategies and Rayyan-Assisted Preprocessing

Diagnostic Accuracy of Artificial Intelligence for Dental Caries Detection across Clinical Imaging Modalities: A Systematic Review and Descriptive Synthesis

Final status. The five database searches were rerun without publication-type, study-design, subject-area, database-category, or disease-mapping restrictions. All searches were completed on 10 June 2026. The strategies, database-specific raw yields, Rayyan-assisted preprocessing, safeguards, and reconciled PRISMA arithmetic below constitute the definitive search record used by the manuscript, Figure 1, data files, analysis script, and repository.

S1.1. Information sources and search execution

Database	Platform/interface	Fields and vocabulary	Run date	Coverage/date control	Raw yield	Database-level restrictions
PubMed	PubMed, U.S. National Library of Medicine	MeSH plus free-text terms	10 Jun 2026	1900-01-01 to 2026-06-10	31,663	None by publication type or study design
Scopus	Scopus, Elsevier	TITLE-ABS-KEY	10 Jun 2026	Records loaded before 11 Jun 2026	37,582	No Dentistry-only subject restriction
Web of Science	Web of Science Core Collection, Clarivate	Topic field (TS)	10 Jun 2026	1900-01-01 to 2026-06-10	13,836	No Dentistry/Oral Surgery category restriction
Embase	Embase.com, Elsevier	Emtree plus free-text terms	10 Jun 2026	1947-01-01 to 2026-06-10	53,523	No disease-mapping restriction
Cochrane Library	Cochrane Library, Wiley	Title, abstract, keywords	10 Jun 2026	Coverage available through run date	670	No Trials-only record-type restriction

Total raw yield: 137,274 records.

S1.2. Exact executed strategies

PubMed (raw yield: 31,663)

```
( "Artificial Intelligence"[Mesh] OR "Machine Learning"[Mesh] OR "Deep Learning"[Mesh] OR "Neural Networks, Computer"[Mesh] OR "artificial intelligence" OR "machine learning" OR "deep learning" OR "neural network*" OR "convolutional neural network*" OR CNN OR "transformer*" OR "radiomics" OR "computer vision" OR "image recognition" OR "pattern recognition" OR "automated analysis" OR "algorithm*" OR "supervised learning" OR "unsupervised learning" OR "reinforcement learning" ) AND ( ("Diagnosis"[Mesh] OR "Diagnostic Imaging"[Mesh] OR diagnos* OR detect* OR "screening" OR "classification" OR "computer-aided diagnosis" OR "computer-assisted diagnosis" OR "decision support" OR "clinical decision support" OR "lesion detection" OR "disease identification") AND ("Sensitivity and Specificity"[Mesh] OR "Reproducibility of Results"[Mesh] OR "Predictive Value of Tests"[Mesh] OR accuracy OR precision OR "diagnostic accuracy" OR "diagnostic performance" OR "diagnostic validity" OR "predictive value" OR "ROC curve" OR "AUC" OR "area under the curve" OR "diagnostic odds ratio" OR "likelihood ratio" OR "validation" OR "model performance" OR "calibration" OR "discrimination" OR "true positive" OR "false negative") ) AND ( "Dentistry"[Mesh] OR "Dental Clinics"[Mesh] OR "Oral Medicine"[Mesh] OR "Diagnostic Imaging, Dental"[Mesh] OR dentistry OR dental OR oral OR odontolog* OR stomatology OR maxillofacial OR tooth OR teeth OR periodontal OR caries OR decay OR endodontic OR periapical OR implant OR prosthodontic OR orthodontic OR radiograph* OR "bite-wing" OR "periapical radiograph*" OR "panoramic radiograph*" OR CBCT OR "cone beam computed tomography" OR "intraoral imaging" OR "3D imaging" OR "oral mucosa" OR "oral cancer" OR "oral lesion" OR "oral pathology" ) AND ("1900/01/01"[Date - Publication] : "2026/06/10"[Date - Publication])
```

Scopus (raw yield: 37,582)

```
TITLE-ABS-KEY( ( "artificial intelligence" OR "machine learning" OR "deep learning" OR "neural network*" OR "convolutional neural network*" OR CNN OR "transformer*" OR "radiomics" OR "computer vision" OR "image recognition" OR "pattern recognition" OR "automated analysis" OR "algorithm*" OR "supervised learning" OR "unsupervised learning" OR "reinforcement learning" ) AND ( (diagnos* OR detect* OR "screening" OR "classification" OR "computer-aided diagnosis" OR "computer-assisted diagnosis" OR "decision support" OR "clinical decision support" OR "lesion detection" OR "disease identification") AND (accuracy OR precision OR "sensitivity" OR "specificity" OR "diagnostic accuracy" OR "diagnostic performance" OR "predictive value" OR "ROC curve" OR "AUC" OR "area under the curve" OR "diagnostic odds ratio" OR "likelihood ratio" OR validation OR "model performance" OR "calibration" OR "discrimination" OR "true positive" OR "false negative") ) AND ( dentistry OR dental OR oral OR odontolog* OR stomatology OR maxillofacial OR tooth OR teeth OR periodontal OR caries OR decay OR endodontic OR periapical OR implant OR prosthodontic OR orthodontic OR radiograph* OR "bite-wing" OR "periapical radiograph*" OR "panoramic radiograph*" OR CBCT OR "cone beam computed tomography" OR "intraoral imaging" OR "3D imaging" OR "oral mucosa" OR "oral cancer" OR "oral lesion" OR "oral pathology" ) ) AND ORIG-LOAD-DATE BEF 1781136000
```

Web of Science Core Collection (raw yield: 13,836)

```
TS=( ( "artificial intelligence" OR "machine learning" OR "deep learning" OR "neural network*" OR "convolutional neural network*" OR CNN OR "transformer*" OR "radiomics" OR "computer vision" OR "image recognition" OR "pattern recognition" OR "automated analysis" OR "algorithm*" OR "supervised learning" OR "unsupervised learning" OR "reinforcement learning" ) AND ( (diagnos* OR detect* OR "screening" OR "classification" OR "computer-aided diagnosis" OR "computer-assisted diagnosis" OR "decision support" OR "clinical decision support" OR "lesion detection" OR "disease identification") AND (accuracy OR precision OR "sensitivity" OR "specificity" OR "diagnostic accuracy" OR "diagnostic performance" OR "predictive value" OR "ROC curve" OR "AUC" OR "area under the curve" OR "diagnostic odds ratio" OR "likelihood ratio" OR validation OR "model performance" OR "calibration" OR "discrimination" OR "true positive" OR "false negative") ) AND ( dentistry OR dental OR oral OR odontolog* OR stomatology OR maxillofacial OR tooth OR teeth OR periodontal OR caries OR decay OR endodontic OR periapical OR implant OR prosthodontic OR orthodontic OR radiograph* OR "bite-wing" OR "periapical radiograph*" OR "panoramic radiograph*" OR CBCT OR "cone beam computed tomography" OR "intraoral imaging" OR "3D imaging" OR "oral mucosa" OR "oral cancer" OR "oral lesion" OR "oral pathology" ) ) AND DOP=1900-01-01/2026-06-10
```

Embase (raw yield: 53,523)

```
('artificial intelligence'/exp OR 'machine learning'/exp OR 'deep learning'/exp OR 'artificial neural network'/exp OR 'artificial intelligence' OR 'machine learning' OR 'deep learning' OR 'neural network*' OR 'convolutional neural network*' OR 'cnn' OR 'transformer*' OR 'radiomics' OR 'computer vision' OR 'image recognition' OR 'pattern recognition' OR 'automated analysis' OR 'algorithm*' OR 'supervised learning' OR 'unsupervised learning' OR 'reinforcement learning') AND (('diagnosis'/exp OR 'diagnostic imaging'/exp OR 'diagnos*' OR 'detect*' OR 'screening' OR 'classification' OR 'computer-aided diagnosis' OR 'computer-assisted diagnosis' OR 'decision support' OR 'clinical decision support' OR 'lesion detection' OR 'disease identification') AND ('sensitivity and specificity'/exp OR 'reproducibility'/exp OR 'predictive value'/exp OR 'accuracy' OR 'precision' OR 'diagnostic accuracy' OR 'diagnostic performance' OR 'diagnostic validity' OR 'predictive value' OR 'roc curve' OR 'auc' OR 'area under the curve' OR 'diagnostic odds ratio' OR 'likelihood ratio' OR 'validation' OR 'model performance' OR 'calibration' OR 'discrimination' OR 'true positive' OR 'false negative')) AND ('dentistry'/exp OR 'dental clinic'/exp OR 'stomatology'/exp OR 'diagnostic imaging, dental'/exp OR 'dentistry' OR 'dental' OR 'oral' OR 'odontolog*' OR 'stomatology' OR 'maxillofacial' OR 'tooth' OR 'teeth' OR 'periodontal' OR 'caries' OR 'decay' OR 'endodontic' OR 'periapical' OR 'implant' OR 'prosthodontic' OR 'orthodontic' OR 'radiograph*' OR 'bite-wing' OR 'periapical radiograph*' OR 'panoramic radiograph*' OR 'cbct' OR 'cone beam computed tomography' OR 'intraoral imaging' OR '3d imaging' OR 'oral mucosa' OR 'oral cancer' OR 'oral lesion' OR 'oral pathology') AND [1947-01-01 to 2026-06-10]/ld
```

Cochrane Library (raw yield: 670)

```
( ("artificial intelligence" OR "machine learning" OR "deep learning" OR "neural network*" OR "convolutional neural network*" OR CNN OR "transformer*" OR "radiomics" OR "computer vision" OR "image recognition" OR "pattern recognition" OR "automated analysis" OR "algorithm*" OR "supervised learning" OR "unsupervised learning" OR "reinforcement learning"):ti,ab,kw ) AND ( ((diagnos* OR detect* OR "screening" OR "classification" OR "computer-aided diagnosis" OR "computer-assisted diagnosis" OR "decision support" OR "clinical decision support" OR "lesion detection" OR "disease identification") AND (accuracy OR precision OR "sensitivity" OR "specificity" OR "diagnostic accuracy" OR "diagnostic performance" OR "predictive value" OR "ROC curve" OR "AUC" OR "area under the curve" OR "diagnostic odds ratio" OR "likelihood ratio" OR validation OR "model performance" OR "calibration" OR "discrimination" OR "true positive" OR "false negative")):ti,ab,kw ) AND ( (dentistry OR dental OR oral OR odontolog* OR stomatology OR maxillofacial OR tooth OR teeth OR periodontal OR caries OR decay OR endodontic OR periapical OR implant OR prosthodontic OR orthodontic OR radiograph* OR "bite-wing" OR "periapical radiograph*" OR "panoramic radiograph*" OR CBCT OR "cone beam computed tomography" OR "intraoral imaging" OR "3D imaging" OR "oral mucosa" OR "oral cancer" OR "oral lesion" OR "oral pathology"):ti,ab,kw )
```

S1.3. Rayyan-assisted rule-based preprocessing

Purpose and timing. The database searches were intentionally sensitive and broad. After all database exports were imported into Rayyan and before manual title/abstract screening, a deterministic preprocessing step was used to remove records that were unequivocally incompatible with the eligibility criteria.

Functions used. Rayyan's filterable bibliographic fields, record-type facets, keyword searches, labels, exclusion reasons, multi-select, and bulk actions were used. The procedure was configured and initiated by the review team; it was not an autonomous study-selection model.

Rule families. Rules targeted clearly non-primary or secondary publication types (for example, reviews and meta-analyses, editorials, commentaries, letters, protocols, book material, and other non-original record types) and records whose indexed fields or title/abstract content placed them outside dental-caries diagnostic evaluation. No rule was based solely on a Rayyan relevance score or generative-AI recommendation.

Safeguards and unresolved audit limitation. Each rule family was checked against the eligibility criteria before bulk action, and ambiguous records were intended to be retained. However, the retained audit trail does not contain the complete bulk-excluded record set, rule-specific counts, or a human-screened validation sample. The false-negative rate for the 130,245 rule-excluded records therefore cannot be estimated, and these records did not undergo duplicate human screening.

Result. The Rayyan-assisted procedure marked 130,245 records ineligible before manual screening. A further 3,781 duplicates were removed, leaving 3,248 records for independent title/abstract screening. The magnitude and unaudited false-negative performance of the bulk exclusion are major review-level limitations.

S1.4. Human screening and agreement reporting

Human eligibility decisions. LPV and MSV independently screened the 3,248 retained titles/abstracts and subsequently assessed the 35 retrieved full texts. Disagreements were resolved by discussion, with JM available for adjudication. The automated preprocessing did not replace these eligibility decisions.

Agreement statistic and auditability. The export preserving both reviewers' original blinded pre-consensus decisions was not retained. Neither Cohen's kappa nor percentage agreement can be reconstructed. The final consensus set is documented, but the reviewer-level pre-consensus decisions cannot be independently audited.

S1.5. Reconciled PRISMA accounting

Stage	Count	Arithmetic/check
Raw records identified	137,274	31,663 + 37,582 + 13,836 + 53,523 + 670
Marked ineligible by Rayyan-assisted automation	130,245	Deterministic rules and bulk actions
Duplicate records removed	3,781	Rayyan duplicate management
Records screened	3,248	137,274 - 130,245 - 3,781
Records excluded at title/abstract stage	3,213	Independent human screening
Full-text reports sought/retrieved/assessed	35	3,248 - 3,213
Full-text reports excluded	6	Irrelevant outcomes, n=2; unsuitable designs, n=4
Reports included	29	35 - 6
Unique studies	28	One ancillary report linked to Mertens 2021
Standalone-AI exact 2 x 2 descriptive subset	12	Exact, coherent, clinically interpretable 2 x 2 data; no pooling
Clinician-plus-AI estimate	1	Reported separately

S1.6. Protocol deviation and scope limitations

IEEE Xplore. IEEE Xplore was listed as a planned information source but was not searched because the required institutional interface/API access was unavailable. This is reported as a protocol deviation in the manuscript and protocol-amendment file.

Other sources. Study registers, preprint servers, organizational websites, and other grey-literature sources were not searched. Study authors were not contacted to reconstruct unavailable or internally inconsistent 2 x 2 cells.

Export audit limitation. Original export filenames and checksums, the complete bulk-excluded record set, rule-specific exclusion counts, a validation sample, and blinded pre-consensus reviewer decisions were not retained consistently. Consequently, the automation false-negative rate and inter-reviewer agreement cannot be reconstructed.

S1.7. Software citation

Ouzzani M, Hammady H, Fedorowicz Z, Elmagarmid A. Rayyan-a web and mobile app for systematic reviews. Systematic Reviews. 2016;5:210. doi:10.1186/s13643-016-0384-4.
