# Supplementary material for: Diagnostic Accuracy of Artificial Intelligence for Dental Caries Detection Across Clinical Imaging Modalities: A Systematic Review and Descriptive Synthesis
Source: Diagnostics (Basel). 2026 Sep 16;16(18):2995. doi: 10.3390/diagnostics16182995 (PMC13605960; doi:10.3390/diagnostics16182995)
Supplement: Supplementary file 1 [file diagnostics-16-02995-s001.zip › Supplementary_File_S2_Revised_Tables_and_Figures.pdf]

Supplementary File S2. Revised Complementary Tables and Figures

Diagnostic Accuracy of Artificial Intelligence for Dental Caries Detection across Clinical Imaging Modalities: A Systematic Review and Descriptive Synthesis

The primary paper presents descriptive study-level accuracy and controlled clinician-plus-AI evidence. No cross-modality pooled operating point, HSROC, prediction region, likelihood ratio, diagnostic odds ratio, or predictive-value scenario is included.

Table S1. QUADAS-3 risk-of-bias and applicability summary

Assessment	Domain	Judgement	n	%
Risk of bias	Participants	Low	3	10.7
Risk of bias	Participants	II	3	10.7
Risk of bias	Participants	High	22	78.6
Risk of bias	Index test	Low	16	57.1
Risk of bias	Index test	II	7	25.0
Risk of bias	Index test	High	5	17.9
Risk of bias	Target condition	Low	12	42.9
Risk of bias	Target condition	II	10	35.7
Risk of bias	Target condition	High	6	21.4
Risk of bias	Analysis	Low	3	10.7
Risk of bias	Analysis	II	0	0.0
Risk of bias	Analysis	High	25	89.3
Risk of bias	Overall	Low	0	0.0
Risk of bias	Overall	II	0	0.0
Risk of bias	Overall	High	28	100.0
Applicability	Participants	Low	12	42.9
Applicability	Participants	II	1	3.6
Applicability	Participants	High	15	53.6
Applicability	Index test	Low	20	71.4
Applicability	Index test	II	0	0.0
Applicability	Index test	High	8	28.6
Applicability	Target condition	Low	18	64.3
Applicability	Target condition	II	8	28.6
Applicability	Target condition	High	2	7.1
Applicability	Overall	Low	8	28.6
Applicability	Overall	II	3	10.7
Applicability	Overall	High	17	60.7

Figure S1. Study-level estimates by observational unit

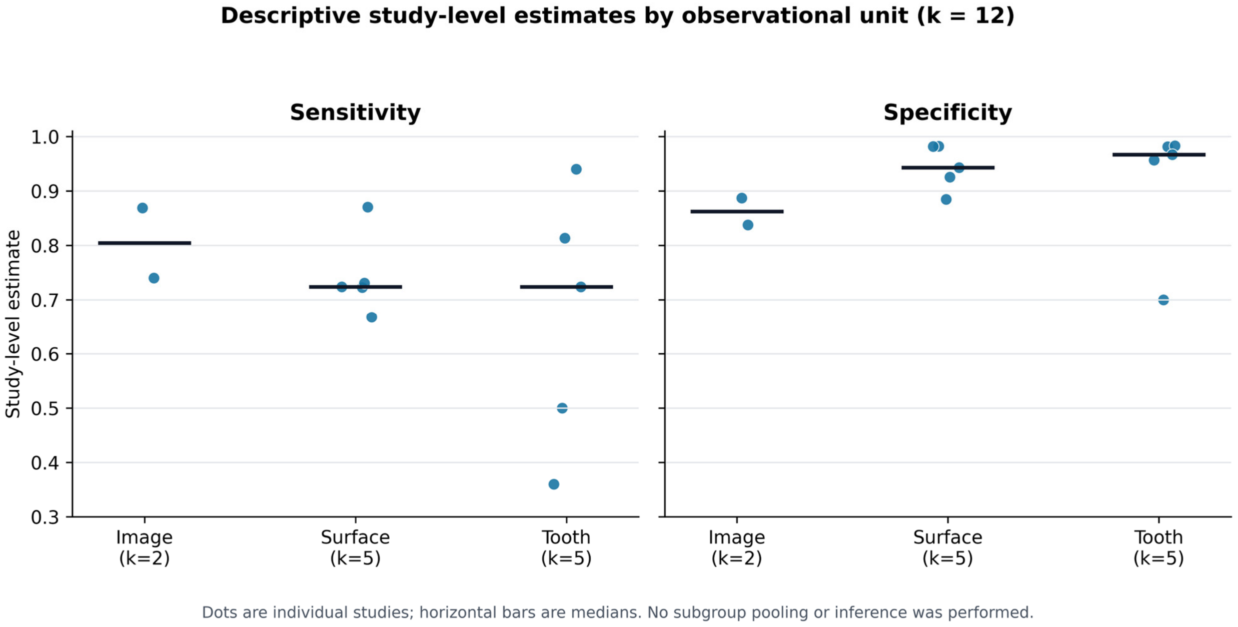

Dots are individual exact-data estimates and crossbars are medians. The k = 12 set includes image (k = 2), surface (k = 5), and tooth (k = 5) observations. No subgroup pooling or inferential comparison was performed.

Table S2. Descriptive ranges by observational unit

Unit	k	Sens. min	Sens. median	Sens. max	Spec. min	Spec. median	Spec. max
Image	2	0.740	0.804	0.869	0.837	0.862	0.887
Surface	5	0.668	0.724	0.870	0.885	0.943	0.982
Tooth	5	0.360	0.724	0.940	0.700	0.967	0.983

Table S3. Controlled clinician interpretation without and with AI

Study	Condition	Design	Sensitivity	Specificity	AUC	Interpretation
Devlin 2021	Without AI	Randomized parallel reader groups	0.443	0.963	NR	Lower sensitivity and fewer false-positive prompts
Devlin 2021	With AI	Randomized parallel reader groups	0.758	0.854	NR	Higher sensitivity with lower specificity; both differences p < 0.01
Mertens 2021	Without AI	Cluster-randomized crossover reader study	0.72	NR (no significant between-condition difference)	0.85	Lower sensitivity and AUC
Mertens 2021	With AI	Cluster-randomized crossover reader study	0.81	NR (no significant between-condition difference)	0.89	Higher sensitivity and AUC; invasive treatment decisions increased

Table S4. GRADE-DTA certainty assessment

Outcome	RoB	Inconsistency	Indirectness	Imprecision	Publication bias	Overall	Basis

Outcome	RoB	Inconsistency	Indirectness	Imprecision	Publication bias	Overall	Basis
Standalone-AI study-level sensitivity and specificity	Very serious	Serious	Very serious	Serious	Serious concern	Very low	28/28 estimates had high overall QUADAS-3 risk and 25/28 had high Analysis risk. Thresholds, units, reference standards, validation, and clustering differed; search coverage and bulk-exclusion auditability were incomplete.
Incremental effect of AI assistance on clinician performance	Serious	Serious	Serious	Serious	Undetected; evidence sparse	Very low	Only two controlled reader studies were available. Both concerned proximal caries in simulated reading settings; specificity and treatment effects differed, and patient outcomes were not evaluated.
