# Supplementary material for: Diagnostic Accuracy of Artificial Intelligence for Dental Caries Detection Across Clinical Imaging Modalities: A Systematic Review and Descriptive Synthesis
Source: Diagnostics (Basel). 2026 Sep 16;16(18):2995. doi: 10.3390/diagnostics16182995 (PMC13605960; doi:10.3390/diagnostics16182995)
Supplement: Supplementary file 1 [file diagnostics-16-02995-s001.zip › Supplementary_File_S3_Revised_Study_Overview.pdf]

Supplementary File S3. Study Characteristics, Estimate Selection, and Analytic Disposition

The review includes 29 reports representing 28 unique studies. Twelve standalone algorithm-output reports contain exact, internally coherent 2 x 2 data and form a study-level descriptive subset; controlled clinician-plus-AI evidence is summarized separately; the remaining reports are retained using reported metrics and narrative characteristics. No cross-modality meta-analysis is performed.

Part A. Study characteristics and diagnostic context

Report	Year	Country	Design	Imaging modality	Unit	Evaluated index-test construct	Intended-use context	Target-condition threshold	External validation
Ahmed 2025	2025	Saudi Arabia	Prospective clinical trial (diagnostic performance evaluation)	3D intraoral scans (Medit i700)	Tooth surface (3D point-cloud units from IOS)	Standalone algorithm output	Research/development evaluation of automated 3D-scan output; clinical deployment role not prospectively evaluated.	Study-defined caries presence using modified ICDAS.	No
Arsiwala-Scheppach 2024	2024	Germany	Randomized, controlled, non-blinded, clustered cross-over clinical trial	Bitewing radiographs (digital RVG)	Dentist–image pair (gaze dataset); regions of interest (teeth, carious lesions, restorations)	AI-assisted clinician interpretation	Clinician-facing second-reader/decision-support overlay evaluated within a reader trial.	Proximal caries across enamel/dentin depth categories; gaze report provided no distinct DTA estimate.	No
Bayraktar 2022	2022	Türkiye	Retrospective diagnostic accuracy study (CNN development + internal testing)	Digital bitewing radiographs	Approximal tooth surfaces (premolars and molars)	Standalone algorithm output	Research/development evaluation of automated lesion detection; no deployed workflow evaluated.	Proximal caries presence on approximal surfaces.	No
Biltekin 2025	2025	Türkiye	Retrospective diagnostic accuracy study (DL model development and internal testing)	Panoramic radiography (extraoral)	Individual teeth (cropped tooth images)	Standalone algorithm output	Research/development benchmark of automated classification; no deployed workflow evaluated.	Carious versus sound cropped tooth image.	No
Cao 2025	2025	Germany/The Netherlands/Slovakia	Retrospective multi-center AI development + external validation study (instance segmentation and multi-class classification)	Digital bitewing radiographs	– Teeth (instance segmentation) – Per-tooth multi-class semantic segmentation (seven conditions)	Standalone algorithm output	Automated chart-filing and diagnostic-support pipeline intended for clinician review.	Study-defined caries finding within a seven-condition per-tooth classification task.	Yes
Chen 2022	2022	China	Retrospective diagnostic accuracy study involving development and testing of a Faster R-CNN object detection model for identifying proximal caries lesions on bitewing radiographs. The model's performance was compared against postgraduate dental students.	Digital bitewing radiographs.	Individual proximal surfaces on posterior teeth (mesial and distal surfaces assessed independently).	Standalone algorithm output	Automated detector evaluated against a reference standard; potential clinician support was not the evaluated index test.	Any proximal caries across E1-E2 and D1-D3 depth categories.	No
Devlin 2021	2021	United Kingdom	Randomized controlled comparative diagnostic accuracy study evaluating dentists' ability to detect enamel-only proximal caries on bitewing radiographs with vs. without assistance from the AssistDent artificial intelligence software.	Digital bitewing radiographs from diverse clinical sources (hospital + 9 GDP clinics). No specific exposure parameters were reported → NR	Individual proximal tooth surfaces.	AI-assisted clinician interpretation	Clinician-facing second-reader prompts (AssistDent) evaluated as clinician plus AI.	Enamel-only proximal caries.	No

Part A. Study characteristics and diagnostic context (continued)

Report	Year	Country	Design	Imaging modality	Unit	Evaluated index-test construct	Intended-use context	Target-condition threshold	External validation
Estai 2022	2022	Australia	Retrospective diagnostic accuracy study evaluating a two-stage deep learning system (ROI detection via Faster R-CNN + caries classification via Inception-ResNet-v2) for automatic detection of proximal caries on digital bitewing radiographs, compared against expert-generated reference standard.	Digital bitewing radiographs acquired using photostimulable phosphor (PSP) plate technique.	Region of interest (ROI) corresponding to proximal surface.	Standalone algorithm output	Research/development evaluation of automated proximal-caries detection.	Study-defined proximal caries according to WHO/ICDAS/Mejare criteria.	No
Ma 2025	2025	China	Retrospective AI-development and internal validation study evaluating performance of Mask R-CNN for detecting and segmenting teeth and dental caries on cone-beam computed tomography (CBCT) images. Three hyperparameter groups were compared: R group – no transfer learning, random initialization I group – transfer learning from ImageNet C group – transfer learning from COCO	Cone-beam computed tomography (CBCT).	Entire tooth volume / tooth-level and pixel-level regions of interest (segmentations). Each CBCT image contains multiple teeth and possibly carious regions.	Standalone algorithm output	Research/development evaluation of automated CBCT segmentation.	CBCT caries region segmentation.	No
Mertens 2021	2021	Germany	Cluster-randomized cross-over controlled trial evaluating the effect of an AI-based decision-support system (dentalXrai Pro) on proximal caries detection by dentists using bitewing radiographs.	Digital bitewing radiographs	Proximal tooth surface.	AI-assisted clinician interpretation	Clinician-facing decision-support overlay evaluated as clinician plus AI.	Proximal caries across enamel and dentin lesion-depth categories.	No
Orhan 2023	2023	Türkiye	Retrospective diagnostic accuracy study evaluating a deep-learning AI system (Diagnocat) for detection of multiple dental conditions—including caries—on panoramic radiographs.	Digital panoramic radiographs (Planmeca Proline CC).	individual teeth	Standalone algorithm output	Commercial diagnostic and treatment-planning support intended for clinician review.	Tooth-level caries presence.	No
Park 2022	2022	South Korea	Prospective diagnostic accuracy study evaluating deep learning models (U-Net, ResNet-18, Faster R-CNN) for classification and localization of caries in intraoral photographic images obtained with a professional intraoral camera.	Intraoral photographic images (RGB) captured with a professional intraoral camera (Qraypen, AIOBIO, Seoul). Resolution: 1280×720 px	For classification → image<br>For localization → individual bounding-box carious lesion	Standalone algorithm output	Research/development evaluation of automated image classification and localization.	Distinct cavitated lesions, ICDAS 4-6.	No
Park 2023	2023	South Korea	Prospective diagnostic accuracy evaluation of a CNN (Xception architecture) for caries classification using quantitative light-induced fluorescence (QLF) intraoral photographic images, with and without manual tooth-surface segmentation.	QLF (Quantitative Light-Induced Fluorescence) intraoral photography. Device: Qraypen C® (AIOBIO, Seoul) Resolution: 1280×720 px	image (presence/absence of caries in the image).	Standalone algorithm output	Research/development evaluation of automated QLF-image classification.	Caries presence, ICDAS 3-6.	No
Zhang 2024	2024	China	Prospective clinical diagnostic accuracy study evaluating an AI system (MobileNet-v3 + U-Net) for tooth-level detection of caries using real-world intraoral photographic images from consecutive outpatients.	Device: A9X intraoral camera (Aicreate) Resolution: 1920×1080 px Three views per tooth: occlusal/incisal, buccal, palatal/lingual	tooth	Standalone algorithm output	Commercial camera-based screening/adjunctive support; algorithm output was evaluated independently against the reference standard.	Tooth-level caries presence using WHO criteria.	No

Part A. Study characteristics and diagnostic context (continued)

Report	Year	Country	Design	Imaging modality	Unit	Evaluated index-test construct	Intended-use context	Target-condition threshold	External validation
Chaudhari 2025	2025	India	Retrospective technical/diagnostic-accuracy study using a public image dataset (model development and internal test-set evaluation)	Periapical dental X-ray (radiograph)	Individual tooth/lesion instance (object-level bounding box detection within periapical images)	Standalone algorithm output	Research benchmark of automated multi-class object detection; no clinical workflow validated.	Caries object-detection class within a six-class task.	No
Caldwell 2026	2026	United States	Prospective diagnostic accuracy study of an AI caries-detection tool, paired with a before/after educational intervention study	Intraoral bitewing radiographs	Interproximal (proximal) tooth surfaces	Standalone algorithm output	Assistive/adjunctive caries detection and education; algorithm output was evaluated independently against the reference standard.	Enamel proximal caries (selected arm); dentinal caries reported separately.	No
Chen 2026	2026	China	Retrospective diagnostic accuracy study	CBCT (axial slices, cropped per-tooth ROIs)	tooth level (post-processed: caries positive requires prediction in ≥3 consecutive axial slices)	Standalone algorithm output	Adjunctive/triage-oriented CBCT pipeline; algorithm output was evaluated independently.	Tooth-level CBCT caries defined by enamel discontinuity/radiolucency or extension beyond the EDJ.	No
van Nistelrooij 2026	2026	Slovakia and Egypt	Retrospective diagnostic accuracy study	Mixed 2D dental radiographs: orthopantomograms (OPG), bitewings, periapical (PA), and other intraoral radiographs (caries in OPGs limited to moderate/severe lesions)	Individual tooth (single-tooth image crop)	Standalone algorithm output	Assistive decision-support pipeline whose outputs are intended for dentist confirmation.	Tooth-level caries-lesion presence; OPG labeling limited to moderate/severe lesions.	No
Giannakopoulos 2026	2026	Cyprus	Retrospective diagnostic accuracy study	Bitewing radiographs (digital, phosphor plate)	Tooth surface (approximal)	Standalone algorithm output	Commercial second-reader/diagnostic-support system; algorithm output was evaluated independently.	Any approximal caries, combining enamel and dentin lesions.	Yes
Rahimi 2026	2026	Iran	Retrospective dataset-benchmark diagnostic accuracy study	Panoramic radiograph (OPG), patch-based (64x64 px) from smile-zone crop	Image patch (64x64 pixels) extracted from smile-zone region	Standalone algorithm output	Research benchmark of 64 x 64-pixel patch classification; no clinically deployable observation unit validated.	Patch contains versus does not contain an annotated caries polygon.	No
Aarti-Rajambigai 2026	2026	NR	Prospective observational diagnostic accuracy study	Intraoral photographs and panoramic radiographs (plus clinical history text)	Patient submission (case-level, n=320 patients)	Standalone algorithm output	Clinician-supervised teledentistry decision support; algorithm output was evaluated independently against clinician consensus.	Reported active-caries versus non-caries subset.	No

Part A. Study characteristics and diagnostic context (continued)

Report	Year	Country	Design	Imaging modality	Unit	Evaluated index-test construct	Intended-use context	Target-condition threshold	External validation
Fadilah 2026	2026	Indonesia	Cross-sectional diagnostic accuracy study with prospective data collection and field validation	Intraoral clinical photographs (JPG, 640x640, taken with smartphones/tablets, ≥12MP)	ICDAS-labeled tooth surface/lesion instance, evaluated per ICDAS class (D1-D6) on new field-collected images	Standalone algorithm output	Field screening support with automated lesion detection; no single binary index-test threshold was selected.	ICDAS D1-D6 class-specific detection; no author-designated binary threshold.	Yes
Wang 2026	2026	China	Retrospective diagnostic accuracy study	Panoramic radiographs (digital, Planmeca ProMax / Sirona Orthophos XG)	Individual carious lesion (bounding-box detection with severity grade D1-D4); some analyses also reported at image/patient level	Standalone algorithm output	Assistive screening/decision support requiring professional verification.	Lesion detection with D1-D4 severity grading.	No
Liu 2026	2026	China	Prospective cross-sectional diagnostic accuracy study	Intraoral photographs (standardised device, occlusal and smooth surfaces)	Individual tooth surface images (occlusal and smooth), single-tooth crops	Standalone algorithm output	Population-level automated screening/triage rather than real-time clinician assistance.	Caries-free versus early versus moderate-to-severe caries.	No
Parekh 2026	2026	United States	Retrospective diagnostic accuracy study	Bitewing radiographs (intraoral, XDR sensors, 70 kVp/8 mA/0.16s)	Individual tooth-level observations (2,829 total annotated; 2657-2820 retained per finding after excluding teeth with observer disagreement)	Standalone algorithm output	Clinical adjunct/second-reader support; algorithm output was evaluated independently.	Tooth-level caries presence/absence.	Yes

Part A. Study characteristics and diagnostic context (continued)

Report	Year	Country	Design	Imaging modality	Unit	Evaluated index-test construct	Intended-use context	Target-condition threshold	External validation
Bicengil 2026	2026	Türkiye	Retrospective diagnostic accuracy study	Panoramic radiographs (paediatric, 5-12 years)	Lesion-level detections (bounding box, IoU≥0.50) per caries type (approximal, occlusal, buccal) in primary molars	Standalone algorithm output	Automated detector compared head-to-head with human readers; not evaluated as clinician plus AI.	Approximal, occlusal, or buccal caries in primary molars.	No
Abdelaziz 2026	2026	United Arab Emirates	Prospective cross-sectional diagnostic accuracy study	Standardized intraoral clinical photographs (Sony Alpha a7 IV with macro lens/flash)	Individual teeth/clinical features (caries/secondary caries lesions) within photographs, not per-patient	Standalone algorithm output	Clinical adjunct; authors did not recommend autonomous use, although algorithm output was evaluated independently.	Caries/secondary-caries clinical finding.	Yes
Kang 2026	2026	Republic of Korea	Pilot prospective diagnostic accuracy study	Self-acquired intraoral smartphone photographs	Tooth (tooth-level aggregation of predictions across images; annotated at tooth-surface visual instance level for training)	Standalone algorithm output	Patient-driven screening adjunct; algorithm output was evaluated independently.	Five-class tooth-level caries scheme.	No
Ren 2026	2026	NR	Retrospective diagnostic accuracy study (algorithm development/validation using public image datasets)	Intra-oral photographs and dental radiographs (bitewing/periapical)	Individual tooth images (image-level, not patient-level; 639 oral photographs, 456 radiographs)	Standalone algorithm output	Research/development evaluation of automated binary classification.	Caries versus healthy image.	No

Part B. Contingency data and selected estimate

Report	TP	FP	FN	TN	Sensitivity	Specificity	QUADAS-3 overall RoB	Selected estimate
Ahmed 2025	670	90	100	690	0.870	0.885	High	Single model (PointNetSeg), single test set, single threshold reported.
Arsiwala-Scheppach 2024							Not assessed	No accuracy estimate for the index test recoverable; gaze-behaviour outcomes only.
Bayraktar 2022	271	42	104	2283	0.723	0.982	High	Single YOLO detector; one test set of 2,700 approximal surfaces; one operating point.
Biltekin 2025	470	150	30	350	0.940	0.700	High	Multiple CNN backbones benchmarked; the authors' designated best-performing final model on the held-out test set of 1,000 tooth images was selected per rule R2.
Cao 2025							High	Multiple sub-tasks and three country sets; no single 2x2 table recoverable for the target condition.
Chen 2022	388	116	148	1435	0.724	0.925	High	Single Faster R-CNN; internal test set of 160 radiographs (2,087 surfaces); the authors' primary operating point.
Devlin 2021	591	422	189	2470	0.758	0.854	High	Two randomised arms; the AI-assisted arm was extracted because it is the arm in which the index test is applied. Analysed separately as clinician-plus-AI.
Estai 2022	1817	10063	0		1.000		High	ROI-level counts only; true negatives not recoverable.

Part B. Contingency data and selected estimate (continued)

Report	TP	FP	FN	TN	Sensitivity	Specificity	QUADAS-3 overall RoB	Selected estimate
Ma 2025							High	Segmentation/detection metrics only; no 2x2 table recoverable.
Mertens 2021							High	Design-effect adjusted reader data; no complete 2x2 for the assisted arm.
Orhan 2023	59	82	105	4274	0.360	0.981	High	Multi-label model; the caries output head was selected as the estimate matching our target condition per rule R1.
Park 2022	111	17	39	133	0.740	0.887	High	Single classification pipeline; whole-image test set (not the localisation sub-analysis) per rule R1.
Park 2023	423	53	64	273	0.869	0.837	High	Segmented and non-segmented test sets reported; the segmented test set (n=813) was selected as the authors' primary analysis per rule R3.
Zhang 2024	557	160	128	3516	0.813	0.956	High	Single model, single tooth-level threshold on the full clinical sample.
Chaudhari 2025	≈4*	≈9*	≈19*	≈68*	0.174	0.883	High	Approximate pseudo-cells reconstructed from rounded metrics; not directly reported integer counts and not used quantitatively.
Caldwell 2026	245	13	122	689	0.668	0.981	High	Enamel-caries arm from Table 1 (TP=245, FP=13, FN=122, TN=689), selected as the authors' principal algorithm-versus-reference-standard analysis. The separate dentinal-caries arm was not combined.

Part B. Contingency data and selected estimate (continued)

Report	TP	FP	FN	TN	Sensitivity	Specificity	QUADAS-3 overall RoB	Selected estimate
Chen 2026	13	2	13	114	0.500	0.983	High	DenseNet169 tooth-level caries arm. The reconstructed cells reproduce the reported sensitivity, specificity, predictive value, overall accuracy and balanced accuracy, supporting use of this estimate.
van Nistelrooij 2026	498	221	190	6416	0.724	0.967	High	Standalone algorithm-output result aggregated across radiograph modalities. Cells were reconstructed from the reported support, precision, sensitivity and specificity for the authors' principal model result.
Giannakopoulos 2026	141	77	52	1270	0.731	0.943	High	Any-caries arm (enamel and dentin combined) from Table 2. The reference-standard rows were oriented correctly; the selected cells reproduce all published operating characteristics.
Rahimi 2026	367	787	61	2815	0.857	0.782	High	Exact patch-level counts retained descriptively; excluded because correlated 64 x 64-pixel patches are not a clinically interpretable unit.
Aarti-Rajambigai 2026	≈187†	≈9†	≈10†	≈108†	0.949	0.923	High	Approximate reconstruction from rounded percentages; cells sum to 314 rather than the reported 320 participants and were not used quantitatively..
Fadilah 2026							High	Six ICDAS-specific rows were reported without a single author-designated binary caries table. The D1 row was recorded descriptively, but no complete primary 2x2 table was used.
Wang 2026							High	Authors proposed Transformer model recorded as the primary algorithm. A complete caries 2x2 table could not be recovered from the reported detection and severity metrics.

Part B. Contingency data and selected estimate (continued)

Report	TP	FP	FN	TN	Sensitivity	Specificity	QUADAS-3 overall RoB	Selected estimate
Liu 2026							High	Three-class caries result (caries-free, early, and moderate-to-severe) recorded descriptively. A binary threshold and exact class denominators were not reported, so no 2x2 table was reconstructed.
Parekh 2026							High	Caries arm recorded as the eligible target-condition result. Exact caries-specific denominators and contingency cells were not reported, so the result was not pooled.
Bicengil 2026							High	Overall pooled caries-detection arm recorded as the authors summary result. The published information did not support a fully verifiable contingency table for the selected arm.
Abdelaziz 2026							High	Caries/secondary-caries arm recorded as the primary caries outcome. Only percentages, confidence intervals and prevalence percentages were reported; raw arm-specific denominators were unavailable.
Kang 2026							High	Binary caries-versus-no-caries arm recorded as the primary scheme. The raw TP, FP, FN and TN matrix was not reported and could not be reconstructed uniquely.
Ren 2026							High	Primary dual-branch model recorded descriptively. Fold-level precision, recall, accuracy, F1 and AUC were reported without specificity or exact class sizes, so no 2x2 table was reconstructed.

Footnote. *Approximate pseudo-cells derived from rounded performance metrics and incompatible denominators. †Approximate cells reconstructed from rounded percentages; total = 314 rather than the stated n = 320. Approximation symbols appear in the cells as well as in the narrative note.

Part C. Estimate-selection rule and analytic disposition

Report	Selection rule	Analytic disposition	Reason for exclusion from exact 2 x 2 descriptive subset
Ahmed 2025	No choice required	Exact 2 x 2 descriptive subset	
Arsiwala-Scheppach 2024	Not applicable	Descriptive synthesis only	No distinct numerical DTA estimate; ancillary gaze-behaviour report of the Mertens 2021 reader trial. Not separately assessed in QUADAS-3 to avoid double counting.
Bayraktar 2022	No choice required	Exact 2 x 2 descriptive subset	
Biltekin 2025	Rule R2	Exact 2 x 2 descriptive subset	
Cao 2025	Not applicable	Descriptive synthesis only	No complete, internally coherent 2x2 table for calculation from exact contingency cells.
Chen 2022	No choice required	Exact 2 x 2 descriptive subset	
Devlin 2021	Rule R4	Secondary clinician-with-AI estimate	Different index-test construct (clinician plus AI); reported as a single secondary estimate and not combined with standalone AI.
Estai 2022	Not applicable	Descriptive synthesis only	No complete, internally coherent 2x2 table for calculation from exact contingency cells.
Ma 2025	Not applicable	Descriptive synthesis only	No complete, internally coherent 2x2 table for calculation from exact contingency cells.
Mertens 2021	Not applicable	Descriptive synthesis only	No complete, internally coherent 2x2 table for calculation from exact contingency cells.
Orhan 2023	Rule R1	Exact 2 x 2 descriptive subset	
Park 2022	Rule R1	Exact 2 x 2 descriptive subset	

Part C. Estimate-selection rule and analytic disposition (continued)

Report	Selection rule	Analytic disposition	Reason for exclusion from exact 2 x 2 descriptive subset
Park 2023	Rule R3	Exact 2 x 2 descriptive subset	
Zhang 2024	No choice required	Exact 2 x 2 descriptive subset	
Chaudhari 2025	R2 (primary arm designated by authors)	Descriptive synthesis only	Excluded from calculation from exact contingency cells: the contingency cells are not directly reported integer counts, depend on rounded reconstruction, and cannot be reconciled with the reported 100-image, 157-instance, and split-size denominators.
Caldwell 2026	R2 (primary arm designated by authors)	Exact 2 x 2 descriptive subset	
Chen 2026	R2 (primary arm designated by authors)	Exact 2 x 2 descriptive subset	
van Nistelrooij 2026	R2 (primary arm designated by authors)	Exact 2 x 2 descriptive subset	
Giannakopoulos 2026	R2 (primary arm designated by authors)	Exact 2 x 2 descriptive subset	
Rahimi 2026	R2 (primary arm designated by authors)	Descriptive synthesis only	Excluded from calculation from exact contingency cells: patch-level observations are not commensurate with clinically interpretable image-, tooth-, or surface-level diagnostic units and multiple correlated patches arise from each radiograph.
Aarti-Rajambigai 2026	R2 (primary arm designated by authors)	Descriptive synthesis only	Excluded from calculation from exact contingency cells: the 2x2 cells were reconstructed from rounded percentages, do not reconcile with the stated sample total, and do not provide a sufficiently verifiable standalone-AI contingency table.
Fadilah 2026	R2 (primary arm designated by authors)	Descriptive synthesis only	No complete, internally coherent 2x2 table for calculation from exact contingency cells.

Part C. Estimate-selection rule and analytic disposition (continued)

Report	Selection rule	Analytic disposition	Reason for exclusion from exact 2 x 2 descriptive subset
Wang 2026	R2 (primary arm designated by authors)	Descriptive synthesis only	No complete, internally coherent 2x2 table for calculation from exact contingency cells.
Liu 2026	R2 (primary arm designated by authors)	Descriptive synthesis only	No complete, internally coherent 2x2 table for calculation from exact contingency cells.
Parekh 2026	R2 (primary arm designated by authors)	Descriptive synthesis only	No complete, internally coherent 2x2 table for calculation from exact contingency cells.
Bicengil 2026	R2 (primary arm designated by authors)	Descriptive synthesis only	No complete, internally coherent 2x2 table for calculation from exact contingency cells.
Abdelaziz 2026	R2 (primary arm designated by authors)	Descriptive synthesis only	No complete, internally coherent 2x2 table for calculation from exact contingency cells.
Kang 2026	R2 (primary arm designated by authors)	Descriptive synthesis only	No complete, internally coherent 2x2 table for calculation from exact contingency cells.
Ren 2026	R2 (primary arm designated by authors)	Descriptive synthesis only	No complete, internally coherent 2x2 table for calculation from exact contingency cells.

Exact 2 x 2 descriptive subset denotes the 12 standalone reports used for calculated study-level sensitivity and specificity without pooling. Clinician-plus-AI evidence is summarized from controlled comparative results. Descriptive synthesis denotes inclusion without a complete exact contingency table.
