# Supplementary material for: Diagnostic Accuracy of Artificial Intelligence for Dental Caries Detection Across Clinical Imaging Modalities: A Systematic Review and Descriptive Synthesis
Source: Diagnostics (Basel). 2026 Sep 16;16(18):2995. doi: 10.3390/diagnostics16182995 (PMC13605960; doi:10.3390/diagnostics16182995)
Supplement: Supplementary file 1 [file diagnostics-16-02995-s001.zip › Supplementary_File_S4_Revised_QUADAS3_Assessment.pdf]

Supplementary File S4. Complete QUADAS-3 v1.2 Assessment

Diagnostic Accuracy of Artificial Intelligence for Dental Caries Detection across Clinical Imaging Modalities: A Systematic Review and Descriptive Synthesis

Purpose: This file documents the complete QUADAS-3 workflow used in the review: review-level synthesis questions, ideal test-accuracy studies, report flow, estimate selection, official signaling questions, estimate-level responses, domain judgments, applicability judgments, rationales, and source locations.

S4.1. Scope, response options, and decision rules

Item	Specification
Tool	QUADAS-3 version 1.2, applied at the numerical-estimate level.
Response options	Y = Yes; PY = Probably yes; PN = Probably no; N = No; NI = No information.
Judgement options	Low; High; II = Insufficient information.
Overall rule	Any High domain → overall High; all Low → overall Low; otherwise → II.
Supplement policy	No supplementary files were available. Their contents were not guessed. NI was assigned when the full text was insufficient.
Human verification	ACG and JM independently verified the pre-populated estimate-level matrix against the source reports on 26 August 2026. This was source verification of a common matrix, not de novo blinded duplicate coding. Final judgments are consensus verified; no inter-reviewer reliability statistic is claimed.
Linked report	Arsiwala-Scheppach 2024 is an ancillary gaze-behaviour report from the Mertens 2021 trial and provides no distinct numerical DTA estimate; it is documented in Phase 4 but not assessed in Phase 5.

S4.2. Review-level synthesis questions

ID	Clinical role	Synthesis question	Ideal test-accuracy study
Q1	Standalone AI	Among clinically acquired human dental images, what diagnostic accuracy is reported for a locked standalone artificial-intelligence system for detecting study-defined dental caries against a clinically appropriate reference standard?	Representative consecutive/random clinical population; clinically acquired human dental images; locked AI and positivity threshold fixed before an independent patient-level test; calibrated and blinded clinically appropriate reference standard with explicit lesion threshold; all observations accounted for; clinically meaningful unit; clustering handled.
Q2	AI-assisted clinician	Among clinicians interpreting human dental images, what diagnostic accuracy is reported when interpretation is assisted by an artificial-intelligence system, compared with the applicable reference standard?	Representative clinicians and clinical images; locked AI-assisted interpretation under a predefined protocol; independent calibrated reference standard; paired/randomized reading design where comparative value is assessed; all readers/images accounted for; crossed reader-image and patient clustering handled.

S4.3. Official signaling questions

Signal	Domain	Official QUADAS-3 wording
P1	Participants	Was a single-gate design used?
P2	Participants	Were participants prospectively enrolled?

Signal	Domain	Official QUADAS-3 wording
P3	Participants	Was a consecutive or random sample of participants included?
P4	Participants	Is the study group a representative sample of the intended-use population?
I1	Index	Was the index test conducted and interpreted according to the recommended instructions?
I2	Index	Were the index test results interpreted without knowledge of the reference standard results?
I3	Index	Were the index test results interpreted with the same information as would be available when the test is used in practice?
I4	Index	If an index test threshold was used, was it standard or pre-specified?
T1	Target	Does the reference standard adequately identify those with and without the target condition?
T2	Target	Was the target condition assessed in all participants?
T3	Target	Was the target condition assessed in the same way in all participants?
T4	Target	Did the reference standard avoid incorporating the index test?
T5	Target	Was the reference standard conducted and interpreted according to the recommended instructions?
T6	Target	Were the reference standard results interpreted without knowledge of the index test results?
T7	Target	If a reference standard threshold was used, was it standard or pre-specified?
T8	Target	Was there an appropriate interval between index test and reference standard?
A1	Analysis	Were all enrolled participants included in the analysis?
A2	Analysis	Were missing data handled appropriately?
A3	Analysis	Does the unit of analysis match the ideal test accuracy trial?
A4	Analysis	Were the estimates of sensitivity and specificity calculated appropriately?

Response coding: Y = Yes; PY = Probably yes; PN = Probably no; N = No; NI = No information. Domain judgments are Low, High, or II (insufficient information).

S4.4. Report-level estimate selection and disposition

Report–estimate reconciliation: The systematic review included 29 reports. Arsiwala-Scheppach 2024 was an ancillary report of the Mertens 2021 reader trial and did not contribute a distinct numerical diagnostic-accuracy estimate; consequently, 28 distinct estimates underwent QUADAS-3 Phase 5 assessment. Lam 2026 was excluded as a systematic review and is not part of the 29 included reports.

Report	Question	Numerical DTA estimate	Selected	Selected estimate	Numerical result	Selection rationale	Source
Ahmed 2025	Q1	Yes	Yes	Single model (PointNetSeg), single test set, single threshold reported.	TP=670; FP=90; FN=100; TN=690; sensitivity=0.870; specificity=0.885	Primary or most clinically relevant test-set estimate matching the assigned synthesis question; alternative models/thresholds were not treated as independent studies.	PDF pp. 1, 6
Arsiwala-Scheppach 2024	Context for Q2	No	No	Not reported	Not reported	Ancillary gaze-behaviour report of the Mertens 2021 trial; no distinct numerical sensitivity/specificity estimate. Excluded from estimate-level QUADAS-3 to avoid double counting.	PDF pp. 1–3

Report	Question	Numerical DTA estimate	Selected	Selected estimate	Numerical result	Selection rationale	Source
Bayraktar 2022	Q1	Yes	Yes	Single YOLO detector; one test set of 2,700 approximal surfaces; one operating point.	TP=271; FP=42; FN=104; TN=2283; sensitivity=0.723; specificity=0.982	Primary or most clinically relevant test-set estimate matching the assigned synthesis question; alternative models/thresholds were not treated as independent studies.	PDF pp. 6, 7
Biltekin 2025	Q1	Yes	Yes	Multiple CNN backbones benchmarked; the authors' designated best-performing final model on the held-out test set of 1,000 tooth images was selected per rule R2.	TP=470; FP=150; FN=30; TN=350; sensitivity=0.940; specificity=0.700	Primary or most clinically relevant test-set estimate matching the assigned synthesis question; alternative models/thresholds were not treated as independent studies.	PDF pp. 3, 4
Cao 2025	Q1	Yes	Yes	Multiple sub-tasks and three country sets; no single 2x2 table recoverable for the target condition.	Caries-lesion arm of Hierarchical Mask DINO: sensitivity=0.719; specificity=0.951; AUC=0.924.	Primary or most clinically relevant test-set estimate matching the assigned synthesis question; alternative models/thresholds were not treated as independent studies.	PDF pp. 4, 5
Chen 2022	Q1	Yes	Yes	Single Faster R-CNN; internal test set of 160 radiographs (2,087 surfaces); the authors' primary operating point.	TP=388; FP=116; FN=148; TN=1435; sensitivity=0.724; specificity=0.925	Primary or most clinically relevant test-set estimate matching the assigned synthesis question; alternative models/thresholds were not treated as independent studies.	PDF pp. 6, 7
Devlin 2021	Q2	Yes	Yes	Two randomised arms; the AI-assisted arm was extracted because it is the arm in which the index test is applied. Analysed separately as clinician-plus-AI.	TP=591; FP=422; FN=189; TN=2470; sensitivity=0.758; specificity=0.854	Primary or most clinically relevant test-set estimate matching the assigned synthesis question; alternative models/thresholds were not treated as independent studies.	PDF pp. 3, 4
Estai 2022	Q1	Yes	Yes	ROI-level counts only; true negatives not recoverable.	Inception-ResNet-v2 ROI task: sensitivity/recall=0.89; specificity=0.86.	Primary or most clinically relevant test-set estimate matching the assigned synthesis question; alternative models/thresholds were not treated as independent studies.	PDF pp. 2, 5
Ma 2025	Q1	Yes	Yes	Segmentation/detection metrics only; no 2x2 table recoverable.	Reported numerical estimate described in the selected-estimate field; no complete 2×2 table was reconstructed.	Primary or most clinically relevant test-set estimate matching the assigned synthesis question; alternative models/thresholds were not treated as independent studies.	PDF pp. 2, 11
Mertens 2021	Q2	Yes	Yes	Design-effect adjusted reader data; no complete 2x2 for the assisted arm.	AI-assisted readers: sensitivity=0.81; specificity approximately 0.97 (reported reader-study operating point).	Primary or most clinically relevant test-set estimate matching the assigned synthesis question; alternative models/thresholds were not treated as independent studies.	PDF pp. 3, 4
Orhan 2023	Q1	Yes	Yes	Multi-label model; the caries output head was selected as the estimate matching our target condition per rule R1.	TP=59; FP=82; FN=105; TN=4274; sensitivity=0.360; specificity=0.981	Primary or most clinically relevant test-set estimate matching the assigned synthesis question; alternative models/thresholds were not treated as independent studies.	PDF pp. 2, 6
Park 2022	Q1	Yes	Yes	Single classification pipeline; whole-image test set (not the localisation sub-analysis) per rule R1.	TP=111; FP=17; FN=39; TN=133; sensitivity=0.740; specificity=0.887	Primary or most clinically relevant test-set estimate matching the assigned synthesis question; alternative models/thresholds were not treated as independent studies.	PDF pp. 5, 6
Park 2023	Q1	Yes	Yes	Segmented and non-segmented test sets reported; the segmented test set (n=813) was selected as the authors' primary analysis per rule R3.	TP=423; FP=53; FN=64; TN=273; sensitivity=0.869; specificity=0.837	Primary or most clinically relevant test-set estimate matching the assigned synthesis question; alternative models/thresholds were not treated as independent studies.	PDF pp. 1, 4
Zhang 2024	Q1	Yes	Yes	Single model, single tooth-level threshold on the full clinical sample.	TP=557; FP=160; FN=128; TN=3516; sensitivity=0.813; specificity=0.956	Primary or most clinically relevant test-set estimate matching the assigned synthesis question; alternative models/thresholds were not treated as independent studies.	PDF pp. 1, 3

Report	Question	Numerical DTA estimate	Selected	Selected estimate	Numerical result	Selection rationale	Source
Chaudhari 2025	Q1	Yes	Yes	Counts extracted for the Caries class arm (the review's target condition) from Table 3; values were reported as non-integer decimals (likely back-calculated by authors from sensitivity/specificity and class instance counts) and were rounded to nearest integer here. Test set = 100 images with 157 tot	TP=4; FP=9; FN=19; TN=68; sensitivity=0.174; specificity=0.883	Primary or most clinically relevant test-set estimate matching the assigned synthesis question; alternative models/thresholds were not treated as independent studies.	PDF pp. 2, 7
Caldwell 2026	Q1	Yes	Yes	Counts are verbatim Table 1, enamel-caries arm (245/689/13/122), giving Se 66.8%, Sp 98.1%; the abstract quotes 69%/99%, a minor discrepancy attributable to rounding/arm aggregation. Table counts retained as the authoritative source. Table 1 gives two separate standalone-AI arms (Enamel caries and D	TP=245; FP=13; FN=122; TN=689; sensitivity=0.668; specificity=0.981	Primary or most clinically relevant test-set estimate matching the assigned synthesis question; alternative models/thresholds were not treated as independent studies.	PDF pp. 2, 4
Chen 2026	Q1	Yes	Yes	Counts back-calculated from DenseNet169's reported sensitivity=0.5000, specificity=0.9828, PPV=0.8667, NPV=0.8976, which independently reproduce the reported overall accuracy (0.8944) and balanced accuracy (0.7414) exactly, supporting reconstruction validity. This is the tooth-level standalone carie	TP=13; FP=2; FN=13; TN=114; sensitivity=0.500; specificity=0.983	Primary or most clinically relevant test-set estimate matching the assigned synthesis question; alternative models/thresholds were not treated as independent studies.	PDF pp. 6, 10
van Nistelrooij 2026	Q1	Yes	Yes	Extracted the model (standalone AI, 5-fold cross-validation mean) arm for 'Caries lesion' from Table 3/4, the authors' primary aggregate result across all radiograph modalities (support=688 diseased teeth = TP+FN). TP/FP/FN/TN reconstructed by combining reported precision, sensitivity, specificity a	TP=498; FP=221; FN=190; TN=6416; sensitivity=0.724; specificity=0.967	Primary or most clinically relevant test-set estimate matching the assigned synthesis question; alternative models/thresholds were not treated as independent studies.	PDF pp. 5, 8
Giannakopoulos 2026	Q1	Yes	Yes	Table 2 is oriented reference-standard-in-rows; the initial extraction transposed FP/FN. Corrected assignment reproduces all four published operating characteristics exactly (Se 73.1%, Sp 94.3%, PPV 64.7%, NPV 96.1%, accuracy 91.6%). Primary arm extracted is 'any caries' (enamel+dentin combined), Ta	TP=141; FP=77; FN=52; TN=1270; sensitivity=0.731; specificity=0.943	Primary or most clinically relevant test-set estimate matching the assigned synthesis question; alternative models/thresholds were not treated as independent studies.	PDF pp. 5, 7
Rahimi 2026	Q1	Yes	Yes	Extracted the independent (held-out, non-cross-validated) test-set arm of the patch classifier, the authors' primary standalone-AI evaluation (5-fold CV results also reported but not used here). TP/FP/FN/TN reconstructed from reported Recall=0.857, Precision=0.318, total caries patches=428, and tota	TP=367; FP=787; FN=61; TN=2815; sensitivity=0.857; specificity=0.782	Primary or most clinically relevant test-set estimate matching the assigned synthesis question; alternative models/thresholds were not treated as independent studies.	PDF pp. 12, 13
Aarti Rajambigai 2026	Q1	Yes	Yes	Only the caries-detection arm was extracted per eligibility criteria (periodontitis and malocclusion arms reported but out of scope). FN and FP were derived algebraically from reported TP=187, TN=108, sensitivity=94.8%, specificity=92.1%; reconstructed total (314) is close to but not exactly 320, in	TP=187; FP=9; FN=10; TN=108; sensitivity=0.949; specificity=0.923	Primary or most clinically relevant test-set estimate matching the assigned synthesis question; alternative models/thresholds were not treated as independent studies.	PDF pp. 2, 3

Report	Question	Numerical DTA estimate	Selected	Selected estimate	Numerical result	Selection rationale	Source
Fadilah 2026	Q1	Yes	Yes	Paper reports six separate per-ICDAS-class (D1-D6) SEN/SPE/PPV/NPV values for AI vs. dentists (Table 5), with no single overall/pooled standalone-AI 2x2 designated as primary; D1 (any-caries threshold) row extracted as illustrative but not used for counts. Sample size is internally inconsistent (tex	AI, ICDAS D1 field-validation row: sensitivity=0.9080; specificity=0.9017.	Primary or most clinically relevant test-set estimate matching the assigned synthesis question; alternative models/thresholds were not treated as independent studies.	PDF pp. 10, 11
Wang 2026	Q1	Yes	Yes	Primary standalone-AI arm extracted is the proposed Transformer model's overall test-set performance vs expert consensus (the paper's headline comparison), not the six baseline CNN/DETR/Swin comparator arms in Table 4 or the AI-assisted-clinician framing mentioned only in Discussion. A full 2x2 coul	Proposed Transformer, overall independent test set: sensitivity=0.884; specificity=0.917.	Primary or most clinically relevant test-set estimate matching the assigned synthesis question; alternative models/thresholds were not treated as independent studies.	PDF pp. 3, 7
Liu 2026	Q1	Yes	Yes	Study reports a three-class classification (caries-free/early/moderate-severe) per tooth surface (occlusal, smooth) rather than binary diagnostic 2x2 outcomes, so TP/FP/FN/TN cannot be reconstructed without assuming a specific positive-class threshold and knowing exact class sample sizes (not report	Reported numerical estimate described in the selected-estimate field; no complete 2×2 table was reconstructed.	Primary or most clinically relevant test-set estimate matching the assigned synthesis question; alternative models/thresholds were not treated as independent studies.	PDF pp. 4, 7
Parekh 2026	Q1	Yes	Yes	Caries is one of four arms (caries, restorations, prosthesis, implant); caries extracted as the target condition for this review. Only sensitivity/specificity are reported per finding, with a stated range of 2657-2820 tooth-level observations post-filtration across all four findings combined, but ex	Caries arm after human-consensus filtration: sensitivity=0.972; specificity=0.842.	Primary or most clinically relevant test-set estimate matching the assigned synthesis question; alternative models/thresholds were not treated as independent studies.	PDF pp. 1, 4
Bicengil 2026	Q1	Yes	Yes	Multiple lesion-type arms (approximal, occlusal, buccal) and an overall pooled arm reported; extracted the overall pooled standalone-AI arm as primary since authors present it as the summary comparison (Table 2/Section 3.4). TP=477 stated directly; FP and FN back-calculated from precision=TP/(TP+FP)	Reported numerical estimate described in the selected-estimate field; no complete 2×2 table was reconstructed.	Primary or most clinically relevant test-set estimate matching the assigned synthesis question; alternative models/thresholds were not treated as independent studies.	PDF pp. 11, 12
Abdelaziz 2026	Q1	Yes	Yes	Extracted the 'Caries/secondary caries (C)' arm as primary caries outcome (ICDAS reference); a second caries-related arm 'Pit and fissure caries/stain (P)' is also reported but not selected as primary. Only percentages with CIs and prevalence percentages are given in Table 1 (no raw denominators per	Caries/secondary-caries arm: sensitivity=0.3303; specificity=0.9571.	Primary or most clinically relevant test-set estimate matching the assigned synthesis question; alternative models/thresholds were not treated as independent studies.	PDF pp. 4, 7
Kang 2026	Q1	Yes	Yes	Three arms reported (binary, four-class, five-class classification schemes) using the same YOLOv6-L6 model; extracted the binary classification arm as primary since it is the simplest caries-vs-no-caries standalone AI comparison and authors present it first in Table 5. Raw TP/FP/FN/TN confusion matr	Prespecified five-class scheme: sensitivity=0.86; specificity=0.9845; accuracy=0.936.	Primary or most clinically relevant test-set estimate matching the assigned synthesis question; alternative models/thresholds were not treated as independent studies.	PDF pp. 2, 10

Report	Question	Numerical DTA estimate	Selected	Selected estimate	Numerical result	Selection rationale	Source
Ren 2026	Q1	Yes	Yes	Only precision/recall/accuracy/F1/AUC per fold on a held-out 20% test set are reported (mean ± SD across 5-fold CV repeated 10 times); no raw TP/FP/FN/TN counts or specificity are given, and class sizes per fold/test split are not stated, so a 2x2 table cannot be reconstructed. Extracted the primary	Reported numerical estimate described in the selected-estimate field; no complete 2×2 table was reconstructed.	Primary or most clinically relevant test-set estimate matching the assigned synthesis question; alternative models/thresholds were not treated as independent studies.	PDF pp. 3, 16

S4.5. Summary of estimate-level judgments

Estimate	Q	Participants RoB	Index test RoB	Target condition RoB	Analysis RoB	Overall RoB	Participants applicability	Index applicability	Target applicability	Overall applicability
Ahmed 2025	Q1	II	Low	Low	High	High	Low	Low	Low	Low
Bayraktar 2022	Q1	High	Low	II	High	High	Low	Low	II	II
Biltekin 2025	Q1	High	Low	II	High	High	High	Low	Low	High
Cao 2025	Q1	Low	Low	II	High	High	Low	Low	Low	Low
Chen 2022	Q1	High	Low	Low	High	High	High	Low	Low	High
Estai 2022	Q1	High	Low	High	High	High	High	Low	II	High
Ma 2025	Q1	High	Low	Low	High	High	High	High	Low	High
Orhan 2023	Q1	II	II	Low	High	High	Low	Low	II	II
Park 2022	Q1	High	Low	High	High	High	High	High	High	High
Park 2023	Q1	High	Low	High	High	High	High	High	II	High
Zhang 2024	Q1	Low	Low	Low	High	High	Low	Low	Low	Low
Chaudhari 2025	Q1	High	High	High	High	High	High	Low	II	High
Caldwell 2026	Q1	High	High	Low	High	High	High	Low	Low	High
Chen 2026	Q1	High	High	High	Low	High	High	High	Low	High
van Nistelrooij 2026	Q1	High	II	II	High	High	Low	Low	Low	Low
Giannakopoulos 2026	Q1	High	Low	Low	Low	High	High	Low	Low	High
Rahimi 2026	Q1	High	II	II	High	High	Low	High	Low	High
Aarti Rajambigai 2026	Q1	II	II	II	High	High	II	Low	Low	II
Fadilah 2026	Q1	High	II	II	High	High	Low	Low	Low	Low
Wang 2026	Q1	High	High	Low	High	High	Low	Low	Low	Low
Liu 2026	Q1	Low	Low	II	High	High	Low	Low	Low	Low
Parekh 2026	Q1	High	II	Low	High	High	High	Low	Low	High
Bicengil 2026	Q1	High	Low	Low	High	High	Low	Low	Low	Low
Abdelaziz 2026	Q1	High	Low	II	High	High	Low	High	Low	High
Kang 2026	Q1	High	High	High	High	High	High	High	II	High
Ren 2026	Q1	High	II	Low	High	High	High	High	II	High

Estimate	Q	Participants RoB	Index test RoB	Target condition RoB	Analysis RoB	Overall RoB	Participants applicability	Index applicability	Target applicability	Overall applicability
Mertens 2021	Q2	High	Low	Il	Low	High	High	Low	Il	High
Devlin 2021	Q2	High	Low	Low	High	High	High	Low	High	High

Reconciliation: Twenty-eight distinct numerical estimates were assessed. Overall risk of bias was High in 28/28 estimates. Analysis risk of bias was High in 25/28 and Low in 3/28. The summary counts reproduce Figure 2 and Supplementary Table S1.

S4.6. Complete estimate-level assessments

Reading guide: Each subsection reports the selected numerical estimate, source-to-analysis flow, all 20 signaling responses, and the final risk-of-bias and applicability judgments with rationales. No unreported method was inferred from an unavailable supplementary file.

S4.6.1. Ahmed 2025 (Q1)

Assessment element	Details
Design	Prospective clinical trial (diagnostic performance evaluation)
Source-to-analysis flow	Source cohort/data set (Prospective clinical trial (diagnostic performance evaluation)) → eligibility/selection as reported → development split(s), if applicable → selected independent/cross-validated test set: Approximately 10% → ~6–7 scans → diagnostic analysis at Tooth surface (3D point-cloud units from IOS) level. Any unreported exclusions or indeterminate/missing observations were coded NI in Phase 5.
Reported evaluation set	Approximately 10% → ~6–7 scans
Analysis unit	Tooth surface (3D point-cloud units from IOS)
Selected estimate	Single model (PointNetSeg), single test set, single threshold reported.
Numerical result	TP=670; FP=90; FN=100; TN=690; sensitivity=0.870; specificity=0.885
Selection rule	Primary or most clinically relevant test-set estimate matching the assigned synthesis question; alternative models/thresholds were not treated as independent studies.
Sources	Flow: PDF pp. 2, 3; estimate selection: PDF pp. 1, 6

Signal	Response	Official signaling question
P1	Y	Was a single-gate design used?
P2	Y	Were participants prospectively enrolled?
P3	NI	Was a consecutive or random sample of participants included?
P4	PY	Is the study group a representative sample of the intended-use population?
I1	Y	Was the index test conducted and interpreted according to the recommended instructions?
I2	PY	Were the index test results interpreted without knowledge of the reference standard results?
I3	Y	Were the index test results interpreted with the same information as would be available when the test is used in practice?
I4	Y	If an index test threshold was used, was it standard or pre-specified?
T1	PY	Does the reference standard adequately identify those with and without the target condition?
T2	Y	Was the target condition assessed in all participants?
T3	Y	Was the target condition assessed in the same way in all participants?

Signal	Response	Official signaling question
T4	Y	Did the reference standard avoid incorporating the index test?
T5	Y	Was the reference standard conducted and interpreted according to the recommended instructions?
T6	PY	Were the reference standard results interpreted without knowledge of the index test results?
T7	Y	If a reference standard threshold was used, was it standard or pre-specified?
T8	Y	Was there an appropriate interval between index test and reference standard?
A1	PY	Were all enrolled participants included in the analysis?
A2	PY	Were missing data handled appropriately?
A3	Y	Does the unit of analysis match the ideal test accuracy trial?
A4	N	Were the estimates of sensitivity and specificity calculated appropriately?

Domain	Risk of bias	Applicability	Rationale	Source
Participants	II	Low	Prospective clinical trial (diagnostic performance evaluation). Test set: Approximately 10% → ~6–7 scans. The full text did not provide enough information on sampling/representativeness for a definitive judgement.	PDF pp. 2, 3
Index test	Low	Low	Index test: PointNetSeg (3D point-cloud segmentation network; STN3d + STNkd + 1D conv layers). A locked test evaluation and sufficiently prespecified interpretation were reported.	PDF pp. 3, 5
Target condition	Low	Low	Reference standard: Modified ICDAS score assigned by calibrated dentists. The reference procedure was judged sufficiently appropriate and independent for the study-defined target condition.	PDF pp. 2, 3
Analysis	High	Not applicable	The study-level test estimate was retained, but the small held-out set and unmodelled within-scan/tooth dependence make uncertainty and effective sample size unreliable.	PDF pp. 1, 6
Overall	High	Low	Overall risk of bias followed the prespecified rule: any High domain yields an overall High judgment; all Low yields Low; otherwise II. Overall applicability reflects the three applicability domains.	Derived from the domain judgments above

Source-status note: Full-text article reviewed. NI was used wherever the article did not report sufficient information.

S4.6.2. Bayraktar 2022 (Q1)

Assessment element	Details
Design	Retrospective diagnostic accuracy study (CNN development + internal testing)
Source-to-analysis flow	Source cohort/data set (Retrospective diagnostic accuracy study (CNN development + internal testing)) → eligibility/selection as reported → development split(s), if applicable → selected independent/cross-validated test set: 200 radiographs → diagnostic analysis at Approximal tooth surfaces (premolars and molars) level. Any unreported exclusions or indeterminate/missing observations were coded NI in Phase 5.
Reported evaluation set	200 radiographs
Analysis unit	Approximal tooth surfaces (premolars and molars)
Selected estimate	Single YOLO detector; one test set of 2,700 approximal surfaces; one operating point.
Numerical result	TP=271; FP=42; FN=104; TN=2283; sensitivity=0.723; specificity=0.982
Selection rule	Primary or most clinically relevant test-set estimate matching the assigned synthesis question; alternative models/thresholds were not treated as independent studies.
Sources	Flow: PDF pp. 2, 5; estimate selection: PDF pp. 6, 7

Signal	Response	Official signaling question
P1	Y	Was a single-gate design used?
P2	N	Were participants prospectively enrolled?
P3	PN	Was a consecutive or random sample of participants included?
P4	PN	Is the study group a representative sample of the intended-use population?
I1	Y	Was the index test conducted and interpreted according to the recommended instructions?
I2	PY	Were the index test results interpreted without knowledge of the reference standard results?
I3	PY	Were the index test results interpreted with the same information as would be available when the test is used in practice?
I4	Y	If an index test threshold was used, was it standard or pre-specified?
T1	PY	Does the reference standard adequately identify those with and without the target condition?
T2	Y	Was the target condition assessed in all participants?
T3	Y	Was the target condition assessed in the same way in all participants?
T4	Y	Did the reference standard avoid incorporating the index test?
T5	PY	Was the reference standard conducted and interpreted according to the recommended instructions?
T6	NI	Were the reference standard results interpreted without knowledge of the index test results?
T7	PY	If a reference standard threshold was used, was it standard or pre-specified?
T8	Y	Was there an appropriate interval between index test and reference standard?
A1	PY	Were all enrolled participants included in the analysis?
A2	PY	Were missing data handled appropriately?
A3	Y	Does the unit of analysis match the ideal test accuracy trial?
A4	N	Were the estimates of sensitivity and specificity calculated appropriately?

Domain	Risk of bias	Applicability	Rationale	Source
Participants	High	Low	Retrospective diagnostic accuracy study (CNN development + internal testing). Test set: 200 radiographs. Selection, enrichment, case-control construction, exclusions, or limited spectrum could materially bias accuracy.	PDF pp. 2, 5

Domain	Risk of bias	Applicability	Rationale	Source
Index test	Low	Low	Index test: Modified YOLOv3 (DarkNet-53 feature extractor; 106 convolutional layers; multi-scale detection heads). A locked test evaluation and sufficiently prespecified interpretation were reported.	PDF pp. 2, 5
Target condition	II	II	Reference standard: Two expert restorative dentists (>10 years experience) labeled lesions via consensus ("fuzzy gold standard" with bounding boxes). Blinding, calibration, reliability, or threshold details were insufficiently reported; missing supplements were not imputed.	PDF pp. 3, 4
Analysis	High	Not applicable	Per-tooth results were analysed without accounting for teeth clustered within radiographs/patients.	PDF pp. 6, 7
Overall	High	II	Overall risk of bias followed the prespecified rule: any High domain yields an overall High judgment; all Low yields Low; otherwise II. Overall applicability reflects the three applicability domains.	Derived from the domain judgments above

Source-status note: Full-text article reviewed. NI was used wherever the article did not report sufficient information.

S4.6.3. Biltekin 2025 (Q1)

Assessment element	Details
Design	Retrospective diagnostic accuracy study (DL model development and internal testing)
Source-to-analysis flow	Source cohort/data set (Retrospective diagnostic accuracy study (DL model development and internal testing)) → eligibility/selection as reported → development split(s), if applicable → selected independent/cross-validated test set: 1,000 images (500 sound + 500 carious) → diagnostic analysis at Individual teeth (cropped tooth images) level. Any unreported exclusions or indeterminate/missing observations were coded NI in Phase 5.
Reported evaluation set	1,000 images (500 sound + 500 carious)
Analysis unit	Individual teeth (cropped tooth images)
Selected estimate	Multiple CNN backbones benchmarked; the authors' designated best-performing final model on the held-out test set of 1,000 tooth images was selected per rule R2.
Numerical result	TP=470; FP=150; FN=30; TN=350; sensitivity=0.940; specificity=0.700
Selection rule	Primary or most clinically relevant test-set estimate matching the assigned synthesis question; alternative models/thresholds were not treated as independent studies.
Sources	Flow: PDF pp. 1, 2; estimate selection: PDF pp. 3, 4

Signal	Response	Official signaling question
P1	N	Was a single-gate design used?
P2	N	Were participants prospectively enrolled?
P3	N	Was a consecutive or random sample of participants included?
P4	N	Is the study group a representative sample of the intended-use population?
I1	Y	Was the index test conducted and interpreted according to the recommended instructions?
I2	PY	Were the index test results interpreted without knowledge of the reference standard results?
I3	PY	Were the index test results interpreted with the same information as would be available when the test is used in practice?
I4	Y	If an index test threshold was used, was it standard or pre-specified?
T1	PY	Does the reference standard adequately identify those with and without the target condition?
T2	Y	Was the target condition assessed in all participants?
T3	Y	Was the target condition assessed in the same way in all participants?
T4	Y	Did the reference standard avoid incorporating the index test?
T5	PY	Was the reference standard conducted and interpreted according to the recommended instructions?
T6	NI	Were the reference standard results interpreted without knowledge of the index test results?
T7	PY	If a reference standard threshold was used, was it standard or pre-specified?
T8	Y	Was there an appropriate interval between index test and reference standard?
A1	N	Were all enrolled participants included in the analysis?
A2	N	Were missing data handled appropriately?
A3	Y	Does the unit of analysis match the ideal test accuracy trial?
A4	N	Were the estimates of sensitivity and specificity calculated appropriately?

Domain	Risk of bias	Applicability	Rationale	Source
Participants	High	High	Retrospective diagnostic accuracy study (DL model development and internal testing). Test set: 1,000 images (500 sound + 500 carious). Selection, enrichment, case-control construction, exclusions, or limited spectrum could materially bias accuracy.	PDF pp. 1, 2

Domain	Risk of bias	Applicability	Rationale	Source
Index test	Low	Low	Index test: – ResNet50 backbone – Modified architecture with Gaussian Context Transformer (GCT) channel-attention block (GCT-ResNet50-b0). A locked test evaluation and sufficiently prespecified interpretation were reported.	PDF pp. 3, 4
Target condition	II	Low	Reference standard: Consensus of two oral and maxillofacial radiologists + one radiology resident. Disagreements led to exclusion of image. Blinding, calibration, reliability, or threshold details were insufficiently reported; missing supplements were not imputed.	PDF p. 2
Analysis	High	Not applicable	Balanced image sampling, unexplained exclusions and image-level analysis prevent a complete intention-to-analyse estimate.	PDF pp. 3, 4
Overall	High	High	Overall risk of bias followed the prespecified rule: any High domain yields an overall High judgment; all Low yields Low; otherwise II. Overall applicability reflects the three applicability domains.	Derived from the domain judgments above

Source-status note: Full-text article reviewed. NI was used wherever the article did not report sufficient information.

S4.6.4. Cao 2025 (Q1)

Assessment element	Details
Design	Retrospective multi-center AI development + external validation study (instance segmentation and multi-class classification)
Source-to-analysis flow	Source cohort/data set (Retrospective multi-center AI development + external validation study (instance segmentation and multi-class classification)) → eligibility/selection as reported → development split(s), if applicable → selected independent/cross-validated test set: 216 → diagnostic analysis at – Teeth (instance segmentation) – Per-tooth multi-class semantic segmentation (seven conditions) level. Any unreported exclusions or indeterminate/missing observations were coded NI in Phase 5.
Reported evaluation set	216
Analysis unit	– Teeth (instance segmentation) – Per-tooth multi-class semantic segmentation (seven conditions)
Selected estimate	Multiple sub-tasks and three country sets; no single 2x2 table recoverable for the target condition.
Numerical result	Caries-lesion arm of Hierarchical Mask DINO: sensitivity=0.719; specificity=0.951; AUC=0.924.
Selection rule	Primary or most clinically relevant test-set estimate matching the assigned synthesis question; alternative models/thresholds were not treated as independent studies.
Sources	Flow: PDF pp. 2, 8; estimate selection: PDF pp. 4, 5

Signal	Response	Official signaling question
P1	Y	Was a single-gate design used?
P2	N	Were participants prospectively enrolled?
P3	PY	Was a consecutive or random sample of participants included?
P4	Y	Is the study group a representative sample of the intended-use population?
I1	Y	Was the index test conducted and interpreted according to the recommended instructions?
I2	Y	Were the index test results interpreted without knowledge of the reference standard results?
I3	Y	Were the index test results interpreted with the same information as would be available when the test is used in practice?
I4	Y	If an index test threshold was used, was it standard or pre-specified?
T1	PY	Does the reference standard adequately identify those with and without the target condition?
T2	Y	Was the target condition assessed in all participants?
T3	Y	Was the target condition assessed in the same way in all participants?
T4	Y	Did the reference standard avoid incorporating the index test?
T5	PY	Was the reference standard conducted and interpreted according to the recommended instructions?
T6	NI	Were the reference standard results interpreted without knowledge of the index test results?
T7	PY	If a reference standard threshold was used, was it standard or pre-specified?
T8	Y	Was there an appropriate interval between index test and reference standard?
A1	PY	Were all enrolled participants included in the analysis?
A2	PY	Were missing data handled appropriately?
A3	Y	Does the unit of analysis match the ideal test accuracy trial?
A4	N	Were the estimates of sensitivity and specificity calculated appropriately?

Domain	Risk of bias	Applicability	Rationale	Source
Participants	Low	Low	Retrospective multi-center AI development + external validation study (instance segmentation and multi-class classification). Test set: 216. Sampling and spectrum were sufficiently aligned with the review question for this domain.	PDF pp. 2, 8
Index test	Low	Low	Index test: Hierarchical Mask DINO. A locked test evaluation and sufficiently prespecified interpretation were reported.	PDF pp. 3, 8

Domain	Risk of bias	Applicability	Rationale	Source
Target condition	II	Low	Reference standard: Pixel-level multi-expert consensus annotations. Blinding, calibration, reliability, or threshold details were insufficiently reported; missing supplements were not imputed.	PDF pp. 2, 8
Analysis	High	Not applicable	Tooth-level estimates did not account for teeth clustered within patients/images.	PDF pp. 4, 5
Overall	High	Low	Overall risk of bias followed the prespecified rule: any High domain yields an overall High judgment; all Low yields Low; otherwise II. Overall applicability reflects the three applicability domains.	Derived from the domain judgments above

Source-status note: Full-text article reviewed. NI was used wherever the article did not report sufficient information.

S4.6.5. Chen 2022 (Q1)

Assessment element	Details
Design	Retrospective diagnostic accuracy study involving development and testing of a Faster R-CNN object detection model for identifying proximal caries lesions on bitewing radiographs. The model's performance was compared against postgraduate dental students.
Source-to-analysis flow	Source cohort/data set (Retrospective diagnostic accuracy study involving development and testing of a Faster R-CNN object detection model for identifying proximal caries lesions on bitewing radiographs. The model's performance was compared against postgraduate dental students.) → eligibility/selection as reported → development split(s), if applicable → selected independent/cross-validated test set: 160 BW radiographs → diagnostic analysis at Individual proximal surfaces on posterior teeth (mesial and distal surfaces assessed independently). level. Any unreported exclusions or indeterminate/missing observations were coded NI in Phase 5.
Reported evaluation set	160 BW radiographs
Analysis unit	Individual proximal surfaces on posterior teeth (mesial and distal surfaces assessed independently).
Selected estimate	Single Faster R-CNN; internal test set of 160 radiographs (2,087 surfaces); the authors' primary operating point.
Numerical result	TP=388; FP=116; FN=148; TN=1435; sensitivity=0.724; specificity=0.925
Selection rule	Primary or most clinically relevant test-set estimate matching the assigned synthesis question; alternative models/thresholds were not treated as independent studies.
Sources	Flow: PDF pp. 1, 2; estimate selection: PDF pp. 6, 7

Signal	Response	Official signaling question
P1	N	Was a single-gate design used?
P2	N	Were participants prospectively enrolled?
P3	Y	Was a consecutive or random sample of participants included?
P4	PN	Is the study group a representative sample of the intended-use population?
I1	Y	Was the index test conducted and interpreted according to the recommended instructions?
I2	Y	Were the index test results interpreted without knowledge of the reference standard results?
I3	Y	Were the index test results interpreted with the same information as would be available when the test is used in practice?
I4	Y	If an index test threshold was used, was it standard or pre-specified?
T1	Y	Does the reference standard adequately identify those with and without the target condition?
T2	Y	Was the target condition assessed in all participants?
T3	Y	Was the target condition assessed in the same way in all participants?
T4	Y	Did the reference standard avoid incorporating the index test?
T5	Y	Was the reference standard conducted and interpreted according to the recommended instructions?
T6	Y	Were the reference standard results interpreted without knowledge of the index test results?
T7	Y	If a reference standard threshold was used, was it standard or pre-specified?
T8	Y	Was there an appropriate interval between index test and reference standard?
A1	Y	Were all enrolled participants included in the analysis?
A2	PY	Were missing data handled appropriately?
A3	Y	Does the unit of analysis match the ideal test accuracy trial?
A4	N	Were the estimates of sensitivity and specificity calculated appropriately?

Domain	Risk of bias	Applicability	Rationale	Source

Domain	Risk of bias	Applicability	Rationale	Source
Participants	High	High	Retrospective diagnostic accuracy study involving development and testing of a Faster R-CNN object detection model for identifying proximal caries lesions on bitewing radiographs. The model's performance was compared against postgraduate dental students. Test set: 160 BW radiographs. Selection, enrichment, case-control construction, exclusions, or limited spectrum could materially bias accuracy.	PDF pp. 1, 2
Index test	Low	Low	Index test: Faster R-CNN with: Convolutional backbone (ImageNet pretrained) Region Proposal Network (RPN) Multi-stage bounding-box generation Adam optimizer, binary focal loss Trained for 200 epochs (24 hours) on NVIDIA Tesla P40 GPU Framework: PaddlePaddle (Python). A locked test evaluation and sufficiently prespecified interpretation were reported.	PDF pp. 4, 8
Target condition	Low	Low	Reference standard: Labelers: – Two endodontists – One radiologist (≥5 years experience) Annotation software: LabelMe Procedure: – Independent labeling – Consensus resolution for disagreements – Pre-calibration of observers – Bounding-box labeling Lesion staging: E1, E2, D1, D2, D3; later merged into E1/E2, D1, D2/D3 categories. The reference procedure was judged sufficiently appropriate and independent for the study-defined target condition.	PDF pp. 3, 4
Analysis	High	Not applicable	Image/tooth observations were treated as independent despite within-patient clustering.	PDF pp. 6, 7
Overall	High	High	Overall risk of bias followed the prespecified rule: any High domain yields an overall High judgment; all Low yields Low; otherwise II. Overall applicability reflects the three applicability domains.	Derived from the domain judgments above

Source-status note: Full-text article reviewed. NI was used wherever the article did not report sufficient information.

S4.6.6. Estai 2022 (Q1)

Assessment element	Details
Design	Retrospective diagnostic accuracy study evaluating a two-stage deep learning system (ROI detection via Faster R-CNN + caries classification via Inception-ResNet-v2) for automatic detection of proximal caries on digital bitewing radiographs, compared against expert-generated reference standard.
Source-to-analysis flow	Source cohort/data set (Retrospective diagnostic accuracy study evaluating a two-stage deep learning system (ROI detection via Faster R-CNN + caries classification via Inception-ResNet-v2) for automatic detection of proximal caries on digital bitewing radiographs, compared against expert-generated reference standard.) → eligibility/selection as reported → development split(s), if applicable → selected independent/cross-validated test set: No separate external test partition (performance evaluated through 10-fold cross-validation) → diagnostic analysis at Region of interest (ROI) corresponding to proximal surface. level. Any unreported exclusions or indeterminate/missing observations were coded NI in Phase 5.
Reported evaluation set	No separate external test partition (performance evaluated through 10-fold cross-validation)
Analysis unit	Region of interest (ROI) corresponding to proximal surface.
Selected estimate	ROI-level counts only; true negatives not recoverable.
Numerical result	Inception-ResNet-v2 ROI task: sensitivity/recall=0.89; specificity=0.86.
Selection rule	Primary or most clinically relevant test-set estimate matching the assigned synthesis question; alternative models/thresholds were not treated as independent studies.
Sources	Flow: PDF pp. 1, 4; estimate selection: PDF pp. 2, 5

Signal	Response	Official signaling question
P1	N	Was a single-gate design used?
P2	N	Were participants prospectively enrolled?
P3	PN	Was a consecutive or random sample of participants included?
P4	PN	Is the study group a representative sample of the intended-use population?
I1	Y	Was the index test conducted and interpreted according to the recommended instructions?
I2	PY	Were the index test results interpreted without knowledge of the reference standard results?
I3	PY	Were the index test results interpreted with the same information as would be available when the test is used in practice?
I4	Y	If an index test threshold was used, was it standard or pre-specified?
T1	PN	Does the reference standard adequately identify those with and without the target condition?
T2	Y	Was the target condition assessed in all participants?
T3	Y	Was the target condition assessed in the same way in all participants?
T4	Y	Did the reference standard avoid incorporating the index test?
T5	PY	Was the reference standard conducted and interpreted according to the recommended instructions?
T6	NI	Were the reference standard results interpreted without knowledge of the index test results?
T7	Y	If a reference standard threshold was used, was it standard or pre-specified?
T8	Y	Was there an appropriate interval between index test and reference standard?
A1	PY	Were all enrolled participants included in the analysis?
A2	PY	Were missing data handled appropriately?
A3	PN	Does the unit of analysis match the ideal test accuracy trial?
A4	N	Were the estimates of sensitivity and specificity calculated appropriately?

Domain	Risk of bias	Applicability	Rationale	Source

Domain	Risk of bias	Applicability	Rationale	Source
Participants	High	High	Retrospective diagnostic accuracy study evaluating a two-stage deep learning system (ROI detection via Faster R-CNN + caries classification via Inception-ResNet-v2) for automatic detection of proximal caries on digital bitewing radiographs, compared against expert-generated reference standard. Test set: No separate external test partition (performance evaluated through 10-fold cross-validation). Selection, enrichment, case-control construction, exclusions, or limited spectrum could materially bias accuracy.	PDF pp. 1, 4
Index test	Low	Low	Index test: ROI Detection: Faster R-CNN (with VGG-16 backbone) Pretrained on ImageNet Fine-tuning of top 70% layers Caries Classification: Inception-ResNet-v2 Compared with Inception-v3 and ResNet-50 Pretrained weights (ImageNet) Fine-tuning of 80% layers Best performance among the three CNNs tested. A locked test evaluation and sufficiently prespecified interpretation were reported.	PDF pp. 2, 4
Target condition	High	II	Reference standard: Three qualified dentists (>10 years experience): Two dentists independently labeled all images Disagreements resolved by a third dentist Classification criteria based on WHO, ICDAS, and Mejåre depth categories Interexaminer reliability: κ = 0.60 (moderate agreement). The reference procedure or its reliability was inadequate enough to risk systematic misclassification.	PDF pp. 2, 3
Analysis	High	Not applicable	Cross-validation and region-of-interest sampling did not preserve a fully independent clinical test cohort and did not address dependence between observations.	PDF pp. 2, 5
Overall	High	High	Overall risk of bias followed the prespecified rule: any High domain yields an overall High judgment; all Low yields Low; otherwise II. Overall applicability reflects the three applicability domains.	Derived from the domain judgments above

Source-status note: Full-text article reviewed. NI was used wherever the article did not report sufficient information.

S4.6.7. Ma 2025 (Q1)

Assessment element	Details
Design	Retrospective AI-development and internal validation study evaluating performance of Mask R-CNN for detecting and segmenting teeth and dental caries on cone-beam computed tomography (CBCT) images. Three hyperparameter groups were compared: R group – no transfer learning, random initialization I group – transfer learning from ImageNet C group – transfer learning from COCO
Source-to-analysis flow	Source cohort/data set (Retrospective AI-development and internal validation study evaluating performance of Mask R-CNN for detecting and segmenting teeth and dental caries on cone-beam computed tomography (CBCT) images. Three hyperparameter groups were compared: R group – no transfer learning, random initialization I group – transfer learning from ImageNet C group – transfer learning from COCO) → eligibility/selection as reported → development split(s), if applicable → selected independent/cross-validated test set: 213 images → diagnostic analysis at Entire tooth volume / tooth-level and pixel-level regions of interest (segmentations). Each CBCT image contains multiple teeth and possibly carious regions. level. Any unreported exclusions or indeterminate/missing observations were coded NI in Phase 5.
Reported evaluation set	213 images
Analysis unit	Entire tooth volume / tooth-level and pixel-level regions of interest (segmentations). Each CBCT image contains multiple teeth and possibly carious regions.
Selected estimate	Segmentation/detection metrics only; no 2x2 table recoverable.
Numerical result	Reported numerical estimate described in the selected-estimate field; no complete 2×2 table was reconstructed.
Selection rule	Primary or most clinically relevant test-set estimate matching the assigned synthesis question; alternative models/thresholds were not treated as independent studies.
Sources	Flow: PDF pp. 5, 12; estimate selection: PDF pp. 2, 11

Signal	Response	Official signaling question
P1	PY	Was a single-gate design used?
P2	N	Were participants prospectively enrolled?
P3	PN	Was a consecutive or random sample of participants included?
P4	PN	Is the study group a representative sample of the intended-use population?
I1	Y	Was the index test conducted and interpreted according to the recommended instructions?
I2	PY	Were the index test results interpreted without knowledge of the reference standard results?
I3	PN	Were the index test results interpreted with the same information as would be available when the test is used in practice?
I4	Y	If an index test threshold was used, was it standard or pre-specified?
T1	Y	Does the reference standard adequately identify those with and without the target condition?
T2	Y	Was the target condition assessed in all participants?
T3	Y	Was the target condition assessed in the same way in all participants?
T4	Y	Did the reference standard avoid incorporating the index test?
T5	Y	Was the reference standard conducted and interpreted according to the recommended instructions?
T6	PY	Were the reference standard results interpreted without knowledge of the index test results?
T7	Y	If a reference standard threshold was used, was it standard or pre-specified?
T8	Y	Was there an appropriate interval between index test and reference standard?
A1	PY	Were all enrolled participants included in the analysis?
A2	PY	Were missing data handled appropriately?
A3	Y	Does the unit of analysis match the ideal test accuracy trial?
A4	N	Were the estimates of sensitivity and specificity calculated appropriately?

Domain	Risk of bias	Applicability	Rationale	Source

Domain	Risk of bias	Applicability	Rationale	Source
Participants	High	High	Retrospective AI-development and internal validation study evaluating performance of Mask R-CNN for detecting and segmenting teeth and dental caries on cone-beam computed tomography (CBCT) images. Three hyperparameter groups were compared: R group – no transfer learning, random initialization I group – transfer learning from ImageNet C group – transfer learning from COCO. Test set: 213 images. Selection, enrichment, case-control construction, exclusions, or limited spectrum could materially bias accuracy.	PDF pp. 5, 12
Index test	Low	High	Index test: AI_Architecture: Mask R-CNN with: Backbone: ResNet-50 Feature Pyramid Network (FPN) Region Proposal Network (RPN) RoIAlign Fully Convolutional Network (mask branch) Losses: L_cls + L_bbox + L_mask Three weight initialization strategies: R: Random I: ImageNet-based pretraining C: COCO-based pretraining Training: 300 epochs (100, 200, 300 sampled) Performed on Tesla V100 GPU (32 GB VRAM), 230 GB RAM, Python 3.6, TensorFlow/Keras. A locked test evaluation and sufficiently prespecified interpretation were reported.	PDF pp. 5, 6
Target condition	Low	Low	Reference standard: Two oral and maxillofacial radiologists with 10 years of experience Segmented teeth and carious regions manually WHO diagnostic criteria Caries: low-density concave defects in enamel/dentin Multi-plane refinement Boolean mask subtraction for isolating caries region. The reference procedure was judged sufficiently appropriate and independent for the study-defined target condition.	PDF pp. 3, 6
Analysis	High	Not applicable	Cropped tooth/region observations were treated as independent and clustering was not incorporated in the accuracy analysis.	PDF pp. 2, 11
Overall	High	High	Overall risk of bias followed the prespecified rule: any High domain yields an overall High judgment; all Low yields Low; otherwise II. Overall applicability reflects the three applicability domains.	Derived from the domain judgments above

Source-status note: Full-text article reviewed. NI was used wherever the article did not report sufficient information.

S4.6.8. Orhan 2023 (Q1)

Assessment element	Details
Design	Retrospective diagnostic accuracy study evaluating a deep-learning AI system (Diagnocat) for detection of multiple dental conditions—including caries—on panoramic radiographs.
Source-to-analysis flow	Source cohort/data set (Retrospective diagnostic accuracy study evaluating a deep-learning AI system (Diagnocat) for detection of multiple dental conditions—including caries—on panoramic radiographs.) → eligibility/selection as reported → development split(s), if applicable → selected independent/cross-validated test set: 100 PRs → diagnostic analysis at individual teeth level. Any unreported exclusions or indeterminate/missing observations were coded NI in Phase 5.
Reported evaluation set	100 PRs
Analysis unit	individual teeth
Selected estimate	Multi-label model; the caries output head was selected as the estimate matching our target condition per rule R1.
Numerical result	TP=59; FP=82; FN=105; TN=4274; sensitivity=0.360; specificity=0.981
Selection rule	Primary or most clinically relevant test-set estimate matching the assigned synthesis question; alternative models/thresholds were not treated as independent studies.
Sources	Flow: PDF pp. 2, 4; estimate selection: PDF pp. 2, 6

Signal	Response	Official signaling question
P1	Y	Was a single-gate design used?
P2	N	Were participants prospectively enrolled?
P3	NI	Was a consecutive or random sample of participants included?
P4	PY	Is the study group a representative sample of the intended-use population?
I1	PY	Was the index test conducted and interpreted according to the recommended instructions?
I2	PY	Were the index test results interpreted without knowledge of the reference standard results?
I3	Y	Were the index test results interpreted with the same information as would be available when the test is used in practice?
I4	NI	If an index test threshold was used, was it standard or pre-specified?
T1	Y	Does the reference standard adequately identify those with and without the target condition?
T2	Y	Was the target condition assessed in all participants?
T3	Y	Was the target condition assessed in the same way in all participants?
T4	Y	Did the reference standard avoid incorporating the index test?
T5	Y	Was the reference standard conducted and interpreted according to the recommended instructions?
T6	PY	Were the reference standard results interpreted without knowledge of the index test results?
T7	Y	If a reference standard threshold was used, was it standard or pre-specified?
T8	Y	Was there an appropriate interval between index test and reference standard?
A1	PY	Were all enrolled participants included in the analysis?
A2	NI	Were missing data handled appropriately?
A3	Y	Does the unit of analysis match the ideal test accuracy trial?
A4	N	Were the estimates of sensitivity and specificity calculated appropriately?

Domain	Risk of bias	Applicability	Rationale	Source
Participants	II	Low	Retrospective diagnostic accuracy study evaluating a deep-learning AI system (Diagnocat) for detection of multiple dental conditions—including caries—on panoramic radiographs. Test set: 100 PRs. The full text did not provide enough information on sampling/representativeness for a definitive judgement.	PDF pp. 2, 4

Domain	Risk of bias	Applicability	Rationale	Source
Index test	II	Low	Index test: Diagnocat (proprietary pipeline integrating): ROI detector (Mask R-CNN, ResNet-101 backbone) Tooth detection → segmentation masks Cascade R-CNN for pathology detection Multi-head architecture detecting 21 conditions Pseudo-labeling and mosaic augmentation used 5430 PRs + 18000 intraoral X-rays for training. Threshold prespecification, blinding, or exact test-use instructions were incompletely reported.	PDF pp. 4, 5
Target condition	Low	II	Reference standard: Three dentomaxillofacial radiologists, independently reviewing images, were calibrated and their majority vote was the ground truth. The reference procedure was judged sufficiently appropriate and independent for the study-defined target condition.	PDF pp. 1, 6
Analysis	High	Not applicable	Tooth-level estimates from panoramic radiographs did not account for teeth clustered within a radiograph/patient; handling of missing/ungradable observations was not reported.	PDF pp. 2, 6
Overall	High	II	Overall risk of bias followed the prespecified rule: any High domain yields an overall High judgment; all Low yields Low; otherwise II. Overall applicability reflects the three applicability domains.	Derived from the domain judgments above

Source-status note: Full-text article reviewed. NI was used wherever the article did not report sufficient information.

S4.6.9. Park 2022 (Q1)

Assessment element	Details
Design	Prospective diagnostic accuracy study evaluating deep learning models (U-Net, ResNet-18, Faster R-CNN) for classification and localization of caries in intraoral photographic images obtained with a professional intraoral camera.
Source-to-analysis flow	Source cohort/data set (Prospective diagnostic accuracy study evaluating deep learning models (U-Net, ResNet-18, Faster R-CNN) for classification and localization of caries in intraoral photographic images obtained with a professional intraoral camera.) → eligibility/selection as reported → development split(s), if applicable → selected independent/cross-validated test set: 300 images → diagnostic analysis at For classification → image For localization → individual bounding-box carious lesion level. Any unreported exclusions or indeterminate/missing observations were coded NI in Phase 5.
Reported evaluation set	300 images
Analysis unit	For classification → image For localization → individual bounding-box carious lesion
Selected estimate	Single classification pipeline; whole-image test set (not the localisation sub-analysis) per rule R1.
Numerical result	TP=111; FP=17; FN=39; TN=133; sensitivity=0.740; specificity=0.887
Selection rule	Primary or most clinically relevant test-set estimate matching the assigned synthesis question; alternative models/thresholds were not treated as independent studies.
Sources	Flow: PDF pp. 2, 3; estimate selection: PDF pp. 5, 6

Signal	Response	Official signaling question
P1	N	Was a single-gate design used?
P2	Y	Were participants prospectively enrolled?
P3	PN	Was a consecutive or random sample of participants included?
P4	N	Is the study group a representative sample of the intended-use population?
I1	Y	Was the index test conducted and interpreted according to the recommended instructions?
I2	PY	Were the index test results interpreted without knowledge of the reference standard results?
I3	PN	Were the index test results interpreted with the same information as would be available when the test is used in practice?
I4	Y	If an index test threshold was used, was it standard or pre-specified?
T1	N	Does the reference standard adequately identify those with and without the target condition?
T2	Y	Was the target condition assessed in all participants?
T3	Y	Was the target condition assessed in the same way in all participants?
T4	Y	Did the reference standard avoid incorporating the index test?
T5	PN	Was the reference standard conducted and interpreted according to the recommended instructions?
T6	NI	Were the reference standard results interpreted without knowledge of the index test results?
T7	Y	If a reference standard threshold was used, was it standard or pre-specified?
T8	Y	Was there an appropriate interval between index test and reference standard?
A1	PY	Were all enrolled participants included in the analysis?
A2	PY	Were missing data handled appropriately?
A3	PN	Does the unit of analysis match the ideal test accuracy trial?
A4	N	Were the estimates of sensitivity and specificity calculated appropriately?

Domain	Risk of bias	Applicability	Rationale	Source

Domain	Risk of bias	Applicability	Rationale	Source
Participants	High	High	Prospective diagnostic accuracy study evaluating deep learning models (U-Net, ResNet-18, Faster R-CNN) for classification and localization of caries in intraoral photographic images obtained with a professional intraoral camera. Test set: 300 images. Selection, enrichment, case-control construction, exclusions, or limited spectrum could materially bias accuracy.	PDF pp. 2, 3
Index test	Low	High	Index test: AI_Architecture: Three-stage pipeline: U-Net → segmentation of tooth surface (pixel-wise) ResNet-18 → classification (caries vs non-caries) Faster R-CNN → localization (bounding-box detection) Details: Pretrained on ImageNet (ResNet) and COCO (Faster R-CNN) 5-fold cross-validation Data augmentation: shifts, symmetry, blurring Optimizer: SGD; LR=0,001 IoU threshold adjusted to 0,2 (low due to irregular caries borders). A locked test evaluation and sufficiently prespecified interpretation were reported.	PDF pp. 3, 5
Target condition	High	High	Reference standard: Expert board-certified dentist in epidemiologic oral examination (Korean government certification) Pixel-wise annotation for tooth surfaces Bounding-box annotation for caries ICDAS codes 4, 5, 6 used (distinct cavitated lesions). The reference procedure or its reliability was inadequate enough to risk systematic misclassification.	PDF pp. 3, 5
Analysis	High	Not applicable	The analysed unit and selected spectrum differed from the intended clinical unit, and clustered observations were not handled.	PDF pp. 5, 6
Overall	High	High	Overall risk of bias followed the prespecified rule: any High domain yields an overall High judgment; all Low yields Low; otherwise II. Overall applicability reflects the three applicability domains.	Derived from the domain judgments above

Source-status note: Full-text article reviewed. NI was used wherever the article did not report sufficient information.

S4.6.10. Park 2023 (Q1)

Assessment element	Details
Design	Prospective diagnostic accuracy evaluation of a CNN (Xception architecture) for caries classification using quantitative light-induced fluorescence (QLF) intraoral photographic images, with and without manual tooth-surface segmentation.
Source-to-analysis flow	Source cohort/data set (Prospective diagnostic accuracy evaluation of a CNN (Xception architecture) for caries classification using quantitative light-induced fluorescence (QLF) intraoral photographic images, with and without manual tooth-surface segmentation.) → eligibility/selection as reported → development split(s), if applicable → selected independent/cross-validated test set: 845 → diagnostic analysis at image (presence/absence of caries in the image). level. Any unreported exclusions or indeterminate/missing observations were coded NI in Phase 5.
Reported evaluation set	845
Analysis unit	image (presence/absence of caries in the image).
Selected estimate	Segmented and non-segmented test sets reported; the segmented test set (n=813) was selected as the authors' primary analysis per rule R3.
Numerical result	TP=423; FP=53; FN=64; TN=273; sensitivity=0.869; specificity=0.837
Selection rule	Primary or most clinically relevant test-set estimate matching the assigned synthesis question; alternative models/thresholds were not treated as independent studies.
Sources	Flow: PDF pp. 3, 6; estimate selection: PDF pp. 1, 4

Signal	Response	Official signaling question
P1	PY	Was a single-gate design used?
P2	Y	Were participants prospectively enrolled?
P3	PN	Was a consecutive or random sample of participants included?
P4	PN	Is the study group a representative sample of the intended-use population?
I1	Y	Was the index test conducted and interpreted according to the recommended instructions?
I2	PY	Were the index test results interpreted without knowledge of the reference standard results?
I3	PN	Were the index test results interpreted with the same information as would be available when the test is used in practice?
I4	Y	If an index test threshold was used, was it standard or pre-specified?
T1	PN	Does the reference standard adequately identify those with and without the target condition?
T2	Y	Was the target condition assessed in all participants?
T3	Y	Was the target condition assessed in the same way in all participants?
T4	Y	Did the reference standard avoid incorporating the index test?
T5	PN	Was the reference standard conducted and interpreted according to the recommended instructions?
T6	NI	Were the reference standard results interpreted without knowledge of the index test results?
T7	Y	If a reference standard threshold was used, was it standard or pre-specified?
T8	Y	Was there an appropriate interval between index test and reference standard?
A1	N	Were all enrolled participants included in the analysis?
A2	NI	Were missing data handled appropriately?
A3	Y	Does the unit of analysis match the ideal test accuracy trial?
A4	N	Were the estimates of sensitivity and specificity calculated appropriately?

Domain	Risk of bias	Applicability	Rationale	Source
Participants	High	High	Prospective diagnostic accuracy evaluation of a CNN (Xception architecture) for caries classification using quantitative light-induced fluorescence (QLF) intraoral photographic images, with and without manual tooth-surface segmentation. Test set: 845. Selection, enrichment, case-control construction, exclusions, or limited spectrum could materially bias accuracy.	PDF pp. 3, 6

Domain	Risk of bias	Applicability	Rationale	Source
Index test	Low	High	Index test: Xception CNN Pretrained on ImageNet Fine-tuning Augmentations: flips, rotations, brightness, contrast, blurring Resizing to 612×420 px Adam optimizer Learning rate: 0,00001 5-fold cross-validation 15–20 epochs with early stopping. A locked test evaluation and sufficiently prespecified interpretation were reported.	PDF pp. 3, 6
Target condition	High	II	Reference standard: One dentist trained in ICDAS ICDAS codes 3–6 defined as caries White-light images used as clinical reference No consensus or multi-examiner review. The reference procedure or its reliability was inadequate enough to risk systematic misclassification.	PDF p. 3
Analysis	High	Not applicable	Not all eligible observations were transparently accounted for and clustering/missing-data handling was not reported.	PDF pp. 1, 4
Overall	High	High	Overall risk of bias followed the prespecified rule: any High domain yields an overall High judgment; all Low yields Low; otherwise II. Overall applicability reflects the three applicability domains.	Derived from the domain judgments above

Source-status note: Full-text article reviewed; . NI was used wherever the article did not report sufficient information.

S4.6.11. Zhang 2024 (Q1)

Assessment element	Details
Design	Prospective clinical diagnostic accuracy study evaluating an AI system (MobileNet-v3 + U-Net) for tooth-level detection of caries using real-world intraoral photographic images from consecutive outpatients.
Source-to-analysis flow	Source cohort/data set (Prospective clinical diagnostic accuracy study evaluating an AI system (MobileNet-v3 + U-Net) for tooth-level detection of caries using real-world intraoral photographic images from consecutive outpatients.) → eligibility/selection as reported → development split(s), if applicable → selected independent/cross-validated test set: NR → diagnostic analysis at tooth level. Any unreported exclusions or indeterminate/missing observations were coded NI in Phase 5.
Reported evaluation set	NR
Analysis unit	tooth
Selected estimate	Single model, single tooth-level threshold on the full clinical sample.
Numerical result	TP=557; FP=160; FN=128; TN=3516; sensitivity=0.813; specificity=0.956
Selection rule	Primary or most clinically relevant test-set estimate matching the assigned synthesis question; alternative models/thresholds were not treated as independent studies.
Sources	Flow: PDF pp. 2, 5; estimate selection: PDF pp. 1, 3

Signal	Response	Official signaling question
P1	Y	Was a single-gate design used?
P2	Y	Were participants prospectively enrolled?
P3	Y	Was a consecutive or random sample of participants included?
P4	Y	Is the study group a representative sample of the intended-use population?
I1	Y	Was the index test conducted and interpreted according to the recommended instructions?
I2	Y	Were the index test results interpreted without knowledge of the reference standard results?
I3	Y	Were the index test results interpreted with the same information as would be available when the test is used in practice?
I4	Y	If an index test threshold was used, was it standard or pre-specified?
T1	Y	Does the reference standard adequately identify those with and without the target condition?
T2	Y	Was the target condition assessed in all participants?
T3	Y	Was the target condition assessed in the same way in all participants?
T4	Y	Did the reference standard avoid incorporating the index test?
T5	Y	Was the reference standard conducted and interpreted according to the recommended instructions?
T6	Y	Were the reference standard results interpreted without knowledge of the index test results?
T7	Y	If a reference standard threshold was used, was it standard or pre-specified?
T8	Y	Was there an appropriate interval between index test and reference standard?
A1	Y	Were all enrolled participants included in the analysis?
A2	Y	Were missing data handled appropriately?
A3	Y	Does the unit of analysis match the ideal test accuracy trial?
A4	N	Were the estimates of sensitivity and specificity calculated appropriately?

Domain	Risk of bias	Applicability	Rationale	Source
Participants	Low	Low	Prospective clinical diagnostic accuracy study evaluating an AI system (MobileNet-v3 + U-Net) for tooth-level detection of caries using real-world intraoral photographic images from consecutive outpatients. Test set: NR. Sampling and spectrum were sufficiently aligned with the review question for this domain.	PDF pp. 2, 5

Domain	Risk of bias	Applicability	Rationale	Source
Index test	Low	Low	Index test: Backbone: MobileNet-v3 Segmentation: 5-layer U-Net Over 150000 expert-annotated intraoral images used for training 15% reserved for testing Data augmentation: rotation, cropping, brightness, chromaticity, translation Optimizer: Adam Dynamic decay learning rate (0,99) Input images resized to 512×512 px. A locked test evaluation and sufficiently prespecified interpretation were reported.	PDF pp. 2, 5
Target condition	Low	Low	Reference standard: Two independent endodontists (5 years experience each) Conflicts resolved by an expert (>20 years experience) WHO 5th edition criteria Blinded to AI output. The reference procedure was judged sufficiently appropriate and independent for the study-defined target condition.	PDF pp. 1, 3
Analysis	High	Not applicable	Although sampling and reference procedures were strong, the reported tooth/surface-level accuracy analysis did not model clustering within patients.	PDF pp. 1, 3
Overall	High	Low	Overall risk of bias followed the prespecified rule: any High domain yields an overall High judgment; all Low yields Low; otherwise II. Overall applicability reflects the three applicability domains.	Derived from the domain judgments above

Source-status note: Full-text article reviewed; . NI was used wherever the article did not report sufficient information.

S4.6.12. Chaudhari 2025 (Q1)

Assessment element	Details
Design	Retrospective technical/diagnostic-accuracy study using a public image dataset (model development and internal test-set evaluation)
Source-to-analysis flow	Source cohort/data set (Retrospective technical/diagnostic-accuracy study using a public image dataset (model development and internal test-set evaluation)) → eligibility/selection as reported → development split(s), if applicable → selected independent/cross-validated test set: 100 test images / 157 instances (Table 2, abstract); Methods section separately states the dataset split as 1729 training, 55 test, and 100 validation images — internally inconsistent → diagnostic analysis at Individual tooth/lesion instance (object-level bounding box detection within periapical images) level. Any unreported exclusions or indeterminate/missing observations were coded NI in Phase 5.
Reported evaluation set	100 test images / 157 instances (Table 2, abstract); Methods section separately states the dataset split as 1729 training, 55 test, and 100 validation images — internally inconsistent
Analysis unit	Individual tooth/lesion instance (object-level bounding box detection within periapical images)
Selected estimate	Counts extracted for the Caries class arm (the review's target condition) from Table 3; values were reported as non-integer decimals (likely back-calculated by authors from sensitivity/specificity and class instance counts) and were rounded to nearest integer here. Test set = 100 images with 157 tot
Numerical result	TP=4; FP=9; FN=19; TN=68; sensitivity=0.174; specificity=0.883
Selection rule	Primary or most clinically relevant test-set estimate matching the assigned synthesis question; alternative models/thresholds were not treated as independent studies.
Sources	Flow: PDF pp. 3, 5; estimate selection: PDF pp. 2, 7

Signal	Response	Official signaling question
P1	N	Was a single-gate design used?
P2	N	Were participants prospectively enrolled?
P3	NI	Was a consecutive or random sample of participants included?
P4	N	Is the study group a representative sample of the intended-use population?
I1	N	Was the index test conducted and interpreted according to the recommended instructions?
I2	PY	Were the index test results interpreted without knowledge of the reference standard results?
I3	PN	Were the index test results interpreted with the same information as would be available when the test is used in practice?
I4	NI	If an index test threshold was used, was it standard or pre-specified?
T1	N	Does the reference standard adequately identify those with and without the target condition?
T2	NI	Was the target condition assessed in all participants?
T3	NI	Was the target condition assessed in the same way in all participants?
T4	PY	Did the reference standard avoid incorporating the index test?
T5	N	Was the reference standard conducted and interpreted according to the recommended instructions?
T6	NI	Were the reference standard results interpreted without knowledge of the index test results?
T7	NI	If a reference standard threshold was used, was it standard or pre-specified?
T8	Y	Was there an appropriate interval between index test and reference standard?
A1	PN	Were all enrolled participants included in the analysis?
A2	NI	Were missing data handled appropriately?
A3	PN	Does the unit of analysis match the ideal test accuracy trial?
A4	N	Were the estimates of sensitivity and specificity calculated appropriately?

Domain	Risk of bias	Applicability	Rationale	Source

Domain	Risk of bias	Applicability	Rationale	Source
Participants	High	High	Retrospective technical/diagnostic-accuracy study using a public image dataset (model development and internal test-set evaluation). Test set: 100 test images / 157 instances (Table 2, abstract); Methods section separately states the dataset split as 1729 training, 55 test, and 100 validation images — internally inconsistent. Selection, enrichment, case-control construction, exclusions, or limited spectrum could materially bias accuracy.	PDF pp. 3, 5
Index test	High	Low	Index test: ESRGAN (RRDB-based super-resolution preprocessing) + YOLOv8 (Ultralytics v8.1.0, yolov8n backbone, one-stage object detector) for 6-class dental condition detection. Model/threshold selection, non-independent evaluation, or access to reference-related information could materially bias performance.	PDF pp. 6, 7
Target condition	High	II	Reference standard: Expert-annotated COCO-format bounding box ground-truth labels on the publicly available Kaggle Dental Periapical X-rays dataset, manually spot-checked by the authors. The reference procedure or its reliability was inadequate enough to risk systematic misclassification.	PDF p. 3
Analysis	High	Not applicable	Dataset counts were internally inconsistent and the reported instance/image denominators could not be reconciled; clustering and exclusions were not handled.	PDF pp. 2, 7
Overall	High	High	Overall risk of bias followed the prespecified rule: any High domain yields an overall High judgment; all Low yields Low; otherwise II. Overall applicability reflects the three applicability domains.	Derived from the domain judgments above

Source-status note: Full-text article reviewed. NI was used wherever the article did not report sufficient information.

S4.6.13. Caldwell 2026 (Q1)

Assessment element	Details
Design	Prospective diagnostic accuracy study of an AI caries-detection tool, paired with a before/after educational intervention study
Source-to-analysis flow	Source cohort/data set (Prospective diagnostic accuracy study of an AI caries-detection tool, paired with a before/after educational intervention study) → eligibility/selection as reported → development split(s), if applicable → selected independent/cross-validated test set: 1,604 proximal surfaces across 400 bitewing images (200 images/1,142 surfaces read for enamel caries and 200 images read for dentinal caries by two examiners) → diagnostic analysis at Interproximal (proximal) tooth surfaces level. Any unreported exclusions or indeterminate/missing observations were coded NI in Phase 5.
Reported evaluation set	1,604 proximal surfaces across 400 bitewing images (200 images/1,142 surfaces read for enamel caries and 200 images read for dentinal caries by two examiners)
Analysis unit	Interproximal (proximal) tooth surfaces
Selected estimate	Counts are verbatim Table 1, enamel-caries arm (245/689/13/122), giving Se 66.8%, Sp 98.1%; the abstract quotes 69%/99%, a minor discrepancy attributable to rounding/arm aggregation. Table counts retained as the authoritative source. Table 1 gives two separate standalone-AI arms (Enamel caries and D
Numerical result	TP=245; FP=13; FN=122; TN=689; sensitivity=0.668; specificity=0.981
Selection rule	Primary or most clinically relevant test-set estimate matching the assigned synthesis question; alternative models/thresholds were not treated as independent studies.
Sources	Flow: PDF pp. 3, 7; estimate selection: PDF pp. 2, 4

Signal	Response	Official signaling question
P1	PY	Was a single-gate design used?
P2	Y	Were participants prospectively enrolled?
P3	NI	Was a consecutive or random sample of participants included?
P4	PN	Is the study group a representative sample of the intended-use population?
I1	Y	Was the index test conducted and interpreted according to the recommended instructions?
I2	N	Were the index test results interpreted without knowledge of the reference standard results?
I3	Y	Were the index test results interpreted with the same information as would be available when the test is used in practice?
I4	Y	If an index test threshold was used, was it standard or pre-specified?
T1	Y	Does the reference standard adequately identify those with and without the target condition?
T2	Y	Was the target condition assessed in all participants?
T3	Y	Was the target condition assessed in the same way in all participants?
T4	Y	Did the reference standard avoid incorporating the index test?
T5	Y	Was the reference standard conducted and interpreted according to the recommended instructions?
T6	PY	Were the reference standard results interpreted without knowledge of the index test results?
T7	Y	If a reference standard threshold was used, was it standard or pre-specified?
T8	Y	Was there an appropriate interval between index test and reference standard?
A1	PY	Were all enrolled participants included in the analysis?
A2	PY	Were missing data handled appropriately?
A3	Y	Does the unit of analysis match the ideal test accuracy trial?
A4	N	Were the estimates of sensitivity and specificity calculated appropriately?

Domain	Risk of bias	Applicability	Rationale	Source

Domain	Risk of bias	Applicability	Rationale	Source
Participants	High	High	Prospective diagnostic accuracy study of an AI caries-detection tool, paired with a before/after educational intervention study. Test set: 1,604 proximal surfaces across 400 bitewing images (200 images/1,142 surfaces read for enamel caries and 200 images read for dentinal caries by two examiners). Selection, enrichment, case-control construction, exclusions, or limited spectrum could materially bias accuracy.	PDF pp. 3, 7
Index test	High	Low	Index test: NR (described only as 'various machine and deep learning techniques'; Overjet Caries Assist, FDA-cleared 510(k) K212519). Model/threshold selection, non-independent evaluation, or access to reference-related information could materially bias performance.	PDF pp. 4, 6
Target condition	Low	Low	Reference standard: Consensus assessment by two calibrated examiners (OMR resident and dental student, calibrated by a board-certified oral and maxillofacial radiologist) reading the same bitewing images for enamel/dentinal caries. The reference procedure was judged sufficiently appropriate and independent for the study-defined target condition.	PDF pp. 3, 4
Analysis	High	Not applicable	Repeated surfaces/images and examiner readings were not incorporated as correlated observations in the reported accuracy calculations.	PDF pp. 2, 4
Overall	High	High	Overall risk of bias followed the prespecified rule: any High domain yields an overall High judgment; all Low yields Low; otherwise II. Overall applicability reflects the three applicability domains.	Derived from the domain judgments above

Source-status note: Full-text article reviewed. NI was used wherever the article did not report sufficient information.

S4.6.14. Chen 2026 (Q1)

Assessment element	Details
Design	Retrospective diagnostic accuracy study
Source-to-analysis flow	Source cohort/data set (Retrospective diagnostic accuracy study) → eligibility/selection as reported → development split(s), if applicable → selected independent/cross-validated test set: 10% patient-level split of 65 eligible patients: 157 axial slices for tooth detection and 2368 tooth-level samples for caries identification → diagnostic analysis at tooth level (post-processed: caries positive requires prediction in ≥3 consecutive axial slices) level. Any unreported exclusions or indeterminate/missing observations were coded NI in Phase 5.
Reported evaluation set	10% patient-level split of 65 eligible patients: 157 axial slices for tooth detection and 2368 tooth-level samples for caries identification
Analysis unit	tooth level (post-processed: caries positive requires prediction in ≥3 consecutive axial slices)
Selected estimate	Counts back-calculated from DenseNet169's reported sensitivity=0.5000, specificity=0.9828, PPV=0.8667, NPV=0.8976, which independently reproduce the reported overall accuracy (0.8944) and balanced accuracy (0.7414) exactly, supporting reconstruction validity. This is the tooth-level standalone carie
Numerical result	TP=13; FP=2; FN=13; TN=114; sensitivity=0.500; specificity=0.983
Selection rule	Primary or most clinically relevant test-set estimate matching the assigned synthesis question; alternative models/thresholds were not treated as independent studies.
Sources	Flow: PDF pp. 3, 10; estimate selection: PDF pp. 6, 10

Signal	Response	Official signaling question
P1	PN	Was a single-gate design used?
P2	N	Were participants prospectively enrolled?
P3	PN	Was a consecutive or random sample of participants included?
P4	N	Is the study group a representative sample of the intended-use population?
I1	N	Was the index test conducted and interpreted according to the recommended instructions?
I2	PY	Were the index test results interpreted without knowledge of the reference standard results?
I3	PN	Were the index test results interpreted with the same information as would be available when the test is used in practice?
I4	N	If an index test threshold was used, was it standard or pre-specified?
T1	PN	Does the reference standard adequately identify those with and without the target condition?
T2	Y	Was the target condition assessed in all participants?
T3	Y	Was the target condition assessed in the same way in all participants?
T4	Y	Did the reference standard avoid incorporating the index test?
T5	PY	Was the reference standard conducted and interpreted according to the recommended instructions?
T6	NI	Were the reference standard results interpreted without knowledge of the index test results?
T7	Y	If a reference standard threshold was used, was it standard or pre-specified?
T8	Y	Was there an appropriate interval between index test and reference standard?
A1	Y	Were all enrolled participants included in the analysis?
A2	Y	Were missing data handled appropriately?
A3	Y	Does the unit of analysis match the ideal test accuracy trial?
A4	Y	Were the estimates of sensitivity and specificity calculated appropriately?

Domain	Risk of bias	Applicability	Rationale	Source
Participants	High	High	Retrospective diagnostic accuracy study. Test set: 10% patient-level split of 65 eligible patients: 157 axial slices for tooth detection and 2368 tooth-level samples for caries identification. Selection, enrichment, case-control construction, exclusions, or limited spectrum could materially bias accuracy.	PDF pp. 3, 10

Domain	Risk of bias	Applicability	Rationale	Source
Index test	High	High	Index test: DenseNet169 (CNN, transfer learning); compared with MobileNet_V2 and ResNet50. Model/threshold selection, non-independent evaluation, or access to reference-related information could materially bias performance.	PDF pp. 3, 10
Target condition	High	Low	Reference standard: Consensus annotation by two dentists (>4 yrs experience) with adjudication by a senior endodontist (>10 yrs), based on explicit CBCT MPR criteria (enamel discontinuity with radiolucency, or radiolucency crossing EDJ into dentin). The reference procedure or its reliability was inadequate enough to risk systematic misclassification.	PDF pp. 3, 5
Analysis	Low	Not applicable	The held-out split was patient-level and the bootstrap analysis was aligned with the selected estimate; no material analysis-domain concern was identified for that estimate.	PDF pp. 6, 10
Overall	High	High	Overall risk of bias followed the prespecified rule: any High domain yields an overall High judgment; all Low yields Low; otherwise II. Overall applicability reflects the three applicability domains.	Derived from the domain judgments above

Source-status note: Full-text article reviewed. NI was used wherever the article did not report sufficient information.

S4.6.15. van Nistelrooij 2026 (Q1)

Assessment element	Details
Design	Retrospective diagnostic accuracy study
Source-to-analysis flow	Source cohort/data set (Retrospective diagnostic accuracy study) → eligibility/selection as reported → development split(s), if applicable → selected independent/cross-validated test set: 400 held-out radiographs from 242 held-out patients (200 OPGs, 150 bitewings, 50 PAs) → diagnostic analysis at Individual tooth (single-tooth image crop) level. Any unreported exclusions or indeterminate/missing observations were coded NI in Phase 5.
Reported evaluation set	400 held-out radiographs from 242 held-out patients (200 OPGs, 150 bitewings, 50 PAs)
Analysis unit	Individual tooth (single-tooth image crop)
Selected estimate	Extracted the model (standalone AI, 5-fold cross-validation mean) arm for 'Caries lesion' from Table 3/4, the authors' primary aggregate result across all radiograph modalities (support=688 diseased teeth = TP+FN). TP/FP/FN/TN reconstructed by combining reported precision, sensitivity, specificity a
Numerical result	TP=498; FP=221; FN=190; TN=6416; sensitivity=0.724; specificity=0.967
Selection rule	Primary or most clinically relevant test-set estimate matching the assigned synthesis question; alternative models/thresholds were not treated as independent studies.
Sources	Flow: PDF pp. 1, 5; estimate selection: PDF pp. 5, 8

Signal	Response	Official signaling question
P1	Y	Was a single-gate design used?
P2	N	Were participants prospectively enrolled?
P3	N	Was a consecutive or random sample of participants included?
P4	PY	Is the study group a representative sample of the intended-use population?
I1	Y	Was the index test conducted and interpreted according to the recommended instructions?
I2	PY	Were the index test results interpreted without knowledge of the reference standard results?
I3	Y	Were the index test results interpreted with the same information as would be available when the test is used in practice?
I4	NI	If an index test threshold was used, was it standard or pre-specified?
T1	PY	Does the reference standard adequately identify those with and without the target condition?
T2	Y	Was the target condition assessed in all participants?
T3	Y	Was the target condition assessed in the same way in all participants?
T4	Y	Did the reference standard avoid incorporating the index test?
T5	PY	Was the reference standard conducted and interpreted according to the recommended instructions?
T6	NI	Were the reference standard results interpreted without knowledge of the index test results?
T7	PY	If a reference standard threshold was used, was it standard or pre-specified?
T8	Y	Was there an appropriate interval between index test and reference standard?
A1	PY	Were all enrolled participants included in the analysis?
A2	PY	Were missing data handled appropriately?
A3	Y	Does the unit of analysis match the ideal test accuracy trial?
A4	N	Were the estimates of sensitivity and specificity calculated appropriately?

Domain	Risk of bias	Applicability	Rationale	Source
Participants	High	Low	Retrospective diagnostic accuracy study. Test set: 400 held-out radiographs from 242 held-out patients (200 OPGs, 150 bitewings, 50 PAs). Selection, enrichment, case-control construction, exclusions, or limited spectrum could materially bias accuracy.	PDF pp. 1, 5

Domain	Risk of bias	Applicability	Rationale	Source
Index test	II	Low	Index test: 3-stage deep learning pipeline: EfficientNetV2 modality classifier, RTMDet (Swin-B backbone) tooth detector/FDI numbering, EfficientNetV2-based binary dental-finding classifiers (single-tooth crops) for caries lesion presence. Threshold prespecification, blinding, or exact test-use instructions were incompletely reported.	PDF pp. 6, 9
Target condition	II	Low	Reference standard: Aggregated multi-clinician assessment (≥4 clinicians per held-out tooth) combined via Dawid-Skene probabilistic model; presence defined as aggregated reader score >0.5. Blinding, calibration, reliability, or threshold details were insufficiently reported; missing supplements were not imputed.	PDF pp. 2, 9
Analysis	High	Not applicable	Multiple radiographs/teeth per patient were analysed without an explicit clustered-data method.	PDF pp. 5, 8
Overall	High	Low	Overall risk of bias followed the prespecified rule: any High domain yields an overall High judgment; all Low yields Low; otherwise II. Overall applicability reflects the three applicability domains.	Derived from the domain judgments above

Source-status note: Full-text article reviewed. NI was used wherever the article did not report sufficient information.

S4.6.16. Giannakopoulos 2026 (Q1)

Assessment element	Details
Design	Retrospective diagnostic accuracy study
Source-to-analysis flow	Source cohort/data set (Retrospective diagnostic accuracy study) → eligibility/selection as reported → development split(s), if applicable → selected independent/cross-validated test set: 100 bitewing radiographs comprising 1540 approximal surfaces → diagnostic analysis at Tooth surface (approximal) level. Any unreported exclusions or indeterminate/missing observations were coded NI in Phase 5.
Reported evaluation set	100 bitewing radiographs comprising 1540 approximal surfaces
Analysis unit	Tooth surface (approximal)
Selected estimate	Table 2 is oriented reference-standard-in-rows; the initial extraction transposed FP/FN. Corrected assignment reproduces all four published operating characteristics exactly (Se 73.1%, Sp 94.3%, PPV 64.7%, NPV 96.1%, accuracy 91.6%). Primary arm extracted is 'any caries' (enamel+dentin combined), Ta
Numerical result	TP=141; FP=77; FN=52; TN=1270; sensitivity=0.731; specificity=0.943
Selection rule	Primary or most clinically relevant test-set estimate matching the assigned synthesis question; alternative models/thresholds were not treated as independent studies.
Sources	Flow: PDF pp. 3, 5; estimate selection: PDF pp. 5, 7

Signal	Response	Official signaling question
P1	Y	Was a single-gate design used?
P2	N	Were participants prospectively enrolled?
P3	PN	Was a consecutive or random sample of participants included?
P4	PN	Is the study group a representative sample of the intended-use population?
I1	Y	Was the index test conducted and interpreted according to the recommended instructions?
I2	Y	Were the index test results interpreted without knowledge of the reference standard results?
I3	Y	Were the index test results interpreted with the same information as would be available when the test is used in practice?
I4	Y	If an index test threshold was used, was it standard or pre-specified?
T1	Y	Does the reference standard adequately identify those with and without the target condition?
T2	Y	Was the target condition assessed in all participants?
T3	Y	Was the target condition assessed in the same way in all participants?
T4	Y	Did the reference standard avoid incorporating the index test?
T5	Y	Was the reference standard conducted and interpreted according to the recommended instructions?
T6	Y	Were the reference standard results interpreted without knowledge of the index test results?
T7	Y	If a reference standard threshold was used, was it standard or pre-specified?
T8	Y	Was there an appropriate interval between index test and reference standard?
A1	Y	Were all enrolled participants included in the analysis?
A2	Y	Were missing data handled appropriately?
A3	Y	Does the unit of analysis match the ideal test accuracy trial?
A4	Y	Were the estimates of sensitivity and specificity calculated appropriately?

Domain	Risk of bias	Applicability	Rationale	Source
Participants	High	High	Retrospective diagnostic accuracy study. Test set: 100 bitewing radiographs comprising 1540 approximal surfaces. Selection, enrichment, case-control construction, exclusions, or limited spectrum could materially bias accuracy.	PDF pp. 3, 5

Domain	Risk of bias	Applicability	Rationale	Source
Index test	Low	Low	Index test: Diagnocat™ (CNN-based commercial cloud AI system). A locked test evaluation and sufficiently prespecified interpretation were reported.	PDF pp. 2, 7
Target condition	Low	Low	Reference standard: Expert consensus (two calibrated examiners) using ICDAS radiographic criteria, binarized caries/no caries. The reference procedure was judged sufficiently appropriate and independent for the study-defined target condition.	PDF pp. 2, 5
Analysis	Low	Not applicable	The analysis used cluster-robust/bootstrap methods appropriate to surfaces nested within radiographs for the selected estimate.	PDF pp. 5, 7
Overall	High	High	Overall risk of bias followed the prespecified rule: any High domain yields an overall High judgment; all Low yields Low; otherwise II. Overall applicability reflects the three applicability domains.	Derived from the domain judgments above

Source-status note: Full-text article reviewed. NI was used wherever the article did not report sufficient information.

S4.6.17. Rahimi 2026 (Q1)

Assessment element	Details
Design	Retrospective dataset-benchmark diagnostic accuracy study
Source-to-analysis flow	Source cohort/data set (Retrospective dataset-benchmark diagnostic accuracy study) → eligibility/selection as reported → development split(s), if applicable → selected independent/cross-validated test set: Classification: 20 images held out as independent test set (4030 patches, 428 caries-positive); Segmentation: 40 samples held out as test set (from 170 total OPGs) → diagnostic analysis at Image patch (64x64 pixels) extracted from smile-zone region level. Any unreported exclusions or indeterminate/missing observations were coded NI in Phase 5.
Reported evaluation set	Classification: 20 images held out as independent test set (4030 patches, 428 caries-positive); Segmentation: 40 samples held out as test set (from 170 total OPGs)
Analysis unit	Image patch (64x64 pixels) extracted from smile-zone region
Selected estimate	Extracted the independent (held-out, non-cross-validated) test-set arm of the patch classifier, the authors' primary standalone-AI evaluation (5-fold CV results also reported but not used here). TP/FP/FN/TN reconstructed from reported Recall=0.857, Precision=0.318, total caries patches=428, and tota
Numerical result	TP=367; FP=787; FN=61; TN=2815; sensitivity=0.857; specificity=0.782
Selection rule	Primary or most clinically relevant test-set estimate matching the assigned synthesis question; alternative models/thresholds were not treated as independent studies.
Sources	Flow: PDF pp. 2, 15; estimate selection: PDF pp. 12, 13

Signal	Response	Official signaling question
P1	Y	Was a single-gate design used?
P2	N	Were participants prospectively enrolled?
P3	PY	Was a consecutive or random sample of participants included?
P4	PN	Is the study group a representative sample of the intended-use population?
I1	PY	Was the index test conducted and interpreted according to the recommended instructions?
I2	PY	Were the index test results interpreted without knowledge of the reference standard results?
I3	N	Were the index test results interpreted with the same information as would be available when the test is used in practice?
I4	NI	If an index test threshold was used, was it standard or pre-specified?
T1	PY	Does the reference standard adequately identify those with and without the target condition?
T2	Y	Was the target condition assessed in all participants?
T3	Y	Was the target condition assessed in the same way in all participants?
T4	Y	Did the reference standard avoid incorporating the index test?
T5	PY	Was the reference standard conducted and interpreted according to the recommended instructions?
T6	NI	Were the reference standard results interpreted without knowledge of the index test results?
T7	PY	If a reference standard threshold was used, was it standard or pre-specified?
T8	Y	Was there an appropriate interval between index test and reference standard?
A1	PY	Were all enrolled participants included in the analysis?
A2	PY	Were missing data handled appropriately?
A3	N	Does the unit of analysis match the ideal test accuracy trial?
A4	N	Were the estimates of sensitivity and specificity calculated appropriately?

Domain	Risk of bias	Applicability	Rationale	Source
Participants	High	Low	Retrospective dataset-benchmark diagnostic accuracy study. Test set: Classification: 20 images held out as independent test set (4030 patches, 428 caries-positive); Segmentation: 40 samples held out as test set (from 170 total OPGs). Selection, enrichment, case-control construction, exclusions, or limited spectrum could materially bias accuracy.	PDF pp. 2, 15

Domain	Risk of bias	Applicability	Rationale	Source
Index test	II	High	Index test: ResNet-18 (ImageNet-pretrained backbone) with custom global-average-pooling/dense/softmax classification head. Threshold prespecification, blinding, or exact test-use instructions were incompletely reported.	PDF pp. 9, 12
Target condition	II	Low	Reference standard: Expert-annotated caries polygons (proximal/occlusal) drawn by two mid-level annotators and validated by two dental specialists, used as ground-truth mask for patch labeling. Blinding, calibration, reliability, or threshold details were insufficiently reported; missing supplements were not imputed.	PDF pp. 4, 12
Analysis	High	Not applicable	Patch-level and segmentation observations did not match the clinical patient/image unit and dependence among patches was not addressed.	PDF pp. 12, 13
Overall	High	High	Overall risk of bias followed the prespecified rule: any High domain yields an overall High judgment; all Low yields Low; otherwise II. Overall applicability reflects the three applicability domains.	Derived from the domain judgments above

Source-status note: Full-text article reviewed. NI was used wherever the article did not report sufficient information.

S4.6.18. Aarti-Rajambigai 2026 (Q1)

Assessment element	Details
Design	Prospective observational diagnostic accuracy study
Source-to-analysis flow	Source cohort/data set (Prospective observational diagnostic accuracy study) → eligibility/selection as reported → development split(s), if applicable → selected independent/cross-validated test set: 320 patients (teledentistry submissions Jan-Sept 2025); caries subset analyzed as 187 active caries + 108 non-caries cases → diagnostic analysis at Patient submission (case-level, n=320 patients) level. Any unreported exclusions or indeterminate/missing observations were coded NI in Phase 5.
Reported evaluation set	320 patients (teledentistry submissions Jan-Sept 2025); caries subset analyzed as 187 active caries + 108 non-caries cases
Analysis unit	Patient submission (case-level, n=320 patients)
Selected estimate	Only the caries-detection arm was extracted per eligibility criteria (periodontitis and malocclusion arms reported but out of scope). FN and FP were derived algebraically from reported TP=187, TN=108, sensitivity=94.8%, specificity=92.1%; reconstructed total (314) is close to but not exactly 320, in
Numerical result	TP=187; FP=9; FN=10; TN=108; sensitivity=0.949; specificity=0.923
Selection rule	Primary or most clinically relevant test-set estimate matching the assigned synthesis question; alternative models/thresholds were not treated as independent studies.
Sources	Flow: PDF pp. 2, 3; estimate selection: PDF pp. 2, 3

Signal	Response	Official signaling question
P1	Y	Was a single-gate design used?
P2	Y	Were participants prospectively enrolled?
P3	NI	Was a consecutive or random sample of participants included?
P4	NI	Is the study group a representative sample of the intended-use population?
I1	NI	Was the index test conducted and interpreted according to the recommended instructions?
I2	Y	Were the index test results interpreted without knowledge of the reference standard results?
I3	Y	Were the index test results interpreted with the same information as would be available when the test is used in practice?
I4	NI	If an index test threshold was used, was it standard or pre-specified?
T1	PY	Does the reference standard adequately identify those with and without the target condition?
T2	Y	Was the target condition assessed in all participants?
T3	Y	Was the target condition assessed in the same way in all participants?
T4	Y	Did the reference standard avoid incorporating the index test?
T5	PY	Was the reference standard conducted and interpreted according to the recommended instructions?
T6	Y	Were the reference standard results interpreted without knowledge of the index test results?
T7	NI	If a reference standard threshold was used, was it standard or pre-specified?
T8	Y	Was there an appropriate interval between index test and reference standard?
A1	N	Were all enrolled participants included in the analysis?
A2	NI	Were missing data handled appropriately?
A3	Y	Does the unit of analysis match the ideal test accuracy trial?
A4	PN	Were the estimates of sensitivity and specificity calculated appropriately?

Domain	Risk of bias	Applicability	Rationale	Source
Participants	II	II	Prospective observational diagnostic accuracy study. Test set: 320 patients (teledentistry submissions Jan-Sept 2025); caries subset analyzed as 187 active caries + 108 non-caries cases. The full text did not provide enough information on sampling/representativeness for a definitive judgement.	PDF pp. 2, 3

Domain	Risk of bias	Applicability	Rationale	Source
Index test	II	Low	Index test: Convolutional neural network (image analysis) combined with a natural language processing pipeline (clinical text interpretation). Threshold prespecification, blinding, or exact test-use instructions were incompletely reported.	PDF pp. 2, 3
Target condition	II	Low	Reference standard: Independent consensus diagnosis by three board-certified dentists (blinded to AI output) based on intraoral photos, panoramic radiographs, and clinical history. Blinding, calibration, reliability, or threshold details were insufficiently reported; missing supplements were not imputed.	PDF pp. 2, 3
Analysis	High	Not applicable	Caries-specific denominators and reported totals did not fully reconcile, and handling of missing/indeterminate cases was not described.	PDF pp. 2, 3
Overall	High	II	Overall risk of bias followed the prespecified rule: any High domain yields an overall High judgment; all Low yields Low; otherwise II. Overall applicability reflects the three applicability domains.	Derived from the domain judgments above

Source-status note: Full-text article reviewed. NI was used wherever the article did not report sufficient information.

S4.6.19. Fadilah 2026 (Q1)

Assessment element	Details
Design	Cross-sectional diagnostic accuracy study with prospective data collection and field validation
Source-to-analysis flow	Source cohort/data set (Cross-sectional diagnostic accuracy study with prospective data collection and field validation) → eligibility/selection as reported → development split(s), if applicable → selected independent/cross-validated test set: 320 photographs (initial train/validation/test split of 3,221 images: 2,266/635/320); a separate field validation used 394 test data comprising 1,486 labeled instances across 320 new field-collected images (Table 4) → diagnostic analysis at ICDAS-labeled tooth surface/lesion instance, evaluated per ICDAS class (D1-D6) on new field-collected images level. Any unreported exclusions or indeterminate/missing observations were coded NI in Phase 5.
Reported evaluation set	320 photographs (initial train/validation/test split of 3,221 images: 2,266/635/320); a separate field validation used 394 test data comprising 1,486 labeled instances across 320 new field-collected images (Table 4)
Analysis unit	ICDAS-labeled tooth surface/lesion instance, evaluated per ICDAS class (D1-D6) on new field-collected images
Selected estimate	Paper reports six separate per-ICDAS-class (D1-D6) SEN/SPE/PPV/NPV values for AI vs. dentists (Table 5), with no single overall/pooled standalone-AI 2x2 designated as primary; D1 (any-caries threshold) row extracted as illustrative but not used for counts. Sample size is internally inconsistent (tex
Numerical result	AI, ICDAS D1 field-validation row: sensitivity=0.9080; specificity=0.9017.
Selection rule	Primary or most clinically relevant test-set estimate matching the assigned synthesis question; alternative models/thresholds were not treated as independent studies.
Sources	Flow: PDF pp. 3, 11; estimate selection: PDF pp. 10, 11

Signal	Response	Official signaling question
P1	Y	Was a single-gate design used?
P2	Y	Were participants prospectively enrolled?
P3	N	Was a consecutive or random sample of participants included?
P4	PY	Is the study group a representative sample of the intended-use population?
I1	PY	Was the index test conducted and interpreted according to the recommended instructions?
I2	NI	Were the index test results interpreted without knowledge of the reference standard results?
I3	PY	Were the index test results interpreted with the same information as would be available when the test is used in practice?
I4	NI	If an index test threshold was used, was it standard or pre-specified?
T1	PY	Does the reference standard adequately identify those with and without the target condition?
T2	Y	Was the target condition assessed in all participants?
T3	Y	Was the target condition assessed in the same way in all participants?
T4	Y	Did the reference standard avoid incorporating the index test?
T5	PY	Was the reference standard conducted and interpreted according to the recommended instructions?
T6	NI	Were the reference standard results interpreted without knowledge of the index test results?
T7	Y	If a reference standard threshold was used, was it standard or pre-specified?
T8	Y	Was there an appropriate interval between index test and reference standard?
A1	PN	Were all enrolled participants included in the analysis?
A2	NI	Were missing data handled appropriately?
A3	Y	Does the unit of analysis match the ideal test accuracy trial?
A4	N	Were the estimates of sensitivity and specificity calculated appropriately?

Domain	Risk of bias	Applicability	Rationale	Source

Domain	Risk of bias	Applicability	Rationale	Source
Participants	High	Low	Cross-sectional diagnostic accuracy study with prospective data collection and field validation. Test set: 320 photographs (initial train/validation/test split of 3,221 images: 2,266/635/320); a separate field validation used 394 test data comprising 1,486 labeled instances across 320 new field-collected images (Table 4). Selection, enrichment, case-control construction, exclusions, or limited spectrum could materially bias accuracy.	PDF pp. 3, 11
Index test	II	Low	Index test: YOLOv8x (CSPDarknet53 backbone, PANet neck, YOLO detection head; HI Bogi app). Threshold prespecification, blinding, or exact test-use instructions were incompletely reported.	PDF pp. 5, 7
Target condition	II	Low	Reference standard: ICDAS (D0-D6) visual-tactile classification, annotated by calibrated dentists and confirmed only when two independent ICDAS experts agreed. Blinding, calibration, reliability, or threshold details were insufficiently reported; missing supplements were not imputed.	PDF pp. 3, 7
Analysis	High	Not applicable	Field-validation counts and class-specific denominators were not fully reconciled; repeated instances within images/children were not modelled.	PDF pp. 10, 11
Overall	High	Low	Overall risk of bias followed the prespecified rule: any High domain yields an overall High judgment; all Low yields Low; otherwise II. Overall applicability reflects the three applicability domains.	Derived from the domain judgments above

Source-status note: Full-text article reviewed. NI was used wherever the article did not report sufficient information.

S4.6.20. Wang 2026 (Q1)

Assessment element	Details
Design	Retrospective diagnostic accuracy study
Source-to-analysis flow	Source cohort/data set (Retrospective diagnostic accuracy study) → eligibility/selection as reported → development split(s), if applicable → selected independent/cross-validated test set: 579 panoramic images (2,119 annotated lesions), 15% of the total dataset → diagnostic analysis at Individual carious lesion (bounding-box detection with severity grade D1-D4); some analyses also reported at image/patient level level. Any unreported exclusions or indeterminate/missing observations were coded NI in Phase 5.
Reported evaluation set	579 panoramic images (2,119 annotated lesions), 15% of the total dataset
Analysis unit	Individual carious lesion (bounding-box detection with severity grade D1-D4); some analyses also reported at image/patient level
Selected estimate	Primary standalone-AI arm extracted is the proposed Transformer model's overall test-set performance vs expert consensus (the paper's headline comparison), not the six baseline CNN/DETR/Swin comparator arms in Table 4 or the AI-assisted-clinician framing mentioned only in Discussion. A full 2x2 coul
Numerical result	Proposed Transformer, overall independent test set: sensitivity=0.884; specificity=0.917.
Selection rule	Primary or most clinically relevant test-set estimate matching the assigned synthesis question; alternative models/thresholds were not treated as independent studies.
Sources	Flow: PDF pp. 2, 5; estimate selection: PDF pp. 3, 7

Signal	Response	Official signaling question
P1	PY	Was a single-gate design used?
P2	N	Were participants prospectively enrolled?
P3	NI	Was a consecutive or random sample of participants included?
P4	PY	Is the study group a representative sample of the intended-use population?
I1	PN	Was the index test conducted and interpreted according to the recommended instructions?
I2	PY	Were the index test results interpreted without knowledge of the reference standard results?
I3	Y	Were the index test results interpreted with the same information as would be available when the test is used in practice?
I4	N	If an index test threshold was used, was it standard or pre-specified?
T1	Y	Does the reference standard adequately identify those with and without the target condition?
T2	Y	Was the target condition assessed in all participants?
T3	Y	Was the target condition assessed in the same way in all participants?
T4	Y	Did the reference standard avoid incorporating the index test?
T5	Y	Was the reference standard conducted and interpreted according to the recommended instructions?
T6	PY	Were the reference standard results interpreted without knowledge of the index test results?
T7	Y	If a reference standard threshold was used, was it standard or pre-specified?
T8	Y	Was there an appropriate interval between index test and reference standard?
A1	PY	Were all enrolled participants included in the analysis?
A2	PY	Were missing data handled appropriately?
A3	Y	Does the unit of analysis match the ideal test accuracy trial?
A4	N	Were the estimates of sensitivity and specificity calculated appropriately?

Domain	Risk of bias	Applicability	Rationale	Source
Participants	High	Low	Retrospective diagnostic accuracy study. Test set: 579 panoramic images (2,119 annotated lesions), 15% of the total dataset. Selection, enrichment, case-control construction, exclusions, or limited spectrum could materially bias accuracy.	PDF pp. 2, 5

Domain	Risk of bias	Applicability	Rationale	Source
Index test	High	Low	Index test: Domain-adapted hierarchical/pyramid Vision Transformer encoder (12 layers, 12 heads, 768-dim) with multi-scale feature fusion, spatially-aware attention, enhanced 2D positional encoding, feature enhancement module, and FPN-based detection/severity-grading decoder. Model/threshold selection, non-independent evaluation, or access to reference-related information could materially bias performance.	PDF pp. 5, 7
Target condition	Low	Low	Reference standard: Expert consensus annotation: bounding-box lesion labeling and D1-D4 severity grading by three experienced dentists (10+ yrs) with senior-endodontist adjudication of disagreements (visual-radiographic reference, not histological). The reference procedure was judged sufficiently appropriate and independent for the study-defined target condition.	PDF pp. 7, 8
Analysis	High	Not applicable	Lesions nested within panoramic images/patients were analysed as independent observations.	PDF pp. 3, 7
Overall	High	Low	Overall risk of bias followed the prespecified rule: any High domain yields an overall High judgment; all Low yields Low; otherwise II. Overall applicability reflects the three applicability domains.	Derived from the domain judgments above

Source-status note: Full-text article reviewed. NI was used wherever the article did not report sufficient information.

S4.6.21. Liu 2026 (Q1)

Assessment element	Details
Design	Prospective cross-sectional diagnostic accuracy study
Source-to-analysis flow	Source cohort/data set (Prospective cross-sectional diagnostic accuracy study) → eligibility/selection as reported → development split(s), if applicable → selected independent/cross-validated test set: 20 percent of participants reserved as an independent test set, drawn from 524 enrolled participants and 7671 images; exact test-set n not stated → diagnostic analysis at Individual tooth surface images (occlusal and smooth), single-tooth crops level. Any unreported exclusions or indeterminate/missing observations were coded NI in Phase 5.
Reported evaluation set	20 percent of participants reserved as an independent test set, drawn from 524 enrolled participants and 7671 images; exact test-set n not stated
Analysis unit	Individual tooth surface images (occlusal and smooth), single-tooth crops
Selected estimate	Study reports a three-class classification (caries-free/early/moderate-severe) per tooth surface (occlusal, smooth) rather than binary diagnostic 2x2 outcomes, so TP/FP/FN/TN cannot be reconstructed without assuming a specific positive-class threshold and knowing exact class sample sizes (not report
Numerical result	Reported numerical estimate described in the selected-estimate field; no complete 2×2 table was reconstructed.
Selection rule	Primary or most clinically relevant test-set estimate matching the assigned synthesis question; alternative models/thresholds were not treated as independent studies.
Sources	Flow: PDF pp. 7, 11; estimate selection: PDF pp. 4, 7

Signal	Response	Official signaling question
P1	Y	Was a single-gate design used?
P2	Y	Were participants prospectively enrolled?
P3	PY	Was a consecutive or random sample of participants included?
P4	Y	Is the study group a representative sample of the intended-use population?
I1	Y	Was the index test conducted and interpreted according to the recommended instructions?
I2	Y	Were the index test results interpreted without knowledge of the reference standard results?
I3	Y	Were the index test results interpreted with the same information as would be available when the test is used in practice?
I4	Y	If an index test threshold was used, was it standard or pre-specified?
T1	PY	Does the reference standard adequately identify those with and without the target condition?
T2	Y	Was the target condition assessed in all participants?
T3	Y	Was the target condition assessed in the same way in all participants?
T4	Y	Did the reference standard avoid incorporating the index test?
T5	Y	Was the reference standard conducted and interpreted according to the recommended instructions?
T6	NI	Were the reference standard results interpreted without knowledge of the index test results?
T7	Y	If a reference standard threshold was used, was it standard or pre-specified?
T8	Y	Was there an appropriate interval between index test and reference standard?
A1	Y	Were all enrolled participants included in the analysis?
A2	PY	Were missing data handled appropriately?
A3	Y	Does the unit of analysis match the ideal test accuracy trial?
A4	N	Were the estimates of sensitivity and specificity calculated appropriately?

Domain	Risk of bias	Applicability	Rationale	Source
Participants	Low	Low	Prospective cross-sectional diagnostic accuracy study. Test set: 20 percent of participants reserved as an independent test set, drawn from 524 enrolled participants and 7671 images; exact test-set n not stated. Sampling and spectrum were sufficiently aligned with the review question for this domain.	PDF pp. 7, 11

Domain	Risk of bias	Applicability	Rationale	Source
Index test	Low	Low	Index test: YOLOv8 (tooth detection) + TResNet (image classification, unimodal); TResNet features fused with MLP and one-hot questionnaire data (multimodal). A locked test evaluation and sufficiently prespecified interpretation were reported.	PDF pp. 5, 10
Target condition	II	Low	Reference standard: Clinical examination by experienced examiner using ICDAS-II criteria (0=caries-free, 1-2=early caries, ≥3=moderate-to-severe caries). Blinding, calibration, reliability, or threshold details were insufficiently reported; missing supplements were not imputed.	PDF pp. 3, 4
Analysis	High	Not applicable	Multiple images/teeth per participant were not handled as clustered observations in the selected accuracy estimate.	PDF pp. 4, 7
Overall	High	Low	Overall risk of bias followed the prespecified rule: any High domain yields an overall High judgment; all Low yields Low; otherwise II. Overall applicability reflects the three applicability domains.	Derived from the domain judgments above

Source-status note: Full-text article reviewed. NI was used wherever the article did not report sufficient information.

S4.6.22. Parekh 2026 (Q1)

Assessment element	Details
Design	Retrospective diagnostic accuracy study
Source-to-analysis flow	Source cohort/data set (Retrospective diagnostic accuracy study) → eligibility/selection as reported → development split(s), if applicable → selected independent/cross-validated test set: 407 bitewing radiographs from 315 adult patients, yielding 2829 tooth-level observations → diagnostic analysis at Individual tooth-level observations (2,829 total annotated; 2657-2820 retained per finding after excluding teeth with observer disagreement) level. Any unreported exclusions or indeterminate/missing observations were coded NI in Phase 5.
Reported evaluation set	407 bitewing radiographs from 315 adult patients, yielding 2829 tooth-level observations
Analysis unit	Individual tooth-level observations (2,829 total annotated; 2657-2820 retained per finding after excluding teeth with observer disagreement)
Selected estimate	Caries is one of four arms (caries, restorations, prosthesis, implant); caries extracted as the target condition for this review. Only sensitivity/specificity are reported per finding, with a stated range of 2657-2820 tooth-level observations post-filtration across all four findings combined, but ex
Numerical result	Caries arm after human-consensus filtration: sensitivity=0.972; specificity=0.842.
Selection rule	Primary or most clinically relevant test-set estimate matching the assigned synthesis question; alternative models/thresholds were not treated as independent studies.
Sources	Flow: PDF pp. 1, 3; estimate selection: PDF pp. 1, 4

Signal	Response	Official signaling question
P1	Y	Was a single-gate design used?
P2	N	Were participants prospectively enrolled?
P3	N	Was a consecutive or random sample of participants included?
P4	N	Is the study group a representative sample of the intended-use population?
I1	PY	Was the index test conducted and interpreted according to the recommended instructions?
I2	PY	Were the index test results interpreted without knowledge of the reference standard results?
I3	Y	Were the index test results interpreted with the same information as would be available when the test is used in practice?
I4	NI	If an index test threshold was used, was it standard or pre-specified?
T1	Y	Does the reference standard adequately identify those with and without the target condition?
T2	Y	Was the target condition assessed in all participants?
T3	Y	Was the target condition assessed in the same way in all participants?
T4	Y	Did the reference standard avoid incorporating the index test?
T5	Y	Was the reference standard conducted and interpreted according to the recommended instructions?
T6	Y	Were the reference standard results interpreted without knowledge of the index test results?
T7	Y	If a reference standard threshold was used, was it standard or pre-specified?
T8	Y	Was there an appropriate interval between index test and reference standard?
A1	N	Were all enrolled participants included in the analysis?
A2	N	Were missing data handled appropriately?
A3	Y	Does the unit of analysis match the ideal test accuracy trial?
A4	N	Were the estimates of sensitivity and specificity calculated appropriately?

Domain	Risk of bias	Applicability	Rationale	Source
Participants	High	High	Retrospective diagnostic accuracy study. Test set: 407 bitewing radiographs from 315 adult patients, yielding 2829 tooth-level observations. Selection, enrichment, case-control construction, exclusions, or limited spectrum could materially bias accuracy.	PDF pp. 1, 3

Domain	Risk of bias	Applicability	Rationale	Source
Index test	II	Low	Index test: Convolutional neural network (VELMENI Inc. proprietary CNN; exact architecture/training parameters undisclosed as confidential IP). Threshold prespecification, blinding, or exact test-use instructions were incompletely reported.	PDF pp. 3, 6
Target condition	Low	Low	Reference standard: Consensus (agreement) of two oral and maxillofacial radiologists' tooth-level annotations for caries presence/absence. The reference procedure was judged sufficiently appropriate and independent for the study-defined target condition.	PDF pp. 1, 5
Analysis	High	Not applicable	Substantial exclusions and tooth-level observations nested within patients were not addressed in the accuracy analysis.	PDF pp. 1, 4
Overall	High	High	Overall risk of bias followed the prespecified rule: any High domain yields an overall High judgment; all Low yields Low; otherwise II. Overall applicability reflects the three applicability domains.	Derived from the domain judgments above

Source-status note: Full-text article reviewed. NI was used wherever the article did not report sufficient information.

S4.6.23. Bicengil 2026 (Q1)

Assessment element	Details
Design	Retrospective diagnostic accuracy study
Source-to-analysis flow	Source cohort/data set (Retrospective diagnostic accuracy study) → eligibility/selection as reported → development split(s), if applicable → selected independent/cross-validated test set: 152 panoramic radiographs (10% of the 1526-image dataset) used as the independent AI test set; a separate randomly selected subset of 100 radiographs was used for AI-vs-human observer comparison → diagnostic analysis at Lesion-level detections (bounding box, IoU≥0.50) per caries type (approximal, occlusal, buccal) in primary molars level. Any unreported exclusions or indeterminate/missing observations were coded NI in Phase 5.
Reported evaluation set	152 panoramic radiographs (10% of the 1526-image dataset) used as the independent AI test set; a separate randomly selected subset of 100 radiographs was used for AI-vs-human observer comparison
Analysis unit	Lesion-level detections (bounding box, IoU≥0.50) per caries type (approximal, occlusal, buccal) in primary molars
Selected estimate	Multiple lesion-type arms (approximal, occlusal, buccal) and an overall pooled arm reported; extracted the overall pooled standalone-AI arm as primary since authors present it as the summary comparison (Table 2/Section 3.4). TP=477 stated directly; FP and FN back-calculated from precision=TP/(TP+FP)
Numerical result	Reported numerical estimate described in the selected-estimate field; no complete 2×2 table was reconstructed.
Selection rule	Primary or most clinically relevant test-set estimate matching the assigned synthesis question; alternative models/thresholds were not treated as independent studies.
Sources	Flow: PDF pp. 3, 13; estimate selection: PDF pp. 11, 12

Signal	Response	Official signaling question
P1	Y	Was a single-gate design used?
P2	N	Were participants prospectively enrolled?
P3	NI	Was a consecutive or random sample of participants included?
P4	PY	Is the study group a representative sample of the intended-use population?
I1	Y	Was the index test conducted and interpreted according to the recommended instructions?
I2	Y	Were the index test results interpreted without knowledge of the reference standard results?
I3	Y	Were the index test results interpreted with the same information as would be available when the test is used in practice?
I4	Y	If an index test threshold was used, was it standard or pre-specified?
T1	Y	Does the reference standard adequately identify those with and without the target condition?
T2	Y	Was the target condition assessed in all participants?
T3	Y	Was the target condition assessed in the same way in all participants?
T4	Y	Did the reference standard avoid incorporating the index test?
T5	Y	Was the reference standard conducted and interpreted according to the recommended instructions?
T6	Y	Were the reference standard results interpreted without knowledge of the index test results?
T7	Y	If a reference standard threshold was used, was it standard or pre-specified?
T8	Y	Was there an appropriate interval between index test and reference standard?
A1	PY	Were all enrolled participants included in the analysis?
A2	PY	Were missing data handled appropriately?
A3	Y	Does the unit of analysis match the ideal test accuracy trial?
A4	N	Were the estimates of sensitivity and specificity calculated appropriately?

Domain	Risk of bias	Applicability	Rationale	Source

Domain	Risk of bias	Applicability	Rationale	Source
Participants	High	Low	Retrospective diagnostic accuracy study. Test set: 152 panoramic radiographs (10% of the 1526-image dataset) used as the independent AI test set; a separate randomly selected subset of 100 radiographs was used for AI-vs-human observer comparison. Selection, enrichment, case-control construction, exclusions, or limited spectrum could materially bias accuracy.	PDF pp. 3, 13
Index test	Low	Low	Index test: YOLOv8x object-detection model (CranioCatch platform). A locked test evaluation and sufficiently prespecified interpretation were reported.	PDF pp. 3, 8
Target condition	Low	Low	Reference standard: Expert-consensus annotation by paediatric dentist (>10 yrs experience) and oral/maxillofacial radiologist (5 yrs experience) on 100-image test subset, with intra-observer kappa=0.83. The reference procedure was judged sufficiently appropriate and independent for the study-defined target condition.	PDF pp. 4, 5
Analysis	High	Not applicable	Tooth/lesion-level observations nested within panoramic radiographs were not modelled as correlated.	PDF pp. 11, 12
Overall	High	Low	Overall risk of bias followed the prespecified rule: any High domain yields an overall High judgment; all Low yields Low; otherwise II. Overall applicability reflects the three applicability domains.	Derived from the domain judgments above

Source-status note: Full-text article reviewed. NI was used wherever the article did not report sufficient information.

S4.6.24. Abdelaziz 2026 (Q1)

Assessment element	Details
Design	Prospective cross-sectional diagnostic accuracy study
Source-to-analysis flow	Source cohort/data set (Prospective cross-sectional diagnostic accuracy study) → eligibility/selection as reported → development split(s), if applicable → selected independent/cross-validated test set: 306 photographs from 34 patients (independent validation set; separate from the developer's 1,255-image training/validation/test split) → diagnostic analysis at Individual teeth/clinical features (caries/secondary caries lesions) within photographs, not per-patient level. Any unreported exclusions or indeterminate/missing observations were coded NI in Phase 5.
Reported evaluation set	306 photographs from 34 patients (independent validation set; separate from the developer's 1,255-image training/validation/test split)
Analysis unit	Individual teeth/clinical features (caries/secondary caries lesions) within photographs, not per-patient
Selected estimate	Extracted the 'Caries/secondary caries (C)' arm as primary caries outcome (ICDAS reference); a second caries-related arm 'Pit and fissure caries/stain (P)' is also reported but not selected as primary. Only percentages with CIs and prevalence percentages are given in Table 1 (no raw denominators per
Numerical result	Caries/secondary-caries arm: sensitivity=0.3303; specificity=0.9571.
Selection rule	Primary or most clinically relevant test-set estimate matching the assigned synthesis question; alternative models/thresholds were not treated as independent studies.
Sources	Flow: PDF pp. 2, 8; estimate selection: PDF pp. 4, 7

Signal	Response	Official signaling question
P1	Y	Was a single-gate design used?
P2	Y	Were participants prospectively enrolled?
P3	NI	Was a consecutive or random sample of participants included?
P4	PN	Is the study group a representative sample of the intended-use population?
I1	Y	Was the index test conducted and interpreted according to the recommended instructions?
I2	Y	Were the index test results interpreted without knowledge of the reference standard results?
I3	PN	Were the index test results interpreted with the same information as would be available when the test is used in practice?
I4	PY	If an index test threshold was used, was it standard or pre-specified?
T1	PY	Does the reference standard adequately identify those with and without the target condition?
T2	Y	Was the target condition assessed in all participants?
T3	Y	Was the target condition assessed in the same way in all participants?
T4	Y	Did the reference standard avoid incorporating the index test?
T5	Y	Was the reference standard conducted and interpreted according to the recommended instructions?
T6	NI	Were the reference standard results interpreted without knowledge of the index test results?
T7	Y	If a reference standard threshold was used, was it standard or pre-specified?
T8	Y	Was there an appropriate interval between index test and reference standard?
A1	PY	Were all enrolled participants included in the analysis?
A2	PY	Were missing data handled appropriately?
A3	Y	Does the unit of analysis match the ideal test accuracy trial?
A4	N	Were the estimates of sensitivity and specificity calculated appropriately?

Domain	Risk of bias	Applicability	Rationale	Source
Participants	High	Low	Prospective cross-sectional diagnostic accuracy study. Test set: 306 photographs from 34 patients (independent validation set; separate from the developer's 1,255-image training/validation/test split). Selection, enrichment, case-control construction, exclusions, or limited spectrum could materially bias accuracy.	PDF pp. 2, 8

Domain	Risk of bias	Applicability	Rationale	Source
Index test	Low	High	Index test: Mask R-CNN with Swin Transformer backbone (instance segmentation + object detection). A locked test evaluation and sufficiently prespecified interpretation were reported.	PDF pp. 7, 9
Target condition	II	Low	Reference standard: Examiner consensus (2 examiners + 3rd for adjudication) using ICDAS for caries assessment. Blinding, calibration, reliability, or threshold details were insufficiently reported; missing supplements were not imputed.	PDF pp. 3, 8
Analysis	High	Not applicable	Repeated photographs/teeth within only 34 patients were analysed without a patient-level clustered-data method.	PDF pp. 4, 7
Overall	High	High	Overall risk of bias followed the prespecified rule: any High domain yields an overall High judgment; all Low yields Low; otherwise II. Overall applicability reflects the three applicability domains.	Derived from the domain judgments above

Source-status note: Full-text article reviewed. NI was used wherever the article did not report sufficient information.

S4.6.25. Kang 2026 (Q1)

Assessment element	Details
Design	Pilot prospective diagnostic accuracy study
Source-to-analysis flow	Source cohort/data set (Pilot prospective diagnostic accuracy study) → eligibility/selection as reported → development split(s), if applicable → selected independent/cross-validated test set: 10 percent of 70 participants at the patient level, corresponding to 494 tooth-level instances (five-class scheme) from the 420 smartphone images → diagnostic analysis at Tooth (tooth-level aggregation of predictions across images; annotated at tooth-surface visual instance level for training) level. Any unreported exclusions or indeterminate/missing observations were coded NI in Phase 5.
Reported evaluation set	10 percent of 70 participants at the patient level, corresponding to 494 tooth-level instances (five-class scheme) from the 420 smartphone images
Analysis unit	Tooth (tooth-level aggregation of predictions across images; annotated at tooth-surface visual instance level for training)
Selected estimate	Three arms reported (binary, four-class, five-class classification schemes) using the same YOLOv6-L6 model; extracted the binary classification arm as primary since it is the simplest caries-vs-no-caries standalone AI comparison and authors present it first in Table 5. Raw TP/FP/FN/TN confusion matr
Numerical result	Prespecified five-class scheme: sensitivity=0.86; specificity=0.9845; accuracy=0.936.
Selection rule	Primary or most clinically relevant test-set estimate matching the assigned synthesis question; alternative models/thresholds were not treated as independent studies.
Sources	Flow: PDF pp. 3, 4; estimate selection: PDF pp. 2, 10

Signal	Response	Official signaling question
P1	Y	Was a single-gate design used?
P2	Y	Were participants prospectively enrolled?
P3	N	Was a consecutive or random sample of participants included?
P4	N	Is the study group a representative sample of the intended-use population?
I1	PY	Was the index test conducted and interpreted according to the recommended instructions?
I2	PY	Were the index test results interpreted without knowledge of the reference standard results?
I3	PN	Were the index test results interpreted with the same information as would be available when the test is used in practice?
I4	N	If an index test threshold was used, was it standard or pre-specified?
T1	PN	Does the reference standard adequately identify those with and without the target condition?
T2	Y	Was the target condition assessed in all participants?
T3	Y	Was the target condition assessed in the same way in all participants?
T4	Y	Did the reference standard avoid incorporating the index test?
T5	PY	Was the reference standard conducted and interpreted according to the recommended instructions?
T6	NI	Were the reference standard results interpreted without knowledge of the index test results?
T7	Y	If a reference standard threshold was used, was it standard or pre-specified?
T8	Y	Was there an appropriate interval between index test and reference standard?
A1	PY	Were all enrolled participants included in the analysis?
A2	PY	Were missing data handled appropriately?
A3	Y	Does the unit of analysis match the ideal test accuracy trial?
A4	N	Were the estimates of sensitivity and specificity calculated appropriately?

Domain	Risk of bias	Applicability	Rationale	Source

Domain	Risk of bias	Applicability	Rationale	Source
Participants	High	High	Pilot prospective diagnostic accuracy study. Test set: 10 percent of 70 participants at the patient level, corresponding to 494 tooth-level instances (five-class scheme) from the 420 smartphone images. Selection, enrichment, case-control construction, exclusions, or limited spectrum could materially bias accuracy.	PDF pp. 3, 4
Index test	High	High	Index test: YOLOv6-L6 (EfficientRep backbone, Rep-PAN neck, decoupled detection head; CNN-based object detector). Model/threshold selection, non-independent evaluation, or access to reference-related information could materially bias performance.	PDF pp. 4, 5
Target condition	High	II	Reference standard: Expert consensus visual annotation by 4 calibrated dentists using ICDAS-II and ADA CCS criteria on smartphone photographs (no independent clinical/radiographic/histological reference). The reference procedure or its reliability was inadequate enough to risk systematic misclassification.	PDF pp. 2, 4
Analysis	High	Not applicable	Although the split was patient-level, tooth-level accuracy and threshold optimisation did not adequately propagate clustering/selection into uncertainty.	PDF pp. 2, 10
Overall	High	High	Overall risk of bias followed the prespecified rule: any High domain yields an overall High judgment; all Low yields Low; otherwise II. Overall applicability reflects the three applicability domains.	Derived from the domain judgments above

Source-status note: Full-text article reviewed. NI was used wherever the article did not report sufficient information.

S4.6.26. Ren 2026 (Q1)

Assessment element	Details
Design	Retrospective diagnostic accuracy study (algorithm development/validation using public image datasets)
Source-to-analysis flow	Source cohort/data set (Retrospective diagnostic accuracy study (algorithm development/validation using public image datasets)) → eligibility/selection as reported → development split(s), if applicable → selected independent/cross-validated test set: 20% independent frozen test set from 639 intra-oral photographs (481 caries, 158 healthy) and 456 radiographs (268 caries, 188 healthy); 5-fold cross-validation on remaining 80% development set, repeated 10 times → diagnostic analysis at Individual tooth images (image-level, not patient-level; 639 oral photographs, 456 radiographs) level. Any unreported exclusions or indeterminate/missing observations were coded NI in Phase 5.
Reported evaluation set	20% independent frozen test set from 639 intra-oral photographs (481 caries, 158 healthy) and 456 radiographs (268 caries, 188 healthy); 5-fold cross-validation on remaining 80% development set, repeated 10 times
Analysis unit	Individual tooth images (image-level, not patient-level; 639 oral photographs, 456 radiographs)
Selected estimate	Only precision/recall/accuracy/F1/AUC per fold on a held-out 20% test set are reported (mean ± SD across 5-fold CV repeated 10 times); no raw TP/FP/FN/TN counts or specificity are given, and class sizes per fold/test split are not stated, so a 2x2 table cannot be reconstructed. Extracted the primary
Numerical result	Reported numerical estimate described in the selected-estimate field; no complete 2×2 table was reconstructed.
Selection rule	Primary or most clinically relevant test-set estimate matching the assigned synthesis question; alternative models/thresholds were not treated as independent studies.
Sources	Flow: PDF pp. 16, 20; estimate selection: PDF pp. 3, 16

Signal	Response	Official signaling question
P1	N	Was a single-gate design used?
P2	N	Were participants prospectively enrolled?
P3	NI	Was a consecutive or random sample of participants included?
P4	N	Is the study group a representative sample of the intended-use population?
I1	PY	Was the index test conducted and interpreted according to the recommended instructions?
I2	NI	Were the index test results interpreted without knowledge of the reference standard results?
I3	PN	Were the index test results interpreted with the same information as would be available when the test is used in practice?
I4	NI	If an index test threshold was used, was it standard or pre-specified?
T1	Y	Does the reference standard adequately identify those with and without the target condition?
T2	Y	Was the target condition assessed in all participants?
T3	Y	Was the target condition assessed in the same way in all participants?
T4	Y	Did the reference standard avoid incorporating the index test?
T5	Y	Was the reference standard conducted and interpreted according to the recommended instructions?
T6	PY	Were the reference standard results interpreted without knowledge of the index test results?
T7	Y	If a reference standard threshold was used, was it standard or pre-specified?
T8	Y	Was there an appropriate interval between index test and reference standard?
A1	PN	Were all enrolled participants included in the analysis?
A2	NI	Were missing data handled appropriately?
A3	PN	Does the unit of analysis match the ideal test accuracy trial?
A4	N	Were the estimates of sensitivity and specificity calculated appropriately?

Domain	Risk of bias	Applicability	Rationale	Source

Domain	Risk of bias	Applicability	Rationale	Source
Participants	High	High	Retrospective diagnostic accuracy study (algorithm development/validation using public image datasets). Test set: 20% independent frozen test set from 639 intra-oral photographs (481 caries, 158 healthy) and 456 radiographs (268 caries, 188 healthy); 5-fold cross-validation on remaining 80% development set, repeated 10 times. Selection, enrichment, case-control construction, exclusions, or limited spectrum could materially bias accuracy.	PDF pp. 16, 20
Index test	II	High	Index test: DBAttNet (dual-branch attention-augmented CNN, ResNet50 backbone with modality-specific attention: IRAA for photographs, CFA for radiographs, plus shared CBAM). Threshold prespecification, blinding, or exact test-use instructions were incompletely reported.	PDF pp. 13, 18
Target condition	Low	II	Reference standard: Consensus annotation by two licensed dentists (caries vs. healthy), with disagreements resolved by discussion or third senior dentist adjudication. The reference procedure was judged sufficiently appropriate and independent for the study-defined target condition.	PDF pp. 12, 22
Analysis	High	Not applicable	Healthy/caries image selection, exclusions and image-level observations did not support a complete patient-level intention-to-analyse estimate.	PDF pp. 3, 16
Overall	High	High	Overall risk of bias followed the prespecified rule: any High domain yields an overall High judgment; all Low yields Low; otherwise II. Overall applicability reflects the three applicability domains.	Derived from the domain judgments above

Source-status note: Full-text article reviewed. NI was used wherever the article did not report sufficient information.

S4.6.27. Mertens 2021 (Q2)

Assessment element	Details
Design	Cluster-randomized cross-over controlled trial evaluating the effect of an AI-based decision-support system (dentalXrai Pro) on proximal caries detection by dentists using bitewing radiographs.
Source-to-analysis flow	Source cohort/data set (Cluster-randomized cross-over controlled trial evaluating the effect of an AI-based decision-support system (dentalXrai Pro) on proximal caries detection by dentists using bitewing radiographs.) → eligibility/selection as reported → development split(s), if applicable → selected independent/cross-validated test set: 140 radiographs (used across dentists; not a classical test set) → diagnostic analysis at Proximal tooth surface. level. Any unreported exclusions or indeterminate/missing observations were coded NI in Phase 5.
Reported evaluation set	140 radiographs (used across dentists; not a classical test set)
Analysis unit	Proximal tooth surface.
Selected estimate	Design-effect adjusted reader data; no complete 2x2 for the assisted arm.
Numerical result	AI-assisted readers: sensitivity=0.81; specificity approximately 0.97 (reported reader-study operating point).
Selection rule	Primary or most clinically relevant test-set estimate matching the assigned synthesis question; alternative models/thresholds were not treated as independent studies.
Sources	Flow: PDF pp. 2, 3; estimate selection: PDF pp. 3, 4

Signal	Response	Official signaling question
P1	PY	Was a single-gate design used?
P2	Y	Were participants prospectively enrolled?
P3	PN	Was a consecutive or random sample of participants included?
P4	N	Is the study group a representative sample of the intended-use population?
I1	Y	Was the index test conducted and interpreted according to the recommended instructions?
I2	Y	Were the index test results interpreted without knowledge of the reference standard results?
I3	Y	Were the index test results interpreted with the same information as would be available when the test is used in practice?
I4	Y	If an index test threshold was used, was it standard or pre-specified?
T1	PY	Does the reference standard adequately identify those with and without the target condition?
T2	Y	Was the target condition assessed in all participants?
T3	Y	Was the target condition assessed in the same way in all participants?
T4	Y	Did the reference standard avoid incorporating the index test?
T5	PY	Was the reference standard conducted and interpreted according to the recommended instructions?
T6	NI	Were the reference standard results interpreted without knowledge of the index test results?
T7	PY	If a reference standard threshold was used, was it standard or pre-specified?
T8	Y	Was there an appropriate interval between index test and reference standard?
A1	Y	Were all enrolled participants included in the analysis?
A2	PY	Were missing data handled appropriately?
A3	Y	Does the unit of analysis match the ideal test accuracy trial?
A4	Y	Were the estimates of sensitivity and specificity calculated appropriately?

Domain	Risk of bias	Applicability	Rationale	Source
Participants	High	High	Cluster-randomized cross-over controlled trial evaluating the effect of an AI-based decision-support system (dentalXrai Pro) on proximal caries detection by dentists using bitewing radiographs. Test set: 140 radiographs (used across dentists; not a classical test set). Selection, enrichment, case-control construction, exclusions, or limited spectrum could materially bias accuracy.	PDF pp. 2, 3

Domain	Risk of bias	Applicability	Rationale	Source
Index test	Low	Low	Index test: dentalXrai Pro (v1.0.4), a commercial system integrating: CNN-based tooth detection Pixelwise caries segmentation Automated report generation. A locked test evaluation and sufficiently prespecified interpretation were reported.	PDF pp. 2, 3
Target condition	II	II	Reference standard: 4 independent dento-maxillofacial radiologists 1 expert restorative dentist Pixelwise annotation on all 140 images Consensus refinement by fifth expert Lesion depth classified as: enamel, early dentin, advanced dentin . Blinding, calibration, reliability, or threshold details were insufficiently reported; missing supplements were not imputed.	PDF pp. 2, 4
Analysis	Low	Not applicable	The cluster-randomised crossover analysis explicitly applied a design effect for repeated readings; this was judged appropriate for the selected assisted-reading estimate.	PDF pp. 3, 4
Overall	High	High	Overall risk of bias followed the prespecified rule: any High domain yields an overall High judgment; all Low yields Low; otherwise II. Overall applicability reflects the three applicability domains.	Derived from the domain judgments above

Source-status note: Full-text article reviewed. NI was used wherever the article did not report sufficient information.

S4.6.28. Devlin 2021 (Q2)

Assessment element	Details
Design	Randomized controlled comparative diagnostic accuracy study evaluating dentists' ability to detect enamel-only proximal caries on bitewing radiographs with vs. without assistance from the AssistDent artificial intelligence software.
Source-to-analysis flow	Source cohort/data set (Randomized controlled comparative diagnostic accuracy study evaluating dentists' ability to detect enamel-only proximal caries on bitewing radiographs with vs. without assistance from the AssistDent artificial intelligence software.) → eligibility/selection as reported → development split(s), if applicable → selected independent/cross-validated test set: 103 images (model evaluation) → diagnostic analysis at Individual proximal tooth surfaces. level. Any unreported exclusions or indeterminate/missing observations were coded NI in Phase 5.
Reported evaluation set	103 images (model evaluation)
Analysis unit	Individual proximal tooth surfaces.
Selected estimate	Two randomised arms; the AI-assisted arm was extracted because it is the arm in which the index test is applied. Analysed separately as clinician-plus-AI.
Numerical result	TP=591; FP=422; FN=189; TN=2470; sensitivity=0.758; specificity=0.854
Selection rule	Primary or most clinically relevant test-set estimate matching the assigned synthesis question; alternative models/thresholds were not treated as independent studies.
Sources	Flow: PDF pp. 1, 2; estimate selection: PDF pp. 3, 4

Signal	Response	Official signaling question
P1	N	Was a single-gate design used?
P2	Y	Were participants prospectively enrolled?
P3	N	Was a consecutive or random sample of participants included?
P4	N	Is the study group a representative sample of the intended-use population?
I1	Y	Was the index test conducted and interpreted according to the recommended instructions?
I2	Y	Were the index test results interpreted without knowledge of the reference standard results?
I3	Y	Were the index test results interpreted with the same information as would be available when the test is used in practice?
I4	Y	If an index test threshold was used, was it standard or pre-specified?
T1	PY	Does the reference standard adequately identify those with and without the target condition?
T2	Y	Was the target condition assessed in all participants?
T3	Y	Was the target condition assessed in the same way in all participants?
T4	Y	Did the reference standard avoid incorporating the index test?
T5	Y	Was the reference standard conducted and interpreted according to the recommended instructions?
T6	PY	Were the reference standard results interpreted without knowledge of the index test results?
T7	Y	If a reference standard threshold was used, was it standard or pre-specified?
T8	Y	Was there an appropriate interval between index test and reference standard?
A1	Y	Were all enrolled participants included in the analysis?
A2	Y	Were missing data handled appropriately?
A3	Y	Does the unit of analysis match the ideal test accuracy trial?
A4	N	Were the estimates of sensitivity and specificity calculated appropriately?

Domain	Risk of bias	Applicability	Rationale	Source
Participants	High	High	Randomized controlled comparative diagnostic accuracy study evaluating dentists' ability to detect enamel-only proximal caries on bitewing radiographs with vs. without assistance from the AssistDent artificial intelligence software. Test set: 103 images (model evaluation). Selection, enrichment, case-control construction, exclusions, or limited spectrum could materially bias accuracy.	PDF pp. 1, 2

Domain	Risk of bias	Applicability	Rationale	Source
Index test	Low	Low	Index test: AssistDent (commercial proprietary ML-based software). Specific algorithmic architecture: NR Known functions: Automated detection of enamel-only proximal caries On-screen arrow prompts indicating suspected lesion locations. A locked test evaluation and sufficiently prespecified interpretation were reported.	PDF pp. 2, 4
Target condition	Low	High	Reference standard: Gold standard annotations produced by: Five dento-maxillofacial radiologists One professor of restorative dentistry Each image annotated independently by ≥3 experts Any enamel-only proximal caries marked by any expert was retained in consensus Duplicates removed Result: 1,972 enamel-only proximal caries in gold-standard annotation set. The reference procedure was judged sufficiently appropriate and independent for the study-defined target condition.	PDF pp. 1, 3
Analysis	High	Not applicable	The same enriched image set was read by multiple dentists, but the analysis used participant-level means/t tests without modelling the crossed image-by-reader dependence.	PDF pp. 3, 4
Overall	High	High	Overall risk of bias followed the prespecified rule: any High domain yields an overall High judgment; all Low yields Low; otherwise II. Overall applicability reflects the three applicability domains.	Derived from the domain judgments above

Source-status note: Full-text article reviewed. NI was used wherever the article did not report sufficient information.

S4.7. Signaling items coded as no information

Interpretation: The entries below identify every NI response and the reason no favorable method was inferred. NI denotes absence of sufficient reporting, not confirmation that the study method was inadequate.

Estimate	Signal	Domain	Official question	Reason for NI	Study ID
Ahmed 2025	P3	Participants	Was a consecutive or random sample of participants included?	The full-text article did not report enough information and no supplementary file was available; no favorable method was inferred.	Ahmed_2025
Bayraktar 2022	T6	Target	Were the reference standard results interpreted without knowledge of the index test results?	The full-text article did not report enough information and no supplementary file was available; no favorable method was inferred.	Bayraktar_2022
Biltekin 2025	T6	Target	Were the reference standard results interpreted without knowledge of the index test results?	The full-text article did not report enough information and no supplementary file was available; no favorable method was inferred.	Biltekin_2025
Cao 2025	T6	Target	Were the reference standard results interpreted without knowledge of the index test results?	The full-text article did not report enough information and no supplementary file was available; no favorable method was inferred.	Cao_2025
Estai 2022	T6	Target	Were the reference standard results interpreted without knowledge of the index test results?	The full-text article did not report enough information and no supplementary file was available; no favorable method was inferred.	Estai_2022
Orhan 2023	P3	Participants	Was a consecutive or random sample of participants included?	The full-text article did not report enough information and no supplementary file was available; no favorable method was inferred.	Orhan_2023
Orhan 2023	I4	Index	If an index test threshold was used, was it standard or pre-specified?	The full-text article did not report enough information and no supplementary file was available; no favorable method was inferred.	Orhan_2023
Orhan 2023	A2	Analysis	Were missing data handled appropriately?	The full-text article did not report enough information and no supplementary file was available; no favorable method was inferred.	Orhan_2023
Park 2022	T6	Target	Were the reference standard results interpreted without knowledge of the index test results?	The full-text article did not report enough information and no supplementary file was available; no favorable method was inferred.	Park_2022
Park 2023	T6	Target	Were the reference standard results interpreted without knowledge of the index test results?	The full-text article did not report enough information and no supplementary file was available; no favorable method was inferred.	Park_2023
Park 2023	A2	Analysis	Were missing data handled appropriately?	The full-text article did not report enough information and no supplementary file was available; no favorable method was inferred.	Park_2023
Chaudhari 2025	P3	Participants	Was a consecutive or random sample of participants included?	The full-text article did not report enough information and no supplementary file was available; no favorable method was inferred.	Chaudhari_2025
Chaudhari 2025	I4	Index	If an index test threshold was used, was it standard or pre-specified?	The full-text article did not report enough information and no supplementary file was available; no favorable method was inferred.	Chaudhari_2025
Chaudhari 2025	T2	Target	Was the target condition assessed in all participants?	The full-text article did not report enough information and no supplementary file was available; no favorable method was inferred.	Chaudhari_2025
Chaudhari 2025	T3	Target	Was the target condition assessed in the same way in all participants?	The full-text article did not report enough information and no supplementary file was available; no favorable method was inferred.	Chaudhari_2025
Chaudhari 2025	T6	Target	Were the reference standard results interpreted without knowledge of the index test results?	The full-text article did not report enough information and no supplementary file was available; no favorable method was inferred.	Chaudhari_2025
Chaudhari 2025	T7	Target	If a reference standard threshold was used, was it standard or pre-specified?	The full-text article did not report enough information and no supplementary file was available; no favorable method was inferred.	Chaudhari_2025
Chaudhari 2025	A2	Analysis	Were missing data handled appropriately?	The full-text article did not report enough information and no supplementary file was available; no favorable method was inferred.	Chaudhari_2025
Caldwell 2026	P3	Participants	Was a consecutive or random sample of participants included?	The full-text article did not report enough information and no supplementary file was available; no favorable method was inferred.	Caldwell_2026
Chen 2026	T6	Target	Were the reference standard results interpreted without knowledge of the index test results?	The full-text article did not report enough information and no supplementary file was available; no favorable method was inferred.	Chen_2026

Estimate	Signal	Domain	Official question	Reason for NI	Study ID
van Nistelrooij 2026	I4	Index	If an index test threshold was used, was it standard or pre-specified?	The full-text article did not report enough information and no supplementary file was available; no favorable method was inferred.	VanNistelrooij_2026
van Nistelrooij 2026	T6	Target	Were the reference standard results interpreted without knowledge of the index test results?	The full-text article did not report enough information and no supplementary file was available; no favorable method was inferred.	VanNistelrooij_2026
Rahimi 2026	I4	Index	If an index test threshold was used, was it standard or pre-specified?	The full-text article did not report enough information and no supplementary file was available; no favorable method was inferred.	Rahimi_2026
Rahimi 2026	T6	Target	Were the reference standard results interpreted without knowledge of the index test results?	The full-text article did not report enough information and no supplementary file was available; no favorable method was inferred.	Rahimi_2026
Aarti Rajambigai 2026	P3	Participants	Was a consecutive or random sample of participants included?	The full-text article did not report enough information and no supplementary file was available; no favorable method was inferred.	AartiRajambigai_2026
Aarti Rajambigai 2026	P4	Participants	Is the study group a representative sample of the intended-use population?	The full-text article did not report enough information and no supplementary file was available; no favorable method was inferred.	AartiRajambigai_2026
Aarti Rajambigai 2026	I1	Index	Was the index test conducted and interpreted according to the recommended instructions?	The full-text article did not report enough information and no supplementary file was available; no favorable method was inferred.	AartiRajambigai_2026
Aarti Rajambigai 2026	I4	Index	If an index test threshold was used, was it standard or pre-specified?	The full-text article did not report enough information and no supplementary file was available; no favorable method was inferred.	AartiRajambigai_2026
Aarti Rajambigai 2026	T7	Target	If a reference standard threshold was used, was it standard or pre-specified?	The full-text article did not report enough information and no supplementary file was available; no favorable method was inferred.	AartiRajambigai_2026
Aarti Rajambigai 2026	A2	Analysis	Were missing data handled appropriately?	The full-text article did not report enough information and no supplementary file was available; no favorable method was inferred.	AartiRajambigai_2026
Fadilah 2026	I2	Index	Were the index test results interpreted without knowledge of the reference standard results?	The full-text article did not report enough information and no supplementary file was available; no favorable method was inferred.	Fadilah_2026
Fadilah 2026	I4	Index	If an index test threshold was used, was it standard or pre-specified?	The full-text article did not report enough information and no supplementary file was available; no favorable method was inferred.	Fadilah_2026
Fadilah 2026	T6	Target	Were the reference standard results interpreted without knowledge of the index test results?	The full-text article did not report enough information and no supplementary file was available; no favorable method was inferred.	Fadilah_2026
Fadilah 2026	A2	Analysis	Were missing data handled appropriately?	The full-text article did not report enough information and no supplementary file was available; no favorable method was inferred.	Fadilah_2026
Wang 2026	P3	Participants	Was a consecutive or random sample of participants included?	The full-text article did not report enough information and no supplementary file was available; no favorable method was inferred.	Wang_2026
Liu 2026	T6	Target	Were the reference standard results interpreted without knowledge of the index test results?	The full-text article did not report enough information and no supplementary file was available; no favorable method was inferred.	Liu_2026
Parekh 2026	I4	Index	If an index test threshold was used, was it standard or pre-specified?	The full-text article did not report enough information and no supplementary file was available; no favorable method was inferred.	Parekh_2026
Bicengil 2026	P3	Participants	Was a consecutive or random sample of participants included?	The full-text article did not report enough information and no supplementary file was available; no favorable method was inferred.	Bicengil_2026
Abdelaziz 2026	P3	Participants	Was a consecutive or random sample of participants included?	The full-text article did not report enough information and no supplementary file was available; no favorable method was inferred.	Abdelaziz_2026
Abdelaziz 2026	T6	Target	Were the reference standard results interpreted without knowledge of the index test results?	The full-text article did not report enough information and no supplementary file was available; no favorable method was inferred.	Abdelaziz_2026
Kang 2026	T6	Target	Were the reference standard results interpreted without knowledge of the index test results?	The full-text article did not report enough information and no supplementary file was available; no favorable method was inferred.	Kang_2026
Ren 2026	P3	Participants	Was a consecutive or random sample of participants included?	The full-text article did not report enough information and no supplementary file was available; no favorable method was inferred.	Ren_2026

Estimate	Signal	Domain	Official question	Reason for NI	Study ID
Ren 2026	I2	Index	Were the index test results interpreted without knowledge of the reference standard results?	The full-text article did not report enough information and no supplementary file was available; no favorable method was inferred.	Ren_2026
Ren 2026	I4	Index	If an index test threshold was used, was it standard or pre-specified?	The full-text article did not report enough information and no supplementary file was available; no favorable method was inferred.	Ren_2026
Ren 2026	A2	Analysis	Were missing data handled appropriately?	The full-text article did not report enough information and no supplementary file was available; no favorable method was inferred.	Ren_2026
Mertens 2021	T6	Target	Were the reference standard results interpreted without knowledge of the index test results?	The full-text article did not report enough information and no supplementary file was available; no favorable method was inferred.	Mertens_2021

S4.8. Auditability statement

QUADAS-3 was applied at the numerical-estimate level. Arsiwala-Scheppach et al. (2024) was treated as an ancillary report of the Mertens et al. (2021) reader trial and was not assigned a separate estimate-level judgment. The assessment used the full-text reports available to the review team. Supplementary files of the primary studies were unavailable and their contents were not inferred; no-information responses were retained whenever reporting was insufficient.

S4.9. Independent source verification and consensus

The final master workbook preserves the two independently completed source-verification records, the automated comparison, and the adjudication register. Verification was completed using the effective review date; no retrospective dates were used.

Reviewer/component	Initials	Date	Estimates completed	Result
Source verifier 1	ACG	26 August 2026	28/28	Complete
Source verifier 2	JM	26 August 2026	28/28	Complete
Matrix comparison	—	26 August 2026	28/28	All pre-populated judgments confirmed; not interpreted as a reliability statistic
Adjudication	ISG	Not required	0	No disagreements required adjudication
